# C-terminal evolutionary remodelling of isoleucyl-tRNA synthetases is a prokaryote-specific strategy for tuning aminoacylation rate

**DOI:** 10.1038/s41467-026-78241-1

**Published:** 2026-10-12

**Authors:** Petra Modrusan, Alojzije Brkic, Andre Lecona Buttelli, Marc Leibundgut, Igor Zivkovic, Nenad Ban, Liam M. Longo, Ita Gruic-Sovulj

**Affiliations:** 1 University of Zagreb Faculty of Science, Department of Chemistry, Zagreb, Croatia; 2 Earth-Life Science Institute, Institute of Science Tokyo, Tokyo, Japan; 3 Department of Biology, Institute of Molecular Biology and Biophysics, ETH Zürich, Zürich, Switzerland; 4 Blue Marble Space, Seattle, WA USA

**Keywords:** Enzyme mechanisms, Molecular evolution, X-ray crystallography

## Abstract

Aminoacyl-tRNA synthetases are the guardians of translational fidelity. Their complex function is mirrored by an elaborate structure, which includes multiple nested domains. While the evolutionary pressures that promoted the emergence of some domains, such as the editing domain, are clear, the pressures acting on other domains, particularly those at the C-terminus, are not. Here, we combine kinetic analysis, X-ray crystallography, and bioinformatics to unveil the history and evolutionary forces that have shaped isoleucyl-tRNA synthetase (IleRS) domain structure. We find that the traditional classification of IleRSs based on the C-terminal tRNA-recognition domains is incomplete, as it fails to capture features of the synthetic domain. Guided by a crystal structure of the *Priestia megaterium* IleRS2:tRNA^Ile^ complex, we removed key interactions between IleRS2 and its cognate tRNA and characterised their impact on enzyme activity. We found that D-loop interactions with the IleRS2 C-terminal domains are non-essential in prokaryotes, and their loss can even increase catalytic turnover. Further, we show that the zinc-binding domain of IleRS1 recognises the anticodon less stringently than the canonical C-terminal domain of IleRS2. Our data suggest that C-terminal evolutionary remodelling of IleRSs is an ongoing process with a historical precedent and is consistent with selection for faster aminoacylation rate.

## Abstract (web summary)

Aminoacyl-tRNA synthetases decipher the genetic code with remarkable speed and accuracy. Here, the authors show how evolution can increase aminoacylation speed by repeatedly reshaping the interactions between an enzyme and its tRNA substrate.

## Introduction

Aminoacyl-tRNA synthetases (AARSs) are indispensable enzymes that establish the genetic code by coupling amino acids and their cognate tRNAs for ribosomal protein synthesis^1,2^. As guardians of translational accuracy, AARSs are under strong evolutionary pressure for high selectivity in amino acid^3^ and tRNA recognition^4^. In cases where initial amino acid selectivity alone could not achieve the required translation accuracy, AARSs evolved hydrolytic editing^2,5^ to prevent error propagation.

**Fig. 1:**
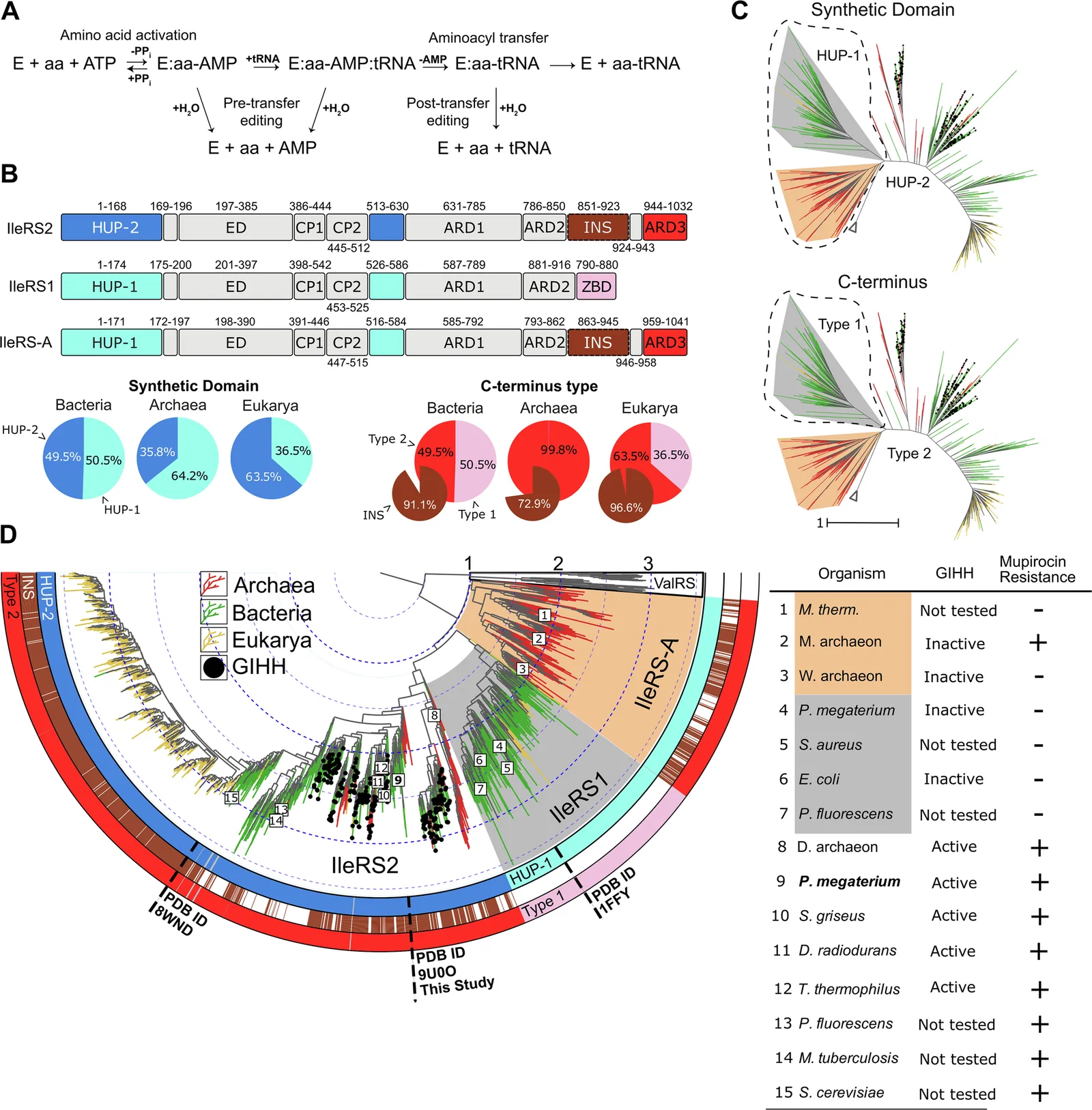
Reaction mechanisms, domain composition, and phylogeny of IleRS. **A** The IleRS reaction cycle. An amino acid is first activated to form an aminoacyl-adenylate intermediate, followed by aminoacyl transfer to the cognate tRNA within the synthetic site of the HUP (which stands for HIGH-signature proteins, UspA, and PP-ATPases) domain. If a non-cognate amino acid is activated, it can be cleared within the synthetic site or transferred to tRNA. Mischarged tRNA is rapidly deacylated at the editing domain (ED). **B** Domain composition for the three primary IleRS groups, which form distinct evolutionary clades. Each group consists of a HUP domain, divided by two connective peptides (CP) and an editing domain (ED), followed by tRNA anticodon-recognition domains (ARD1–ARD3, ZBD). IleRS-A has a HUP domain that is more closely related to that of IleRS1 (termed HUP-1) but shares a type 2 C-terminal domain organisation with IleRS2, which comprises ARD1–ARD3 and, frequently, an ~80-amino-acid insertion within the ARD2 domain (INS). In contrast, IleRS1 has a treble clef zinc-binding domain (ZBD) and lacks INS, which is referred to as the type 1 C-terminal organisation. The indicated residue ranges are for IleRS1 and IleRS2 from *P. megaterium* and IleRS-A from *M. thermoautotrophicus*. The phylogenetic distributions of HUP and C-terminal domain types are presented as pie charts following the same colour scheme as the domains (HUP-1: cyan; HUP-2: blue; ARD3: red; ZBD: pink; INS: brown). **C** Unrooted maximum-likelihood phylogenetic tree of the HUP domain. Branches are coloured according to the domain of life (red: Archaea; green: Bacteria; yellow: Eukarya). The clades are coloured according to IleRS groups (orange: IleRS-A; grey: IleRS1; white: IleRS2). ValRS sequences are indicated by a collapsed clade (white triangle). In the top and bottom trees, the clades corresponding to the HUP-1 domain type and the type 1 C-terminal architecture are circled for clarity. **D** Outgroup-rooted maximum-likelihood tree of the IleRS HUP domain. Tree branches and clades are coloured as in (C). Black circles at the leaf tips indicate the presence of the non-canonical HIGH motif variant (GIHH), which has been shown to confer mupirocin hyper-resistance in IleRS2. From innermost to outermost, the rings annotate the HUP type, the presence of an INS subdomain, and the type of C-terminal organisation, respectively, following the same colour scheme as in (B). IleRS enzymes with either natural (*S. griseus* and *D. radiodurans*) or introduced GIHH motifs whose activities have been tested^22^ (Supplementary Fig. 4) are indicated by numbers in the phylogenetic tree, with the corresponding organisms listed in the adjacent table. IleRS enzymes for which sensitivity to mupirocin has been experimentally assessed^15,20–22,25–29^ (Supplementary Fig. 4) are also indicated. HUP-based phylogenetic trees, including the underlying sequence sets and alignments used to build them, are deposited in the Zenodo repository under https://doi.org/10.5281/zenodo.21453230^61^.

AARSs are divided into two evolutionarily distinct classes, I and II, each comprising ten members^6,7^. Isoleucyl-tRNA synthetase (IleRS) is a class I enzyme responsible for charging tRNA^Ile^ with isoleucine (Ile)^8^ (Fig. 1A). IleRS enzymes catalyse an ATP-dependent two-step aminoacylation reaction, in which the amino acid is first activated as an adenylate and then transferred to its cognate tRNA. Both of these steps occur within the synthetic site of the HUP (which stands for HIGH-signature proteins, UspA, and PP-ATPases) domain^9^ (Fig. 1B). When IleRS fails to reject a non-cognate amino acid resembling Ile at the activation step, the enzyme either clears the misactivated amino acid within the HUP domain^10,11^ or hydrolyses the mischarged tRNA^Ile^ at the editing domain (ED)^12,13^ (Fig. 1A, B).

IleRSs are traditionally subdivided into two groups, IleRS1 and IleRS2, based on the distinct anticodon-recognition domains (ARDs) at their C-termini^14–17^, although this classification does not fully reflect their evolutionary relationships (Fig. 1B). IleRS1 enzymes have a C-terminal zinc-binding treble-clef motif^18^ (ZBD) and are found only in bacteria and eukaryotic organelles^19^. In contrast, IleRS2 enzymes, present in archaea, the eukaryotic cytoplasm, and many bacteria^15,20,21^, have a C-terminal ARD3 domain that is structurally homologous to the preceding domain (ARD2)^17,22^. IleRS2 can also have a unique insertion in the ARD2 domain termed INS.

IleRS1 and IleRS2 also differ in their sensitivity to mupirocin, a naturally occurring and clinically relevant polyketide antibiotic^23^ that binds to the synthetic site of the HUP domain^24^. Mupirocin strongly inhibits IleRS1^25–28^, whereas IleRS2 from eukaryotes^25,29^ and bacteria^15,21,26,27^ display significantly greater mupirocin resistance, often requiring >10^3^ higher concentrations to be inactivated. In addition, IleRS2 may harbour a natural, non-canonical variant of the HIGH motif, a signature of class I AARSs, that confers hyper-resistance to mupirocin^22^.

The fact that IleRS2 in bacteria is a housekeeping enzyme that also confers antibiotic (hyper)resistance raises the question of why IleRS1 has persisted in some bacterial lineages. A clue may be provided by the study of *Priestia megaterium*, one of the rare organisms that has both IleRS1 and IleRS2. Previously, we found that the IleRS1 of *P. megaterium* has a higher aminoacylation turnover rate and supports faster in vitro translation than its IleRS2^26^. Consistent with this observation, an analysis of reported bacterial doubling times suggested that faster-growing bacteria preferentially employ IleRS1^26^. Could the increase in turnover be related to the presence of ZBD and/or the absence of INS in IleRS1?

To address this question and better understand the roles of ARD3, ZBD, and INS, we solved the crystal structure of *P. megaterium* IleRS2 in complex with tRNA^Ile^ and carried out a detailed kinetic characterisation of IleRS and tRNA^Ile^ mutants. We also performed a phylogenetic analysis of archaeal, bacterial, and eukaryotic IleRS enzymes.

Here, we show an updated IleRS phylogeny with a clade of deep-branching archaeal IleRSs (termed IleRS-A) in addition to the two previously recognised clades, bacterial IleRS1 and archaeal/bacterial/eukaryotic IleRS2. Our phylogenetic analysis indicates that IleRS1 diverged from an IleRS-A-like ancestor, likely driven by a replacement of a C-terminal domain. We also find evidence for ongoing C-terminal remodelling, with a concerted loss of the INS subdomain across the prokaryotic tree of life. We show that *P. megaterium* IleRS2 recognises the anticodon bases with slightly higher fidelity than *P. megaterium* IleRS1, and that INS, present exclusively in the ARD2 domain of IleRS2 and IleRS-A, first served as a tRNA-binding domain in archaea and bacteria but later evolved into a tRNA recognition element in eukaryotes. Finally, we retrace the evolutionary loss of the INS, showing that it is a relatively facile evolutionary transition. Our data frame the replacement of ARD3 by ZBD to form the IleRS1 clade, as well as the ongoing loss of INS, as prokaryotic strategies for increased aminoacylation turnover.

## Results

### Resolving a missing deep-branching archaeal IleRS clade

The traditional subdivision of IleRSs based on differences in their C-terminal domains has generally been interpreted as reflecting an evolutionary split into two clades: IleRS1 found in bacteria versus IleRS2 found in archaea, some bacteria, and the cytoplasm of eukaryotes^19^. However, deeper phylogenetic analysis revealed that some archaeal IleRSs, so far attributed to the IleRS2 clade, actually cluster more closely with IleRS1^21,22^. This observation raises the question of whether C-terminal domain types correspond to a clade separation.

To clarify the evolutionary history of IleRS, we constructed a database of 97,678 IleRS sequences spanning bacteria, archaea, and eukaryotes and annotated their protein domain architectures (see Methods and Supplementary Fig. 1 for more details). Phylogenetic trees were calculated for each of the core structural domains (HUP, ED, CP1 (connective peptide 1), CP2, and ARD1; Fig. 1B) and rooted with ValRS, the closest homologue of IleRS (Supplementary Fig. 2A), as the outgroup. For each structural domain, representative sequences were re-sampled with replacement five times, aligned, and phylogenetic trees were inferred (see Methods for more details). Unrooted and outgroup-rooted trees of the IleRS synthetic (HUP) domain are presented in Fig. 1C and D, respectively, while trees calculated using other structural domains are presented in Supplementary Fig. 3.

Foremost, we find that the standard two-group division scheme does not conform to the clade structure of the phylogenetic tree. In addition to IleRS1 and IleRS2, we identify a previously undiscovered clade of archaeal IleRS (IleRS-A). This clade has a C-terminal domain organisation like IleRS2 but has a HUP domain that forms a clade of its own and is more closely related to that of IleRS1 (HUP-1; Fig. 1C, D). Indeed, the closer resemblance of IleRS-A to IleRS1 is consistent across phylogenetic trees inferred from the HUP domain, as well as trees calculated from other core domains (Supplementary Fig. 3). Plotting the occurrence of the primary non-canonical HIGH motif, referred to here as GIHH, demonstrates that it is a feature of HUP-2^22^ but is entirely absent from IleRS-A and IleRS1 (Fig. 1D). In the GIHH motif, the highly conserved first and third amino acid positions of the canonical HIGH motif are swapped. Note that sequence variability exists within both motifs; thus, these terms are used herein as group names rather than exact sequences.

To determine whether our updated classification scheme meaningfully relates to properties of the synthetic domain, we identified three archaeal enzymes for biochemical characterisation: the IleRS-As from Woesearchaeota (WaIleRS-A) and *Candidatus* Micrarchaeota (MaIleRS-A), and the IleRS2 from *Candidatus* Diapherotrites (DaIleRS2). Based on the properties of the characterised HUP-1 synthetic domains to date^22,25–28^, we predict that IleRS-A enzymes should (a) not tolerate the non-canonical GIHH motif and (b) exhibit sensitivity to mupirocin. As expected, both WaIleRS-A and MaIleRS-A were fully inactivated when the 1^st^ and 3^rd^ positions of their respective HIGH motifs were swapped (Supplementary Fig. 4A, B), whereas DaIleRS2 exhibited only a twofold lower rate of Ile activation (Supplementary Fig. 4C), similar to that previously reported for IleRS2 enzymes^22^.

Next, we tested whether these archaeal enzymes responded to mupirocin as expected. As observed for various IleRS1s^25–28^ and the IleRS-A from *Methanothermobacter thermoautotrophicus*^20^, we found that WaIleRS-A is sensitive to mupirocin (Supplementary Fig. 4D). Likewise, DaIleRS2 was resistant to mupirocin (Supplementary Fig. 4D), as expected for an IleRS2^15,21,22,27,29^. Interestingly, MaIleRS-A was also resistant to high µM concentrations of mupirocin (Supplementary Fig. 4D). This unexpected result suggests greater variability in mupirocin resistance than expected. Nevertheless, our biochemical data generally support the assignment of the IleRS-A and IleRS1 clades as having biochemically similar synthetic domains, which we refer to here as the HUP-1 synthetic domain (Fig. 1).

For nearly all trees from all protein domains (Fig. 1 and Supplementary Fig. 3), the ValRS-outgroup-inferred root is positioned closest to, or within, IleRS-A. Although the position of IleRS1 with respect to IleRS-A genes is not entirely stable, all trees suggest that IleRS1 emerged from an IleRS-A-like ancestor. Although ValRS is more closely related to IleRS (Supplementary Fig. 2A), and thus the natural choice for an outgroup, we also considered whether LeuRS-rooted trees similarly position IleRS-A as the primal IleRS form (Supplementary Fig. 2B). Owing to its greater evolutionary distance, the position of the root when using LeuRS as an outgroup was less stable, although reliably localised in the HUP-1 clade (IleRS1 or IleRS-A). Taken together with the stable ValRS rooting closest to IleRS-A, we conclude that the last common ancestor of contemporary IleRS synthetic domains was IleRS-A-like. Subsequent remodelling of the C-terminus likely occurred concomitant with, or in close temporal proximity to, the emergence of IleRS1, likely in a bacterial ancestor. Eukaryotic IleRS1 (in organelles) and IleRS2 genes then evolved independently from bacterial ancestors. Thus, current evidence supports a model (Supplementary Fig. 5) in which IleRS evolution involved two independent passages from archaeal to bacterial to eukaryotic parent organisms.

### C-terminal domain exchange and loss have shaped and are still shaping IleRS evolution

The phylogenetic tree shows that remodelling of the C-terminus is a core driver of IleRS diversification. Two key examples emerge: exchange of ARD3 with an unrelated ZBD fold and loss of the INS subdomain ( ~ 80 residues). As the ZBD domain is somewhat shorter and less complex than the ARD3 domain (31 vs. 82 residues on average, respectively), both processes result in a modest shortening of IleRS. However, whereas ARD3 exchange appears to have occurred just once, relatively early in the evolution of contemporary IleRS proteins, INS subdomain loss appears to be an actively ongoing process: we find that a number of organisms have lost the INS subdomain independently in both the IleRS-A and IleRS2 clades, with the IleRS2 eukaryote sub-clade being a notable exception (Fig. 1D).

Why are the C-terminal domains of IleRS a hotspot for diversification, and what forces drove, and are still driving, these changes? To address these questions, we sought to (a) identify the residues of ARD3 and INS domains that interact with tRNA^Ile^; (b) evaluate the catalytic consequences of these interactions; and (c) experimentally realise an event of C-terminal remodelling.

### tRNA^Ile^ recognition by bacterial IleRS2

**Fig. 2:**
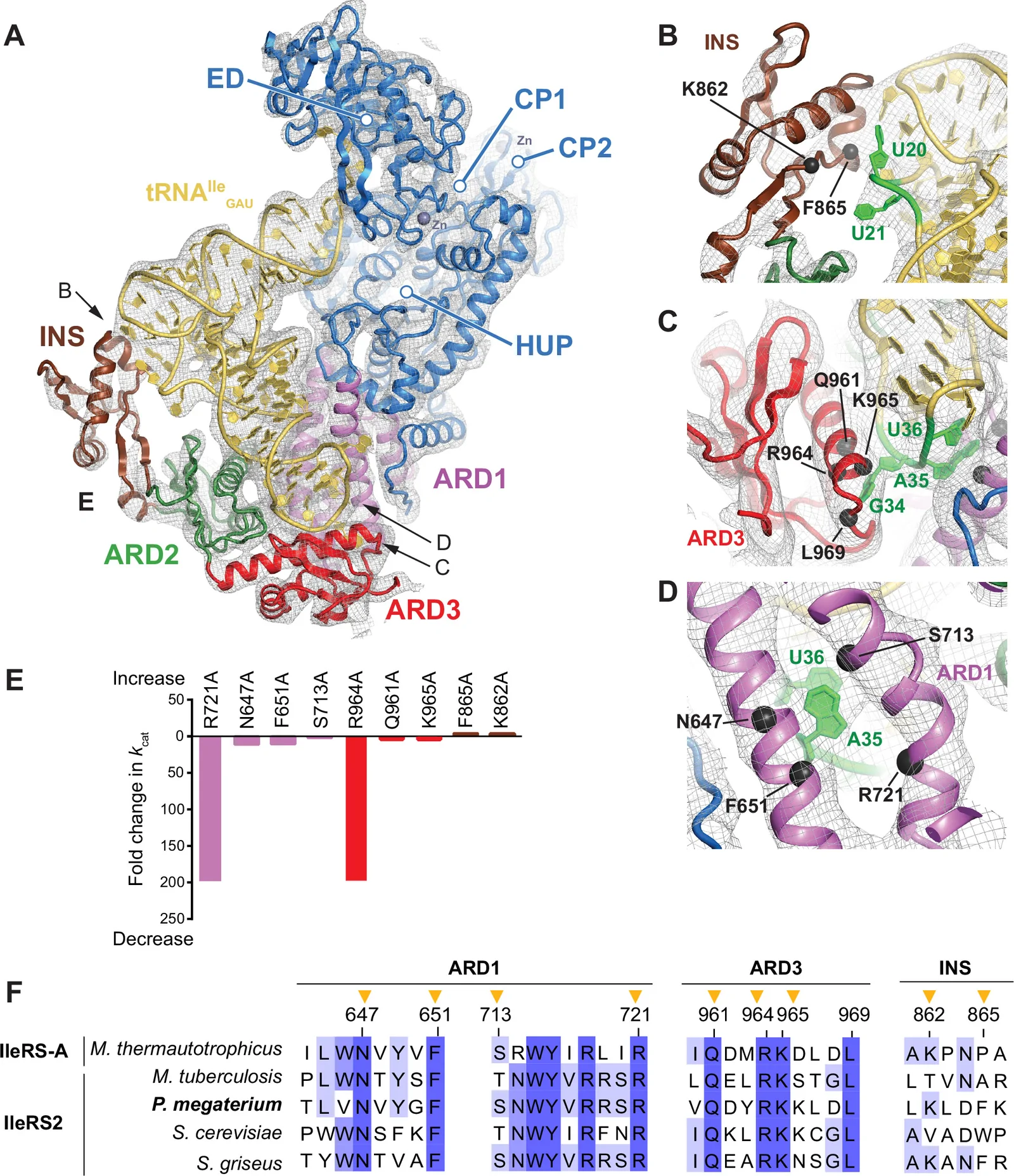
tRNA^Ile^ recognition by *Pm*IleRS2. **A** Structure of the *Pm*IleRS2:*Ec*tRNA^Ile^:ATP complex. As the precise interactions between amino acid side chains and tRNA^Ile^ were not resolved, the participation of the residues (depicted as C*α-*centred spheres) in tRNA^Ile^ recognition was instead inferred from comparison with the related high-resolution crystal structure from yeast (PDB: 8WND^17^; Supplementary Fig. 12) and corroborated by detailed kinetic analysis (Table 1). *Pm*IleRS2 is coloured according to domain borders (HUP, CP1, CP2 and ED: blue; ARD1: violet; ARD2: green; ARD3: red; INS: brown), and the 2m*F*_o_–D*F*_c_ Fourier difference map (grey mesh) is carved 4.6 Å around the model and contoured at 1.0 *σ*. tRNA^Ile^ is coloured yellow. **B–D** D-loop and anticodon recognition by *Pm*IleRS2. The position of the INS subdomain insertion into ARD2 suggests interactions with the D-loop in the vicinity of U20 and U21 nucleotides (**B**). The pocket formed by residues R964, L969, Q961, and K965 within ARD3 is found in proximity to the first anticodon base, G34 (**C**), while the second and third bases, A35 and U36, are close to residues R721, N647, F651, and S713 in the ARD1 subdomain (**D**). **E** Kinetic analysis of the participation of these residues in tRNA^Ile^ recognition by mutagenesis (Table 1), with the fold change in *k*_cat_ relative to the WT plotted. Source data are provided as a Source Data file. **F** Sequence alignment showing that the residues involved in anticodon recognition in *Pm*IleRS2 are highly conserved, in contrast to those involved in D-loop recognition. Residues that are mutated and experimentally tested are marked with arrows.

Because eukaryotic INS subdomains are rarely lost, we reasoned that the role of the INS subdomain in eukaryotes may differ from that in bacteria or archaea. Thus, we solved the crystal structure of IleRS2 from *P. megaterium* (*Pm*IleRS2) bound to *Escherichia coli* tRNA^Ile^ and ATP (Fig. 2A, Supplementary Table 1). This crystal structure of a prokaryotic IleRS contains the more evolutionarily unstable INS subdomain in complex with tRNA^Ile^, making it a particularly useful point of comparison with the tRNA^Ile^-bound structure of yeast IleRS2^17^ and the tRNA^Ile^- and mupirocin-bound structure of *Staphylococcus aureus* IleRS1 (*Sa*IleRS1)^16^.

Despite a limited resolution of 5.3 Å (Supplementary Table 1), all domains of *Pm*IleRS2, as well as the bound tRNA^Ile^, were visible in the electron density of all four complexes in the asymmetric unit (Fig. 2, Supplementary Fig. 6, Supplementary Fig. 7). This allowed modelling of the protein and tRNA^Ile^ backbones using the structures of *Pm*IleRS2:Ile-AMS (PDB: 8C8V)^22^ and yeast IleRS2 bound to the tRNA^Ile^ and Ile (PDB: 8WND)^17^ as guides. After iterative readjustment and restrained refinement using non-crystallographic symmetry (NCS) averaging, the final model displayed high-quality metrics at the achieved resolution (Supplementary Table 1), supporting its accuracy and robustness. Further, the structural model was functionally verified by extensive mutagenesis and kinetic measurements (Fig. 2E, F).

In the *Pm*IleRS2:tRNA^Ile^ complex (Fig. 2A), the tRNA acceptor stem is captured in the editing site, and the anticodon loop is lodged within a cleft formed by the ARD1–ARD3 domains. The overall architecture is consistent with previous tRNA^Ile^-bound IleRS structures^16,17^ (Supplementary Fig. 8) and with a type 2 C-terminal domain organisation (Fig. 1B). When compared to the structure of *Pm*IleRS2 without tRNA^Ile^ (PDB: 8C8V)^22^, we find that tRNA^Ile^ binding promotes a ~ 77° rotation and ~28 Å displacement of the INS subdomain in all four complexes in the asymmetric unit (Supplementary Figs. 9, 10), enabling its engagement with the D-loop by motion around a hinge in the ARD2 domain (Supplementary Fig. 7, Supplementary Fig. 11). In this INS subdomain conformation, K862 and F865 become positioned close to bases U20 and U21 of the tRNA^Ile^, suggesting a role in phosphate backbone recognition (Fig. 2B).

To recognise the anticodon loop, our model indicates that ARD3 (Fig. 2A) may be involved in recognition of the first anticodon base, G34, through the pocket formed by the conserved residues Q961, R964, K965, and L969 (Fig. 2C), while the second and third anticodon nucleotides, A35 and U36, may be recognised by ARD1 residues R721, S713, F651 and N647 (Fig. 2D). In both cases, comparison with the high-resolution yeast IleRS2:tRNA^Ile^ structure supported the assignment of the recognition pockets (Supplementary Fig. 12).

To corroborate the structural data, we investigated the putative functional role of these residues in tRNA^Ile^ recognition by *Pm*IleRS2 using mutagenesis and steady-state kinetic analysis. Substitution of R964, shown to participate in G34 recognition in yeast (Supplementary Fig. 12) and possibly also in *Pm*IleRS2 (Fig. 2C), abolished the capacity of *Pm*IleRS2 to charge tRNA^Ile^ with Ile, confirming its key role in G34 recognition. The aminoacylation rate for the R964A mutant was below 0.001 s^−1^, demonstrating more than a 100-fold drop relative to the wild-type (WT) enzyme (Table 1). The barely detectable activity of the R964A mutant precluded the determination of a *K*_M_ (tRNA^Ile^). We next mutated two highly conserved ARD3 residues located near G34, Q961 and K965. The catalytic efficiency (*k*_cat_/*K*_M_) for the Q961A and K965A mutants decreased 10-fold relative to WT (Table 1), suggesting a supporting role in G34 recognition together with R964.

**Table 1:** Steady-state kinetic parameters for aminoacylation of wild-type and D-loop tRNA^Ile^ variants by wild-type *Pm*IleRS2 and its mutants.^a^

		*k*_cat_ / s^−1^	*K*_M_ (tRNA^Ile^) / µM	*k*_cat_ / *K*_M_ / s^−1^ µM^−1^	Relative change^*c*^ *k*_cat_ / *K*_M_
tRNA^Ile^					
	WT	0.19 ± 0.01	3.58 ± 0.30	0.052 ± 0.004	1
ARD1	R721A	<0.001	n.d.^*b*^	-	-
	N647A	0.020 ± 0.006	7.7 ± 1.9	0.0026 ± 0.0003	0.05
	F651A	0.017 ± 0.003	11.5 ± 1.7	0.0015 ± 0.0004	0.03
	S713A	0.09 ± 0.02	8.07 ± 2.08	0.0108 ± 0.0007	0.2
ARD3	Q961A	0.047 ± 0.003	7.4 ± 1.4	0.007 ± 0.001	0.13
	K965A	0.05 ± 0.01	11.9 ± 3.9	0.0049 ± 0.0007	0.09
	R964A	<0.001	n.d.^*b*^	-	-
INS	F865A	0.79 ± 0.09	35.8 ± 11.5	0.027 ± 0.005	0.52
	K862A	0.66 ± 0.09	33.6 ± 7.4	0.021 ± 0.003	0.40
	K862A_F865A	0.66 ± 0.16	33.8 ± 14.2	0.025 ± 0.003	0.48
	ΔINS	0.16 ± 0.05	48.9 ± 11.8	0.0032 ± 0.0003	0.06
tRNA^Ile^ U20G					
	WT	0.31 ± 0.07	8.5 ± 1.2	0.040 ± 0.009	0.8
INS	F865A	0.84 ± 0.06	28.8 ± 4.8	0.030 ± 0.004	0.6
	K862A	0.52 ± 0.04	11.3 ± 2.3	0.053 ± 0.016	1

^*a*^Reactions were performed using in vitro transcribed *Pm*tRNA^Ile^ GAU and its U20G variant as substrates.

^*b*^n.d. means not determined due to insufficient activity.

^*c*^Change relative to the wild-type enzyme *Pm*IleRS2 (mutant/wild-type).

Values represent the mean ± SEM of three (*n* = 3) independent experiments. Exceptions are Q961A, K965A and F865A, for which four (*n* = 4) independent experiments were conducted, and K862A_F865A, with five (*n* = 5) independent experiments. Data from each experiment were fitted individually, and the resulting values were used to calculate the mean ± SEM. Source data are provided as a Source Data file.

The next two anticodon bases, A35 and U36, may be recognised by the strictly conserved R721 within the ARD1 domain of *Pm*IleRS2 (Fig. 2D), as inferred by comparison with the yeast IleRS2 structure (Supplementary Fig. 12). Similar to R964A, the R721A mutation decreased the aminoacylation rate to below 0.001 s^−1^ (Table 1). Mutation of ARD1 residues N647, F651, and S713 to alanine (Fig. 2D) decreased the aminoacylation *k*_cat_/*K*_M_ by 5 to 30-fold, mainly via a *k*_cat_ effect (Table 1). These results demonstrate that R721 is critical for A35 and U36 recognition, with other residues in ARD1 further refining *Pm*IleRS2 specificity.

### Anticodon recognition by *Pm*IleRS2 is more stringent than by *Pm*IleRS1

Although IleRS1 and IleRS2 both utilise ARD1 and ARD3 to read the anticodon triplet, *Sa*IleRS1 recognises the G34 base via backbone and stacking interactions, whereas yeast IleRS2 uses specific side-chain recognition (Supplementary Fig. 12), which is likely preserved in *Pm*IleRS2. Contacts with A35 involve analogous residues from ARD1 in both IleRS1 and IleRS2 enzymes, whereas the reading of U36 differs between them. Protein contacts to U36 present in yeast IleRS2 (Supplementary Fig. 12) and likely in *Pm*IleRS2, where they are conserved at the sequence level, are replaced in *Sa*IleRS1 by interactions with U33 of the tRNA^Ile^ itself (Supplementary Fig. 12). Thus, the structural data indicate that IleRS2 exhibits more specific tRNA^Ile^ anticodon recognition than IleRS1.

**Fig. 3:**
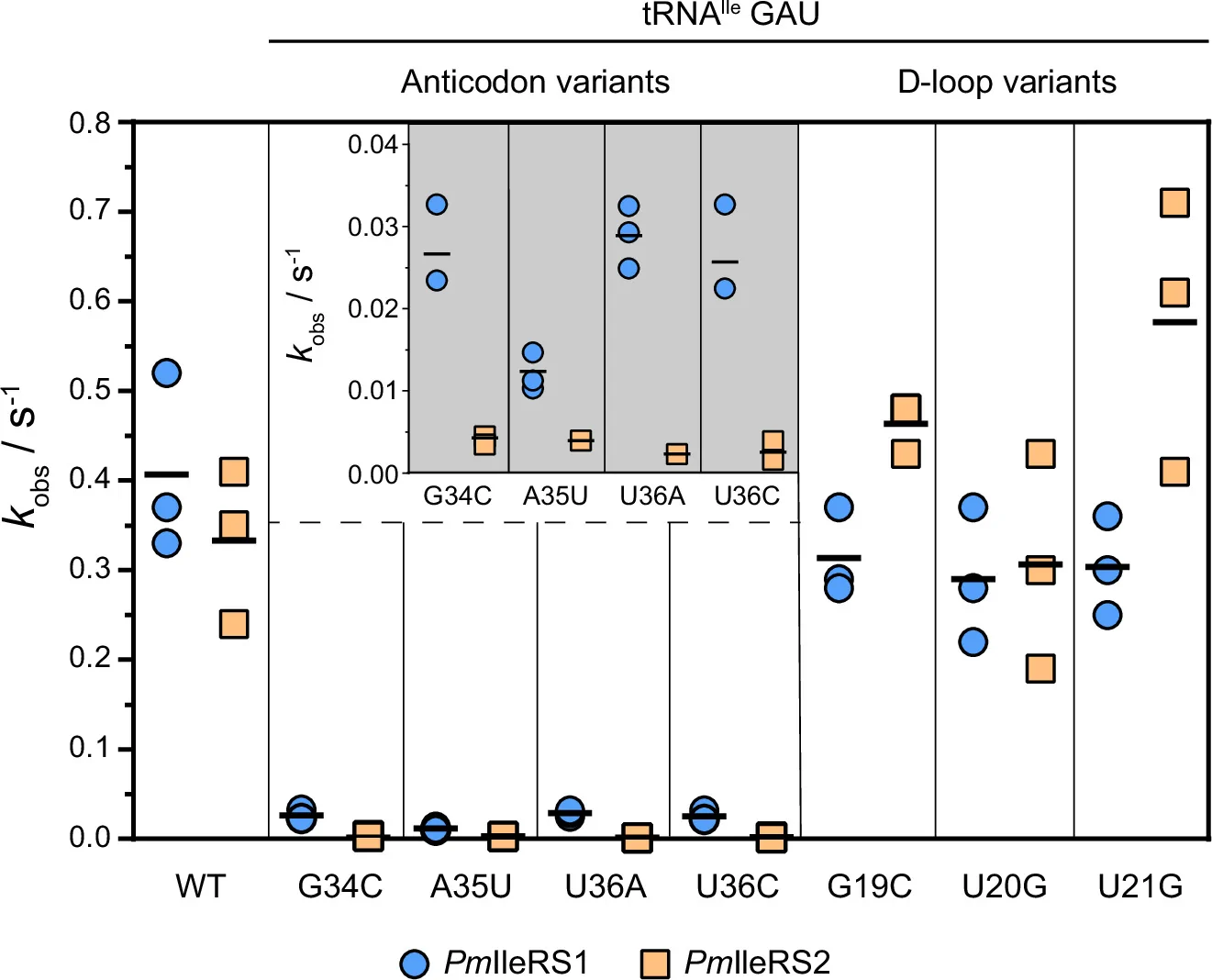
Comparison of the anticodon and D-loop variants of *Pm*tRNA^Ile^ GAU in aminoacylation by *Pm*IleRS1 (blue circles) and *Pm*IleRS2 (orange rectangles). The *k*_obs_ values were obtained at a saturating concentration of tRNA^Ile^ (50 μM), thus providing a close approximation of *k*_cat_ with one exception: for *Pm*IleRS2 with tRNA^Ile^ U20G as a substrate, the *k*_cat_ from detailed steady-state kinetics was used (Table 1). The inset shows an enlarged view of the tRNA^Ile^ anticodon variants. Mean values of three (*n* = 3) independent experiments are indicated with a dash; the standard error of the mean is given in Supplementary Table 2 (for tRNA^Ile^ U20G/IleRS2 in Table 1). Source data are provided as a Source Data file.

To explore this hypothesis through kinetics, we produced anticodon variants of *P. megaterium* tRNA^Ile^ GAU and tested whether they could be aminoacylated by both IleRS1 and IleRS2 from *P. megaterium* (Fig. 3, Supplementary Table 2). As expected, the tRNA^Ile^ variants exhibit a significant reduction in steady-state aminoacylation by *Pm*IleRS1 (also shown for *E. coli* IleRS1^30^) as well as *Pm*IleRS2, demonstrating the key role of the anticodon in tRNA^Ile^ recognition by both enzymes. Interestingly, the tRNA^Ile^ variants were aminoacylated 4-21-fold less efficiently by *Pm*IleRS2 (Fig. 3 inset), indicating that IleRS2 exhibits more stringent anticodon recognition, in line with the structural data. The smallest difference between the two IleRSs was observed for the A35U variant, consistent with analogous reading, whereas G34C, U36A, and U36C variants show larger differences of similar magnitude.

### Anticodon recognition is critical for the isoleucyl transfer step

Although the R721A and R964A IleRS2 mutants cannot aminoacylate tRNA^Ile^ (Table 1), they can participate in the first step of Ile-tRNA^Ile^ formation, Ile activation (Fig. 1A), with *k*_cat_/*K*_M_ values comparable to those of the WT enzyme (Supplementary Table 3). This demonstrates that the mutations influence only the second step, aminoacyl transfer (Fig. 1A). The transfer of the aminoacyl moiety to tRNA^Ile^ could be assessed independently of the activation step by mixing the preformed IleRS:Ile-AMP complex with a limiting amount of tRNA^Ile^ under single-turnover conditions. The IleRS:Ile-AMP complex was formed in situ by incubating IleRS with Ile and ATP before mixing with the tRNA^Ile^.

**Fig. 4:**
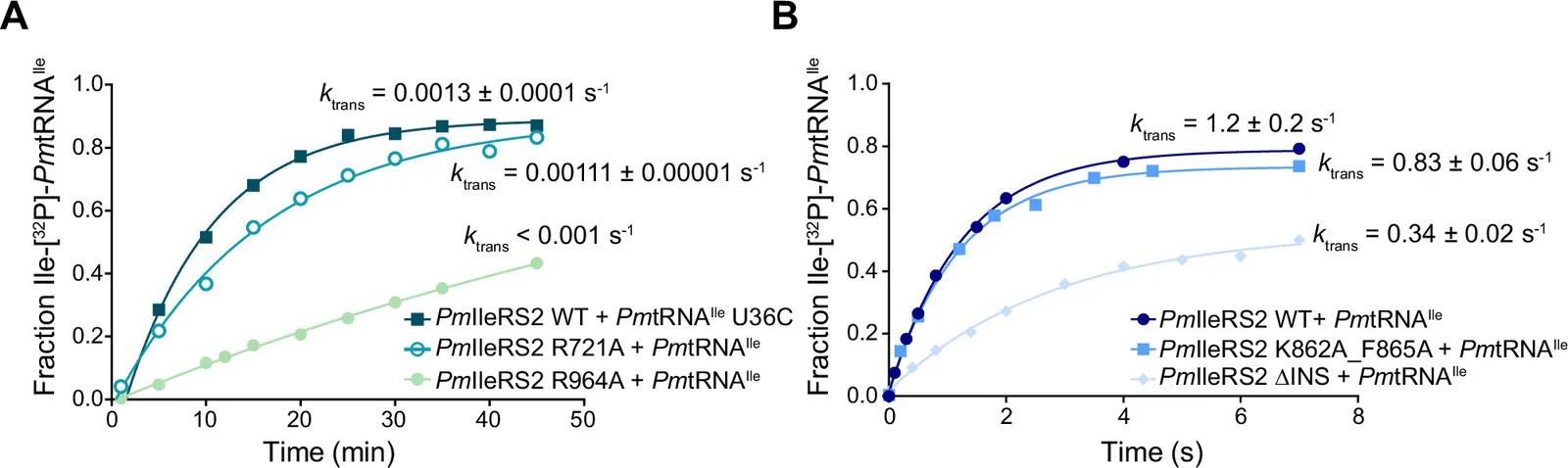
Single-turnover analysis of aminoacyl transfer step by mutants of *Pm*IleRS2 and tRNA^Ile^. **A** Mutation of the anticodon base U36 and the anticodon-reading residues R721 and R964 gave a 10^3^-fold slower transfer step **B** Variations in INS yield up to a 3.5-fold decrease in the transfer step. Doubling the concentration of tRNA^Ile^ returned the same rate, showing that chemistry, not binding, is followed (Supplementary Fig. 13). Time points on a minute time scale (**A**) were obtained by manual mixing, while the ones on millisecond and second time scales (**B**) were sampled using a rapid chemical quench (RQF) instrument. For each protein or tRNA^Ile^, data from the three (*n* = 3) independent experiments were fitted individually (Supplementary Fig. 13). The resulting *k*_trans_ values were used to calculate the mean ± SEM, which are shown in this figure together with representative curves. Source data are provided as a Source Data file.

Single-turnover analysis revealed that R964A and R721A mutants both exhibit a 10^3^-fold drop in isoleucyl transfer rate relative to the WT enzyme (Fig. 4A and Supplementary Fig. 13). Mutating the tRNA^Ile^ instead of the enzyme returned the same outcome: the WT enzyme exhibited a 10^3^-fold lower rate of aminoacyl transfer to a tRNA^Ile^ U36C anticodon variant than to WT tRNA^Ile^ (Fig. 4A and Supplementary Fig. 13). Thus, anticodon recognition is critical for assembling a catalytically competent conformation for the transfer step, consistent with earlier findings that tRNA identity elements preferentially stabilise the transition state for the transfer step^31,32^.

### The INS subdomain mediates non-specific contacts with the tRNA^Ile^ D-loop

To explore interactions of D-loop nucleotides U20 and U21 with the INS subdomain of *Pm*IleRS2 (Fig. 2B), we characterised several U20/U21 tRNA^Ile^ variants, including U20G, U20C, U21G, and U21C. We also characterised the G19C variant, as the D-loop conformation might be disturbed by the absence of the G19:C56 Watson-Crick base pair. The tRNA^Ile^ variants showed no decrease in the aminoacylation rate constants relative to the WT tRNA^Ile^ (Fig. 3 and Supplementary Table 2), indicating that the D-loop establishes base-nonspecific binding interactions with the INS subdomain, known as an indirect readout^33^. Interestingly, the D-loop variants generally showed a slight increase in the rate of aminoacylation by *Pm*IleRS2 relative to WT tRNA^Ile^ (Fig. 3 and Supplementary Table 2). A detailed steady-state analysis using tRNA^Ile^ U20G revealed increases in both *k*_cat_ and *K*_M_ (Table 1). An increase in *k*_cat_ is unexpected for tRNA^Ile^ variants, suggesting that the D-loop does not function as a tRNA^Ile^ identity element and, unlike the anticodon, is not essential for productive juxtaposition of catalytic moieties in the transition state of the aminoacyl transfer step (see below).

The structural data indicate that tRNA^Ile^ recognition by *Pm*IleRS2 (Fig. 2A) differs from *Sa*IleRS1^16^: *Pm*IleRS2 uses INS to interact with the D-loop, while *Sa*IleRS1, lacking the INS subdomain, engages the D-stem of the tRNA^Ile^ via ARD1 (Supplementary Fig. 7 and Supplementary Fig. 14). That said, no change in turnover of the D-loop variants was observed when they were charged by *Pm*IleRS1 (Fig. 3 and Supplementary Table 2). Thus, the small but consistent increase in *k*_cat_ by *Pm*IleRS2 is not related to a change in tRNA^Ile^ structure but is specific to tRNA^Ile^ recognition by IleRS2.

### INS is a tRNA-binding element but not a recognition determinant in *Pm*IleRS2

The finding that the D-loop variant shows an increased *k*_cat_ (Table 1) prompted us to explore the effect of amino acid substitutions in the INS subdomain of *Pm*IleRS2, which should affect binding analogously. To this end, we characterised the aminoacylation activities of the K862A and F865A single mutants and the K862A_F865A double mutant. All mutants exhibit a ~ 4-fold increase in *k*_cat,_ accompanied by a 10-fold rise in *K*_M_ (Table 1), mirroring the effect observed with the tRNA^Ile^ U20G variant (Table 1). Thus, loss of D-loop interactions increases *k*_cat_, suggesting that these interactions restrain a rate-limiting step in the *Pm*IleRS2 catalytic cycle. Earlier findings demonstrated that Ile-tRNA^Ile^ dissociation limits IleRS catalytic turnover^34^, indicating that impairment of the D-loop interactions may enhance Ile-tRNA^Ile^ dissociation.

To test the requirement for an intact INS and to attempt to experimentally realise the ongoing INS loss process inferred from the phylogenetic tree (Fig. 1D), we produced a mutant in which the entire INS subdomain (residues 851-923) was replaced with a short (GGGGS)_2_ linker (ΔINS). The ΔINS protein variant did not exhibit a noticeable difference in its overall fold relative to WT, as assessed by circular dichroism (Supplementary Fig. 15), suggesting that INS makes only a modest contribution to maintaining the IleRS2/IleRS-A architecture. This result is consistent with ΔINS exhibiting only a small reduction in *k*_cat_ and no *K*_M_ effect for Ile in the activation step (Supplementary Table 3). This small reduction in *k*_cat_ is likely due to a small conformational perturbation in ARD2 induced by deletion of the entire INS subdomain. In contrast, truncation increases the *K*_M_ for tRNA^Ile^ in aminoacylation by 15-fold (Table 1), consistent with INS participating in D-loop binding (Fig. 2).

The second step of aminoacylation (the isoleucyl transfer step) can be impaired when recognition of the tRNA identity elements is disrupted^31^. Yet, both the K862A_F865A and ΔINS mutants display only a 1.4- to 3.5-fold decrease in the single-turnover rate constants relative to the WT enzyme (Fig. 4B). This confirms that, in contrast to anticodon recognition (Fig. 4A), *Pm*IleRS2 interactions with the D-loop are not essential to form the catalytically competent conformation for aminoacyl transfer. Instead, the INS subdomain primarily enhances tRNA^Ile^ affinity.

### INS is not mandatory for the functioning of *Pm*IleRS2 in vivo

**Fig. 5:**
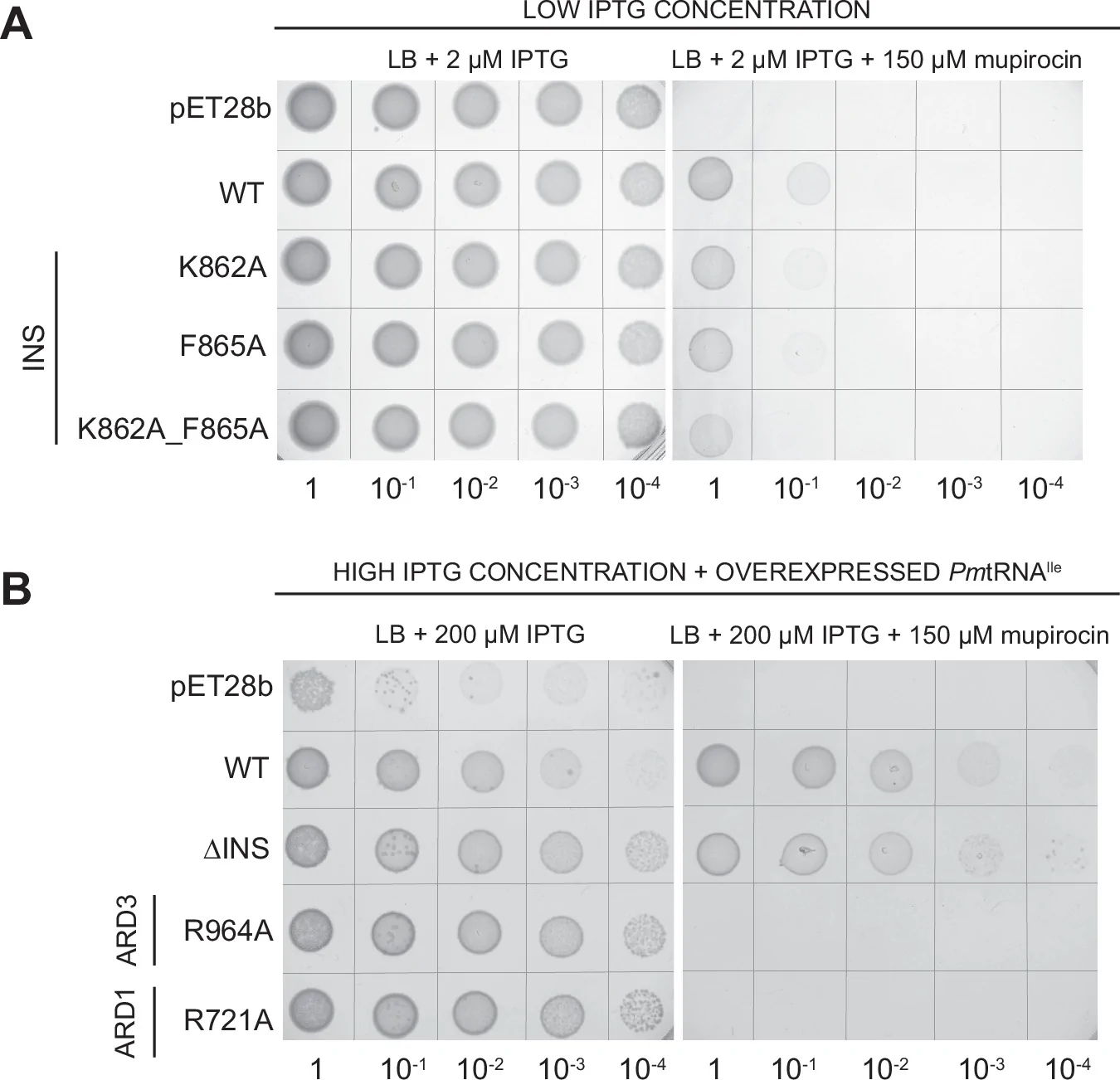
Complementation of *E. coli* growth by *Pm*IleRS2 variants in the presence of mupirocin. Functionality of the *Pm*IleRS2 mutants in vivo was assessed by their ability to complement mupirocin-inhibited *E. coli* IleRS1. Expression of the mutants was induced from the pET28b plasmid using various concentrations of isopropyl β-D-1-thiogalactopyranoside (IPTG) with or without co-overexpression of *Pm*tRNA^Ile^ (Supplementary Fig. 16). **A**,**B** INS mutants rescue *E. coli* growth already at 2 μM IPTG (**A**), while ΔINS requires 200 μM IPTG and co-overexpression of tRNA^Ile^ (**B**). *Pm*IleRS2 anticodon mutants are unable to complement *E. coli* IleRS1, even at 200 μM IPTG and with co-overexpression of tRNA^Ile^ (**B**). For each panel, growth without mupirocin is shown on the left, the negative control (empty plasmid with and without overexpressed tRNA^Ile^) is positioned at the top, and the positive control (overexpression of the WT) is placed in the second row. The experiments were replicated at least twice.

To explore whether INS is required in vivo, we assessed the capacity of INS mutants to rescue *E. coli* growth in the presence of mupirocin. Because mupirocin inhibits the sole endogenous IleRS1 of *E. coli*, growth is dependent on heterologously expressed *Pm*IleRS2 mutants. The K862A, F865A, and K862A_F865A mutants complement *E. coli* growth to a similar extent as WT *Pm*IleRS2 (Supplementary Fig. 16A), even at low IPTG concentration, such as 2 μM (Fig. 5A). Complementation by the ΔINS mutant was more challenging (Supplementary Fig. 16B) and requires a 100-fold higher IPTG concentration as well as co-overexpression of *Pm*tRNA^Ile^ in the cell (Fig. 5B). This coincides with the in vitro data showing that ΔINS exhibits lower *k*_cat_/*K*_M_ compared to other INS mutants due to the lack of a positive effect on *k*_cat_. Thus, our data provide evidence that interactions between INS and the tRNA^Ile^ D-loop are non-essential under the tested conditions, and that even the entire INS subdomain is not mandatory for *Pm*IleRS2 functioning in vivo when expressed at sufficiently high levels.

In sharp contrast, ARD1-based R721A and ARD3-based R964A mutants could not complement the activity of mupirocin-inhibited *E. coli* IleRS1, even at high IPTG induction (Supplementary Fig. 16B) and tRNA^Ile^ co-overexpression (Fig. 5B), in agreement with their abolished anticodon recognition in vitro (Table 1). Finally, the remaining tested IleRS2 mutants complement *E. coli* (Supplementary Fig. 16C) in line with their in vitro aminoacylation efficiencies (Table 1).

## Discussion

### IleRS split into three clades based on the HUP domain and C-terminus

IleRSs have traditionally been subdivided into two groups based on their C-terminal domains, most notably, the Zn-binding domain (ZBD) that is exclusive to IleRS1 (Fig. 1B). IleRS1 (historically referred to as bacterial IleRS) is found in bacteria and eukaryotic organelles, whereas IleRS2 (historically referred to as archaeal/eukaryotic IleRS) resides in archaea, the cytoplasm of eukaryotes, and bacteria, though initial reports suggested modest bacterial occurrence^19^. Later studies revealed that a substantial number of bacteria encode IleRS2^21^. Although the subdivision into two groups was thought to reflect phylogenetic clades, we observed that some archaeal IleRS, classified as IleRS2 by their C-terminal domain, are more closely related to IleRS1^21,22^. This result indicated that the C-terminal domains alone may not accurately reflect IleRS phylogeny.

To address the issue more thoroughly, we conducted a comprehensive phylogenetic analysis and inferred trees based on the different structural modules of IleRS (Supplementary Fig. 3). The HUP domain-based tree, along with other trees, identified three distinct IleRS clades (Fig. 1C, D). Two of the clades align with the traditional classification: i) IleRS1 enzymes present in bacteria and eukaryotic organelles, and ii) IleRS2 enzymes found in archaea, bacteria, and eukaryotic cytoplasm. However, a third clade, designated here as IleRS-A, comprises archaeal enzymes that possess a type 2 C-terminal domain (*i.e*., lacking a ZBD) but have a HUP domain that groups somewhat more closely with that of IleRS1 (referred to as HUP-1). The defining features of HUP-1 are near-absolute conservation of the catalytic HIGH motif, inability to tolerate the natural non-canonical GIHH motif, and sensitivity to the antibiotic mupirocin. Biochemical characterisation demonstrates that IleRS-A enzymes resemble those of IleRS1 (Fig. 1 and Supplementary Fig. 4), supporting their joint classification as HUP-1 enzymes. In contrast, HUP-2, assigned to the IleRS2 enzymes, exhibits mupirocin resistance, which can be further augmented with a GIHH motif^22^. Our data suggest that the split between HUP-1 and HUP-2 predates the emergence of mupirocin, which seems to originate in *Pseudomonas*^23^, with the HUP-2 (IleRS2) lineage likely being naturally predisposed to develop resistance.

Our analysis affords an updated view of IleRS phylogeny (Supplementary Fig. 5). The deep-rooted position of IleRS-A suggests that the HUP-1 domain more closely resembles an ancestral IleRS synthetic domain. The type 2 C-terminal domain architecture likely evolved first, presumably through duplication of an ancestral ARD domain and subsequent insertion of the INS subdomain into ARD2. An early archaeal split gave rise to the IleRS-A and IleRS2 lineages, followed by the subsequent emergence of IleRS1. The observation that all IleRS1 enzymes lack the INS subdomain indicates that the ancestor of IleRS1 had an ARD2 domain without an INS subdomain. Finally, we note that the emergence of IleRS1 likely coincided with the acquisition of ZBD and the loss of ARD3.

### The C-terminal domain reorganisation in IleRS1 was likely driven by demands for faster aminoacylation

Differentiating between the AUA codon for Ile and the AUG codon for methionine (Met) is challenging, because the uridine at the wobble (34) position of the tRNA^Ile^ cannot discriminate between A and G under standard wobble pairing rules^35^. To uncouple decoding of AUA from AUG, different evolutionary lineages employ distinct post-transcriptional modifications at position 34 of tRNA^Ile^^36–38^. Aside from rare cases where tRNA^Ile^ UAU is present, and the ribosome resolves AUA versus AUG codon ambiguity on its own^39^, bacteria encode two tRNA^Ile^ isoacceptors. The major isoacceptor, tRNA^Ile^ GAU, decodes AUU and AUC, while the minor, tRNA^Ile^ CAU, would pair with AUG if left unmodified. Accurate Ile incorporation is ensured by two mechanisms. First and foremost, C34 of the tRNA^Ile^ CAU isoacceptor is post-transcriptionally modified to lysidine (L), which redirects its pairing specificity to the AUA codon and acts as a strong positive determinant for recognition by IleRS. Second, the unmodified tRNA^Ile^ CAU is not recognised by IleRS but is instead charged with Met by MetRS^40^. Because this mischarged Met-tRNA^Ile^ CAU reads the AUG codon, it does not compromise translational fidelity.

Recognition of the wobble position during aminoacylation is mediated by the C-terminal domain of IleRS, which differs between IleRS1 and IleRS2/IleRS-A. It has been proposed^17^ that the ZBD domain was acquired to recognise a bacteria-specific lysidine modification^41,42^. However, the authors overlooked that nearly half of the bacteria harbour IleRS2 instead of IleRS1^21^ (Fig. 1B), and utilise ARD3 for tRNA^Ile^ recognition, suggesting that ZBD in bacteria must serve a different purpose.

What other factors might have driven the acquisition of the ZBD in IleRS1? AARSs have been recognised for important moonlighting roles outside translation^43,44^. Although we cannot rule out the possibility that ZBD contributes to non-canonical AARS functions, our data demonstrate its distinct features in canonical aminoacylation. Namely, aminoacylation of the tRNA^Ile^ anticodon variants reveals that *Pm*IleRS1 is less sensitive to base identity than *Pm*IleRS2 (Fig. 3). This difference may reflect a trade-off between accuracy and velocity in IleRS1, a principle well-documented in translation fidelity^45–48^. Could the absence of INS in IleRS1 (Fig. 1B) stem from similar evolutionary pressures? Bacterial IleRS2 mutants with impaired D-loop interactions display an unexpected increase in *k*_cat_ (Table 1), suggesting that the lack of INS in IleRS1 might also signify a shift towards faster product release, which is the rate-limiting step in IleRS turnover^34^. Therefore, the selection of ZBD and loss of INS could represent an adaptation consistent with increased catalytic velocity of IleRS1 in faster-growing bacteria^26^.

### Transition of the INS subdomain from a tRNA binder to a catalytic mediator

The stages of INS emergence could not be resolved unambiguously (Fig. 1D). However, because we found no evidence for an INS-like insertion in ARD3, INS most likely emerged after the ARD ancestor gave rise to ARD2 and ARD3, but prior to the occurrence of the last common ancestor of IleRS. Indeed, many of the deepest-branching IleRS-A genes contain INS, suggesting that the last common ancestor of IleRS likely possessed this insertion. Given that diverse bacteria and archaea now lack the INS subdomain, we conclude that shortening of IleRS through INS loss is an actively ongoing process in prokaryotes. Taken together, these data suggest that INS loss likely reflects a reversion to an IleRS domain organisation that predates the last common IleRS ancestor (Supplementary Fig. 5).

Our experimental data could explain why the INS subdomain is evolutionarily unstable in bacteria, but rarely lost in eukaryotes. In *Pm*IleRS2, mutations of INS residues F865 and K862 increase the *K*_M_ for tRNA^Ile^ (Table 1), but only lead to a modest reduction in the rate of the aminoacyl transfer step (Fig. 4B). This indicates that, while INS interactions with the D-loop enhance tRNA^Ile^ binding affinity, they do not facilitate maturation of the catalytically productive active site conformation. Because the D-loop is not an identity element in bacteria and likely also in archaea, some IleRS2 and IleRS-A enzymes may naturally lose INS (Fig. 1B), while in those that retain it, like *Pm*IleRS2, INS is not essential for activity either in vitro (Table 1) or in vivo (Fig. 5B). Note that because the aminoacyl transfer step is not rate-limiting, the INS mutants (F865A and K862A) actually display increased *k*_cat_ (Table 1). Along this line, deletion of the entire subdomain results in an enzyme (ΔINS) with an unchanged aminoacylation turnover number (Table 1), providing a rationale for how INS may undergo dynamic loss in prokaryotes.

In contrast to bacteria, the D-loop in yeast apparently serves as an identity element of tRNA^Ile^. Yeast IleRS2 specifically recognises the U20 base via K915 (Supplementary Fig. 12), and removing this interaction using D-loop variants of the tRNA^Ile^ significantly decreases the aminoacylation rate^17^. In line with this evolutionary shift, INS loss events are notably less common in eukaryotes than in bacteria and archaea (Fig. 1B). Thus, we propose that INS initially emerged as a tRNA-binding module with a minor role in juxtaposing reaction moieties for efficient catalysis. Later, in eukaryotes, INS appears to have evolved to play a significant role in adopting the catalytic conformation required for the aminoacyl transfer step, thereby becoming essential.

## Methods

### Construction of IleRS hidden Markov model profiles

Protein sequences from COG0060 (IleRS) were obtained from the EggNOG database v5.0^49^. Sequences were clustered at 60% sequence identity using CD-HIT v4.8.1^50^ [cd-hit -c 0.6 -n 4]. Cluster representatives were aligned with MAFFT L-INS-i v7.505^51^ [mafft --localpair --maxiterate 1000 --thread -1] and trimmed using TrimAl v1.4.rev15^52^ [trimal -gappyout]. The alignment was visually inspected with JalView v2.11.5.0^53^, and outliers were removed manually. Finally, sequences from previously crystallised IleRS1 (PDB 8C9E) and IleRS2 (PDB 8C8V)^22^ were added to the alignment [mafft --add], for a total of 2212 representative IleRS sequences. A phylogenetic tree was inferred for the representative IleRS sequences using IQ-TREE v2.4.0^54^ ModelFinder Plus (MFP)^55^ and ultra-fast bootstrap (UFBoot)^56^ [iqtree -m MFP -B 1000 -T 1] using a single core^57^. Based on the phylogenetic tree, the sequences were divided into two groups, corresponding to IleRS2 and IleRS-A (1047 sequences) and IleRS1 (488 sequences), and re-aligned. Using previously reported domain boundaries for *Pm*IleRS2 and *Pm*IleRS1^22^, reference IleRS crystal structures (PDB:8C8V^22^, PDB:8C9E^22^, PDB:8WND^17^, and PDB:1QU2^16^), secondary structure propensities, and conservation scores, the alignments were segmented into the canonical IleRS domains: HUP, connective peptide 1 (CP1), CP2, editing domain (ED), and anticodon-recognition domain 1 (ARD1). Alignments for the C-terminal domains of IleRS1 (IleRS1_ARD2, and IleRS1_ZBD) and IleRS2/IleRS-A (IleRS2/IleRS-A_ARD2, IleRS2/IleRS-A_ARD3 and IleRS2/IleRS-A_INS) were also generated. Each domain alignment was used to construct a hidden Markov model (HMM) using the HMMER suite v3.3.2^58^ [hmmbuild]. Sequences from the closely related AARS families ValRS (COG0525), LeuRS (COG0495), and MetRS (COG0143) were obtained from the EggNOG database and clustered as above. IleRS HMM profiles were then analysed by searching [hmmsearch --domtblout] against ValRS, LeuRS, and MetRS to assess the extent of discrimination at the domain level (Supplementary Fig. 1).

### IleRS sequence database construction

The IleRS domain HMM profiles were used to search the Genome Taxonomy Database (GTDB) v220.0^59^ and the EukProt v3^60^ database. Using a cutoff of 0.75 profile coverage and 0.01 iE-value, domain annotations were assigned, with preference given to non-overlapping hits with lower iE-values. The domain annotation script, DOTATE v1.2.0, is deposited in the Zenodo repository under DOI: 10.5281/zenodo.21453230^61^. Selection criteria for candidate IleRS sequences were (a) matching at least four of the five canonical IleRS core domains (HUP, CP1, ED, CP2, and ARD1) and (b) matching at least one IleRS type-specific domain (IleRS1_ARD2, IleRS2/IleRS-A_ARD2, IleRS1_ZBD, IleRS2/IleRS-A_ARD3, IleRS2/IleRS-A_INS). In total, 7,360 archaeal, 109,435 bacterial, and 1,588 eukaryotic proteins were classified as potential IleRSs.

Once again, COG IleRS, ValRS, LeuRS, and MetRS reference sequences were clustered as above and added to a custom sequence database using BLAST v2.17.0^62^ [makeblastdb]. Candidate IleRS sequences were clustered at 70%, 60%, and 50% sequence identity for eukaryotic, archaeal, and bacterial genes, respectively, using CD-HIT as above. Representative genes were searched against the labelled COG sequences [blastp -outfmt “6 qseqid sseqid pident length evalue bitscore qlen slen” -evalue 1e-5], and a sequence similarity network (SSN) was constructed by filtering hits with pident <30 and coverage <0.5. Network visualisations were performed in Cytoscape v3.10.4^63^ using the perfuse force-directed layout weighted by bit-score. Only sequences whose representative clustered with a COG IleRS were retained (Supplementary Fig. 1), resulting in a total 97,678 IleRS sequences.

Filtered IleRS sequences were classified according to their C-terminal domain configurations and compiled into a validated IleRS database integrating data from Bacteria, Archaea and Eukarya. From this database, domain composition analyses were calculated.

### Phylogenetic analysis

Representative sequences from Archaea and Bacteria in the IleRS database were randomly selected at the family level according to the GTDB taxonomy. Bacterial representatives were clustered at 60% sequence identity as above. The final dataset included all eukaryotic IleRS sequences, family-level representatives for Archaea, and family-level clustered representatives for Bacteria, providing an approximately balanced representation across the three domains of life. ValRS sequences from COG were clustered and aligned as above, and the homologous domains for HUP, CP1, ED, CP2, and ARD1 were manually extracted. 50 ValRS sequences, composed of 25 bacterial and 25 archaeal representatives, were included as outgroups for tree rooting. Multiple sequence alignments were generated for IleRS domain sequences, then merged using MAFFT–merge [mafft --–localpair--–maxiterate 1000 --merge] with the corresponding ValRS alignment. Then the merged alignment was trimmed as above using TrimAl -automated1 option (optimised for Maximum Likelihood phylogenetic tree calculation). Phylogenies were then inferred using IQ-TREE as above. To ensure the robustness of the phylogenetic inferences, the representative sampling procedure was repeated for each new domain to be analysed (*i.e*., family representatives were re-randomised, re-clustered, re-aligned, and new trees were calculated). The phylogeny for each domain was calculated five times, with MFP only applied in the first run. The resulting model for that domain was reused across the other four runs (HUP: LG + R10, CP1: LG + R9, ED: LG + R9, CP2: LG + R9, ARD1: LG + G4). Consistency of topological features across these replicates, as well as across domains, was used as a measure of robustness. For each domain, all five consensus trees were topologically consistent, and the trees with the highest log-likelihood are presented in Supplementary Fig. 3.

### Preparation and purification of *E. coli* tRNA^Ile^

*E. coli* tRNA^Ile^ GAU (*Ec*tRNA^Ile^ GAU) was transcribed in *E. coli* BL21(DE3) for 15 h at 30 °C using 1 mM isopropyl-β-D-1-thiogalactopyranoside (IPTG) from a ΔpET3a-derived plasmid containing the tRNA gene. The first base pair of the *Ec*tRNA^Ile^ GAU was changed from A–U to G–C to improve transcription yield, without affecting kinetic competence^12^. Luria-Bertani (LB) medium was supplemented with 5 mM MgCl₂ to improve the yield. The crude tRNA was purified through sequential phenol:chloroform extraction, polyethylene glycol precipitation, deacylation, and dialysis, according to established protocols^64^. Isolation of high-homogeneity tRNA^Ile^ GAU from the crude tRNA preparation was conducted by reversed-phase chromatography on a Jupiter® C4 300 Å, 5 µm, 250 × 4.6 mm column (Phenomenex)^10,11^. Fractions with 95% or higher tRNA^Ile^ purity were pooled, dialysed against 5 mM HEPES-KOH (pH 7.5 at 20 °C), lyophilised, and stored at −80 °C. The final preparation for crystallisation had an isoacceptor content exceeding 98% as determined by aminoacylation.

### Crystallisation

Wild-type *Pm*IleRS2 was diluted to 19.77 mg mL^−1^ in 25 mM HEPES-KOH (pH 7.5, 20 °C), 50 mM NaCl, and 10 mM β-mercaptoethanol. *Ec*tRNA^Ile^ GAU stock (99.34 mg mL^−1^ in 5 mM HEPES-KOH, pH 7.5, 20 °C) was heat-renatured by dilution with MgCl₂ to 55.1 mg mL^−1^ tRNA^Ile^ and 100 mM MgCl₂. The ternary complex was assembled by mixing 38.5 µL *Pm*IleRS2, 6.5 µL renatured tRNA^Ile^, and 5 µL 100 mM ATP (in protein buffer), yielding a ~ 1:2 protein:tRNA molar ratio with 10 mM ATP. Crystals were grown at 20 °C by sitting-drop vapour diffusion, using 1 + 1 µl drops of complex and reservoir solutions (50 mM HEPES-NaOH, pH 6–8, 50 mM MgCl_2_, 7.5–15% PEG3350, 0.1 M NH_4_OAc). After 2–7 days, the crystals were stabilised by adding 25% pentaerythritol propoxylate (5/4 PO/OH) in one step. After incubation for 5 minutes, the crystals were mounted on Hampton loops and flash-frozen in liquid nitrogen.

### Data collection and processing

Diffraction data were collected at beamline X06SA (Swiss Light Source, Paul Scherrer Institut, Villigen, Switzerland) using a Dectris Eiger 16 M detector and a wavelength of 1.0 Å. A total of 3600 frames (0.1° oscillation angle) were indexed and scaled with XDS BUILD = 20220220 and XSCALE BUILD = 20220220^65^ into space group P2_1_2_1_2_1_ (unit cell: 121.75 × 147.01 × 344.11 Å; α = β = γ = 90°), yielding usable data to 5.3 Å resolution based on CC₁_/_₂ criterion^66^ (Supplementary Table 1).

### Phasing, model building and coordinate refinement

An initial molecular replacement solution was obtained using PHASER v2.8.3^67^ with a search model assembled from the *Pm*IleRS2:Ile-AMS structure (PDB: 8C8V)^22^ and including the tRNA^Ile^ from the superimposed *Sa*IleRS1:tRNA^Ile^:mupirocin structure (PDB: 1FFY)^16^, revealing four copies of the complex in the asymmetric unit (ASU) with a solvent content of ~59% (Supplementary Figs. 10A, 11). Clear m*F*_o_–D*F*_c_ Fourier difference density was observed for omitted tRNA and protein parts after rigid body refinement in PHENIX v1.21.2_5419^68^ using the individual *Pm*IleRS2 domains and tRNAs as groups (Supplementary Fig. 6A). The quality of the map was improved by four-fold real-space averaging in CCP4 v7.1.018^69^, which allowed completion of the tRNA model (Supplementary Fig. 6B). The less-well-resolved domains of the protein were repositioned manually by rigid body docking into unbiased OMIT maps and rebuilding of the connections (exemplified for the INS subdomain in Supplementary Fig. 7). The model was further improved by adjusting peripheral loops according to m*F*_o_–D*F*_c_ difference Fourier maps. The improvement of the phases was monitored by calculation of anomalous difference Fourier maps for the zinc ions in the zinc fingers (Supplementary Fig. 6C, D), which resulted in increasing peak σ levels with decreasing *R*_work_ and *R*_free_ values, and by calculation of simulated annealing composite OMIT maps to minimise model bias. Because only weak residual m*F*_o_–D*F*_c_ Fourier difference density was observed for the bound ATP cofactors, the ligand was not modelled. Care was taken to keep the model geometry intact and to prevent over-refinement by only editing the original model and refining it to convergence. This approach allowed progressive improvement of the model-to-map fit with a concomitant decrease in *R*_free_ and *R*_work_ values. To remove side chain clashes between neighbouring copies in the ASU, the completed input model was first subjected to *phenix.geometry_minimization* for 3 macrocycles (each comprising 100 iterations), followed by several cycles of coordinate refinement to convergence with *phenix.refine*. In order to prevent overfitting, secondary structure restraints were applied for both the protein (Ramachandran, secondary structure, torsion-angle NCS) and the tRNA (base pair, stacking pair, torsion-angle NCS). Further, B-factors were refined using the group-wise setting (two groups per residue), and a fixed value of *wxc* = 0.6 for the weighting between the geometry and X-ray targets was chosen. To take into account the anisotropic motion of domains, the B-factor refinement also included domain-wise TLS (translation-rotation-screw-rotation) (Supplementary Fig. 11), which further improved the *R*_work_ and *R*_free_ values by ~ 1% to 20.5% and 25%, respectively (Supplementary Table 1). The final model, validated using MolProbity v4.5.2^70^, exhibited excellent statistics for the resolution (Supplementary Table 1).

Comparison of the final model with the recently published 2.8 Å crystal structure of the yeast IleRS2:tRNA^Ile^:isoleucine complex (PDB: 8WND)^17^ revealed only minor differences for the protein, essentially confirming our low-resolution model. However, as in the yeast complex, some base orientations in the anticodon loop and the acceptor stem of the tRNA^Ile^ described the electron density more accurately; they were consequently readjusted in the tRNA^Ile^ bound to *Pm*IleRS2, and the structure was re-refined. The deposited final model (PDB accession code 9U0O, corresponding to extended pdb_00009U0O) includes all side chains and is meant to represent a pseudoatomic interpretation of the *Pm*IleRS2:*Ec*tRNA^Ile^:ATP complex.

### Initial structural comparison of the four *Pm*IleRS2:tRNA^Ile^ complexes in the asymmetric unit

Superposition of all four molecules in the ASU (Supplementary Fig. 10A–D) revealed modest changes in the relative orientations between domains and tRNAs, suggesting that these differences result from crystal packing interactions rather than representing functionally distinct states. This interpretation is further supported by superposition of the yeast structure (PDB 8WND), where the interdomain motions are within a similar range (Supplementary Fig. 10E). For the *Pm*IleRS2 complexes, the highest intrinsic domain flexibility is observed for the INS subdomain, which is also reflected by the B-factor distribution (Supplementary Fig. 11). As a result of this structural analysis, only the best-ordered complex (comprising chains A for protein and a for tRNA, respectively) was used for figure generation throughout the manuscript.

### Cloning and mutagenesis

The DNA sequence encoding *Pm*IleRS2 was cloned into the pET28b(+) plasmid (Novagen), and *Pm*tRNA^Ile^ GAU was cloned into pET3a (Novagen), in which 191 base pairs between two *Eco*RV restriction sites were deleted (ΔpET3a). Expression vectors for *Pm*IleRS2 and *Pm*tRNA^Ile^ GAU mutants were generated by whole-plasmid amplification of pET28b(+)_*Pm*IleRS2 or ΔpET3a_*Pm*tRNA^Ile^ GAU using Phusion High-Fidelity DNA Polymerase (Thermo Scientific) according to the manufacturer’s recommendations. Mutations were introduced by site-directed mutagenesis using custom-designed primers (Supplementary Table 4). All mutants were verified by sequencing (Macrogen Inc.). Archaeal proteins (MaIleRS-A (GCA_016177165.1_JACOVT010000077.1_5), WaIleRS-A (GCA_016219835.1_ JACRKL010000046.1_19) and DaIleRS2 (GCA_017608775.1_JAFCFF010000015.1_11)) as well as their GIHH mutants were obtained as synthetic expression plasmids ordered from Twist Bioscience.

### Production and purification of proteins

Wild-type *Pm*IleRS2 and its mutants were overexpressed in *E. coli* BL21(DE3) cells in LB medium supplemented with 1 mM MgCl₂ at 37 °C, with 1 mM ZnCl₂ added to the culture at an OD_600_ of approximately 0.1. Protein expression was induced with 0.25 mM IPTG and incubated at 15 °C overnight^22^. The cells were harvested by centrifugation and resuspended in 10 or 25 mL of washing buffer (20 mM HEPES-KOH, pH 7.5, 500 mM NaCl, 20 mM MgCl_2_, 10 mM imidazole, 10 mM 2-mercaptoethanol), depending on the method used for cell lysis. Two methods were employed for cell lysis: sonication^22^ and high-pressure homogenisation (Constant Systems Cell Disruptor BT40/T52/AA) at 20,000 psi (1378 bar). The soluble protein fraction was loaded onto a HisTrap HP column (Cytiva, 1 or 5 mL) and the protein was eluted with 200 mM imidazole. A further purification step included size-exclusion chromatography on a Superdex 200 Increase 10/300 GL (Cytiva) or HiLoad Superdex 200 16/600 (Cytiva) column in a buffer containing 20 mM HEPES-KOH (pH 7.5), 50 mM NaCl, 10% glycerol, and 10 mM 2-mercaptoethanol. Fractions with monomeric protein were pooled, concentrated to ~20 mg mL^−1^ and stored at −80 °C. Archaeal IleRS variants were overexpressed and purified using the same workflow, except for DaIleRS2 and its GIHH mutant, which were overexpressed for 4 h at 30 °C. For these two proteins, the lysis and purification buffers were adjusted to pH 8.2. All tested proteins were generally expressed from two or three distinct clones, purified and tested independently in each kinetic assay.

### In vitro transcription and purification of tRNA

The DNA template required for in vitro transcription was amplified by PCR using the plasmid ΔpET3a_*Pm*tRNA^Ile^ GAU, Phusion DNA polymerase, an upstream primer: 5’-TCTCAAGGGCATCGGTCGAC-3’, and a specific 2’-*O*-methylated downstream primer: 5’-TmGGTGGGCCTAAGTGGACTC-3’. In vitro transcription was performed at 37 °C for 4 h in a 1 mL reaction mixture containing 1.8 μg of DNA template, 40 mM Tris-HCl (pH 8.0), 20 mM MgCl_2_, 2 mM spermidine (Sigma-Aldrich), 10 mM dithiothreitol (DTT), 0.005 mg mL^−1^ bovine serum albumin (BSA) (NEB), 5 mM NTPs, 0.008 U μL^−1^ Thermostable Inorganic Pyrophosphatase (TIPP) (NEB), 0.05 U μL^−1^ RNase inhibitor (NEB), and 0.1 mg mL^−1^ T7 RNA polymerase. The resulting tRNA^Ile^ transcript was purified by preparative denaturing gel electrophoresis on a 6% (w/v) polyacrylamide gel. After electrophoresis, tRNA^Ile^ was extracted from the gel using a buffer containing 50 mM Tris-HCl (pH 7.5), 10 mM MgCl_2_, 1 mM EDTA, and 0.1% SDS. The transcript was then precipitated with 96% ice-cold ethanol and 3 M NaOAc (pH 5.2), followed by centrifugation and washing with 70% ice-cold ethanol. Finally, tRNA^Ile^ was dialysed against 10 mM HEPES-KOH (pH 7.5) and kept at −20 °C. Labelled [^32^P]-tRNAs were prepared by enzymatic exchange of terminal adenosine with [^32^P]AMP using tRNA nucleotidyltransferase, TIPP (NEB) and α-[^32^P]ATP (Perkin Elmer). The labelled tRNA was purified by phenol/chloroform extraction and two consecutive Bio-Spin P-30 (Bio-Rad) chromatography steps to remove unincorporated α-[^32^P]ATP. Both unlabelled and radiolabelled tRNA^Ile^ were dialysed against 10 mM HEPES-KOH (pH 7.5) and stored at −20 °C. Transcripts were renatured before use by heating at 85 °C for 3 min, followed by slow cooling to reaction temperature.

### Amino acid activation assay

Amino acid activation assay was monitored by an ATP-PP_i_ exchange assay using radioactively labelled γ-[^32^P]ATP^71^ (Perkin Elmer). The reactions were carried out at 30 °C (65 °C for WT DaIleRS2 and its GIHH mutant) in a buffer containing 20 mM HEPES-KOH (pH 7.5) (20 mM MES - KOH (pH 6.5) for WT DaIleRS2 and its GIHH mutant), 10 mM MgCl_2_, 0.1 mg mL^−1^ BSA, 5 mM DTT, 4 mM [^32^P]ATP, and 1 mM PP_i_. The *Pm*IleRS2 and its variants were present at 20 or 100 nM, while WaIleRS-A, MaIleRS-A and DaIleRS2 were present at 200 or 500 nM. For *k*_cat_ and *K*_M_ (Ile) determination of *Pm*IleRS2 mutants, Ile was used at concentrations ranging from 0.1 to 10 times the *K*_M_ values. To test the activity of GIHH mutants of archaeal enzymes, Ile was present at a concentration of 10 times the *K*_M_ value for the corresponding archaeal WT enzyme. The inhibition of archaeal IleRS enzymes by mupirocin was assessed using Ile concentrations corresponding to the *K*_M_ (Ile) values. Mupirocin was present at concentrations of 1 µM for DaIleRS2 and WaIleRS-A and 50 µM for MaIleRS-A.

The reactions were stopped at various time points with 0.5 M NaOAc (pH 5.2) and 0.1% SDS, followed by product separation by thin-layer chromatography (TLC) on polyethyleneimine (PEI) plates (Macherey-Nagel) in a buffer containing 750 mM KH_2_PO_4_, pH 3.5 and 4 M urea. Separated signals for [^32^P]ATP and [^32^P]PP_i_ were detected using a Typhoon phosphorimager (GE Healthcare) and quantified using ImageQuant software. The reaction rate was determined from a linear plot of product concentration versus time. The *k*_cat_ and *K*_M_ (Ile) were obtained by fitting the data to the Michaelis–Menten equation using GraphPad Prism 6.

### Aminoacylation assay

Aminoacylation assay was performed at 30 °C in a reaction mixture containing 20 mM HEPES-KOH (pH 7.5), 150 mM NH_4_Cl, 10 mM MgCl_2_, 0.008 U μL^−1^ TIPP, 0.01 mg mL^−1^ BSA, 5 mM DTT, and 2 mM ATP. The enzyme was present at concentrations ranging from 10 to 2000 nM. The steady-state reaction was started by adding Ile (final concentration 1 mM) to mixtures containing [^32^P]tRNA^Ile^ at concentrations ranging from 0.1 to 10 times the *K*_M_. The reaction was stopped at various time points by 0.1 M NaOAc and 5% HOAc, and the quenched aliquots were treated with P1 nuclease^72^ (Sigma-Aldrich), followed by product separation by TLC on PEI plates in a buffer containing 100 mM NaOAc pH 5.0 and 5% HOAc. Separated aa-3’-[^32^P]AMP (representing aa-tRNA) and [^32^P]AMP (representing tRNA) were detected using a Typhoon phosphorimager (GE Healthcare) and quantified using ImageQuant software. The *k*_cat_ and *K*_M_ (tRNA^Ile^) were obtained by fitting the data to the Michaelis–Menten equation using GraphPad Prism 6. For tRNA^Ile^ anticodon and D-loop variants, reactions were carried out at a saturating concentration of tRNA^Ile^ (50 μM) and 40 μM [^14^C]Ile (50–100 mCi mmol^−1^) (Perkin Elmer) under the otherwise same reaction conditions stated above. At the indicated time points, aliquots were spotted on Whatman 3MM filter discs, quenched in ice-cold 10% trichloroacetic acid (TCA), and washed twice with cold 5% TCA. The amount of [^14^C]Ile-tRNA^Ile^ retained on the filters was quantified by liquid scintillation counting. The reaction rate was determined from a linear plot of product concentration versus time.

### Single-turnover transfer of an amino acid to tRNA

The transfer of isoleucine to tRNA^Ile^ was measured by mixing IleRS:aa-AMP complex formed in situ with a limiting concentration of [^32^P]tRNA^Ile^. 20–100 μM enzyme was first incubated for 10 min at 30 °C with 4 mM Ile and 4 mM ATP in a buffer containing 20 mM HEPES-KOH (pH 7.5), 150 mM NH_4_Cl, 10 mM MgCl_2_, 0.008 U μL^−1^ TIPP, 0.01 mg mL^−1^ BSA, and 5 mM DTT to allow for IleRS:aa-AMP complex formation. The transfer reaction was initiated by mixing the solution with an equal volume of 2-10 μM [^32^P]tRNA^Ile^ and stopped at various time points with 0.1 M NaOAc and 5% HOAc. The reactions were tested at a double tRNA^Ile^ concentration to confirm that the chemistry step, rather than binding, is followed. The quenched aliquots were treated with P1 nuclease, followed by product separation by TLC and quantification, as for the aminoacylation assay. The *k*_trans_ was extracted by fitting the experimental data into a single exponential equation $y={Y}_{0}+A\times (1-{e}^{-{k}_{{\rm{trans}}}\times {t}})$ using GraphPad Prism 6.

### Complementation assay

Overnight cultures were prepared from *E. coli* BL21(DE3) cells transformed with a single plasmid expressing the enzyme of interest or co-transformed with both the enzyme-expression plasmid and the *Pm*tRNA^Ile^-expression plasmid. The overnight cultures were diluted to an OD_600_ of 1 for the complementation assay. The cultures were then subjected to five serial dilutions. Aliquots of 10 µL from each dilution were spotted onto LB plates, which were supplemented with 30 µg mL^−1^ kanamycin, 100 µg mL^−1^ ampicillin, 1 mM MgCl_2_, and 0.1 mM ZnCl_2_, with or without the addition of 150 µM mupirocin. IPTG was added to LB agar medium at concentrations ranging from 2 µM to 200 µM. The plates were grown for 24 h at 30 °C.

### Reporting summary

Further information on research design is available in the Nature Portfolio Reporting Summary linked to this article.

## Supplementary information

Reporting Summary

Transparent Peer Review file

Supplementary information

## Source data

Source Data

## Data Availability

Protein sequences of IleRS, ValRS, LeuRS and MetRS were obtained from EggNOG database v5.0 under accession codes COG0060, COG0525, COG0495 and COG0143, respectively. IleRS domain HMM profiles were used to search the Genome Taxonomy Database (GTDB) v220.0 and the EukProt v3 database. The structural data generated in this study have been deposited in the Protein Data Bank (PDB) under accession number: 9U0O (*Pm*IleRS2:*Ec*tRNA^Ile^ GAU:ATP). The previously published structural data used in this study are available in the PDB under accession codes 1FFY, 8C8V, 8WND, 8C9E, and 1QU2. Raw kinetic data generated in this study (Figs. 3 and 4, Table 1, Supplementary Figs. 4 and 13, Supplementary Tables 2 and 3) are provided in the Source data file. Phylogenetic data (Fig. 1 and Supplementary Figs. 1-3) are deposited in the Zenodo repository under https://doi.org/10.5281/zenodo.21453230^61^. Other relevant data are either contained in the manuscript or provided in the Supplementary Information file. Source data are provided with this paper.

## Code availability

The custom code used in this study for mapping protein domains onto sequences (DOTATE, v1.2) is publicly available, archived in a Zenodo repository under a Creative Commons Attribution 4.0 International license, and accessible via https://doi.org/10.5281/zenodo.21453230^61^.
