# Supplementary material for: C-terminal evolutionary remodelling of isoleucyl-tRNA synthetases is a prokaryote-specific strategy for tuning aminoacylation rate
Source: Nat Commun. 2026 Oct 12;17:11575. doi: 10.1038/s41467-026-78241-1 (PMC13752280; doi:10.1038/s41467-026-78241-1)
Supplement: Supplementary file 1 [file 41467_2026_78241_MOESM1_ESM.pdf]

nature portfolio

Corresponding author(s): Ita Gruic-Sovulj, Liam M. Longo, and Nenad Ban

Last updated by author(s): 2026/09/10

Reporting Summary

Nature Portfolio wishes to improve the reproducibility of the work that we publish. This form provides structure for consistency and transparency in reporting. For further information on Nature Portfolio policies, see our Editorial Policies and the Editorial Policy Checklist.

Statistics

For all statistical analyses, confirm that the following items are present in the figure legend, table legend, main text, or Methods section.

n/a	Confirmed	
[ ]	[x]	The exact sample size (*n*) for each experimental group/condition, given as a discrete number and unit of measurement
[ ]	[x]	A statement on whether measurements were taken from distinct samples or whether the same sample was measured repeatedly
[x]	[ ]	The statistical test(s) used AND whether they are one- or two-sided<br>*Only common tests should be described solely by name; describe more complex techniques in the Methods section.*
[x]	[ ]	A description of all covariates tested
[x]	[ ]	A description of any assumptions or corrections, such as tests of normality and adjustment for multiple comparisons
[ ]	[x]	A full description of the statistical parameters including central tendency (e.g. means) or other basic estimates (e.g. regression coefficient) AND variation (e.g. standard deviation) or associated estimates of uncertainty (e.g. confidence intervals)
[x]	[ ]	For null hypothesis testing, the test statistic (e.g. *F*, *t*, *r*) with confidence intervals, effect sizes, degrees of freedom and *P* value noted<br>*Give P values as exact values whenever suitable.*
[x]	[ ]	For Bayesian analysis, information on the choice of priors and Markov chain Monte Carlo settings
[x]	[ ]	For hierarchical and complex designs, identification of the appropriate level for tests and full reporting of outcomes
[x]	[ ]	Estimates of effect sizes (e.g. Cohen's *d*, Pearson's *r*), indicating how they were calculated

Our web collection on statistics for biologists contains articles on many of the points above.

Software and code

Policy information about availability of computer code

Data collection	The X-ray data were collected using beamline X06SA (Swiss Light Source, Paul Scherrer Institut, Villigen, Switzerland) using a Dectris Eiger 16M detector.
Data analysis	Protein sequences were clustered at 60% sequence identity using CD-HIT v4.8.1 [cd-hit -c 0.6 -n 4]. Cluster representatives were aligned with MAFFT L-INS-i v7.505 [mafft --localpair --maxiterate 1000 --thread -1] and trimmed using TrimAl v1.4.rev15 [trimal -gappyout]. The alignment was visually inspected with JalView v2.11.5.0. A phylogenetic tree was inferred for the representative IleRS sequences using IQ-TREE v2.4.0, Model Finder Pro (MFP) and ultra-fast bootstrap (UFBoot) [iqtree -m MFP -B 1000 -T 1] using a single core. Each domain alignment was used to construct a hidden Markov model (HMM) using the HMMER suite v3.3.2 [hmmbuild]. The domain annotation script, DOTATE v1.2.0, is available in Zenodo repository under DOI: 10.5281/zenodo.21453230. Network visualizations were performed in Cytoscape v3.10.4. XDS BUILD=20220220 and XSCALE BUILD=20220220 were used for X-ray diffraction data processing. An initial molecular replacement solution was obtained using PHASER v2.8.3 implemented in CCP4 v7.1.018. The initial rigid body and following full coordinate refinement was performed in PHENIX v1.21.2_5419. The quality of the map was improved by four-fold real-space NCS averaging in CCP4 v7.1.018. The structure was iteratively rebuilt in COOT v0.9.8.92. The final model was validated using MolProbity v4.5.2. Structural figure captions were generated in PyMOL v3.1.8 and assembled in Adobe Illustrator v29.8.10. All data for kinetic characterization of enzymes and tRNA were analyzed using the GraphPad Prism v6.0 software.

For manuscripts utilizing custom algorithms or software that are central to the research but not yet described in published literature, software must be made available to editors and reviewers. We strongly encourage code deposition in a community repository (e.g. GitHub). See the Nature Portfolio guidelines for submitting code & software for further information.

Data

Policy information about availability of data

All manuscripts must include a data availability statement. This statement should provide the following information, where applicable:

- Accession codes, unique identifiers, or web links for publicly available datasets
- A description of any restrictions on data availability
- For clinical datasets or third party data, please ensure that the statement adheres to our policy

Protein sequences of IleRS, ValRS, LeuRS and MetRS were obtained from EggNOG database v5.0 under accession codes COG0060, COG0525, COG0495 and COG0143 respectively. IleRS domain HMM profiles were used to search the Genome Taxonomy Database (GTDB) v220.0 and the EukProt v3 database. The coordinates and structural factors of PmIleRS2:EctRNAIle:ATP complex has been deposited in the Protein Data Bank (PDB) under the accession code 9U0O [https://doi.org/10.2210/pdb9U0O/pdb]. Previously published structural data used in this study are available in the PDB under accession codes 1FFY [https://doi.org/10.2210/pdb1ffy/pdb], 8C8V [https://doi.org/10.2210/pdb8c8v/pdb], 8WND [https://doi.org/10.2210/pdb8wnd/pdb], 8C9E [https://doi.org/10.2210/pdb8c9e/pdb] and 1QU2 [https://doi.org/10.2210/pdb1qu2/pdb]. The source data for results of kinetic characterization are provided as a Source Data file. Phylogenetic data are deposited in a Zenodo repository under DOI: 10.5281/zenodo.21453230.

Research involving human participants, their data, or biological material

Policy information about studies with human participants or human data. See also policy information about sex, gender (identity/presentation), and sexual orientation and race, ethnicity and racism.

Reporting on sex and gender	Information not related to our study.
Reporting on race, ethnicity, or other socially relevant groupings	Information not related to our study.
Population characteristics	Information not related to our study.
Recruitment	Information not related to our study.
Ethics oversight	Information not related to our study.

Note that full information on the approval of the study protocol must also be provided in the manuscript.

Field-specific reporting

Please select the one below that is the best fit for your research. If you are not sure, read the appropriate sections before making your selection.

[x] Life sciences [ ] Behavioural & social sciences [ ] Ecological, evolutionary & environmental sciences

For a reference copy of the document with all sections, see nature.com/documents/nr-reporting-summary-flat.pdf

Life sciences study design

All studies must disclose on these points even when the disclosure is negative.

Sample size	The kinetic assays were conducted in at least three independent experiments, with seven to nine time points collected to determine the observed rate constants in each experiment. 19 wild-type proteins or mutants and 10 tRNA variants were kinetically characterised. The sample size for in vivo studies included 9 proteins under various growth conditions. In each cases, the sample size was determined by commonly used practice in kinetic characterisation and common rationale when studying protein:tRNA interactions. The curated IleRS database constructed in this study consists of 97,678 sequences. Bacterial representatives were clustered at 60% sequence identity to reduce sample size, consistent with common practice in phylogenetic analysis.
Data exclusions	No data were excluded from the analyses.
Replication	The kinetic measurements were repeated in three independent experiments, although for some protein variants and assays, four or five independent experiments were performed. The independent experiments were reliably reproducible. Experiments were performed using an independently prepared enzyme and/or tRNA samples generally expressed from two or three distinct clones, to verify reproducibility. Kinetic parameters were determined independently for each experiment, and the reported values represent the mean ± SEM. The observed trends were consistent across independent experiments. The in vivo experiments were performed in at least two independent experiments, showing a high level of reproducibility. The phylogenetic trees were calculated five times using repeatedly randomised family-level representatives from Archaea and Bacteria. The observed trends were consistent across the independently calculated trees.
Randomization	For kinetic and in vivo studies, biochemical samples were predefined, and no samples were randomly assigned to experimental groups. For phylogenetic analysis, representative sequences from the constructed IleRS database were randomly selected at the family level, according to the GTDB taxonomy, before tree construction. To ensure the robustness of the phylogenetic inferences, family representatives were randomly resampled for each tree.
Blinding	Blinding was omitted from the experimental design because outcomes were determined using established assays and quantitative analyses, leaving little room for subjective observer bias. Similarly, potential investigator bias in the phylogenetic analysis was mitigated by

randomisation of sequences prior to multiple alignment and tree construction.

Reporting for specific materials, systems and methods

We require information from authors about some types of materials, experimental systems and methods used in many studies. Here, indicate whether each material, system or method listed is relevant to your study. If you are not sure if a list item applies to your research, read the appropriate section before selecting a response.

Materials & experimental systems

n/a	Involved in the study
☒	☐ Antibodies
☒	☐ Eukaryotic cell lines
☒	☐ Palaeontology and archaeology
☒	☐ Animals and other organisms
☒	☐ Clinical data
☒	☐ Dual use research of concern
☒	☐ Plants

Methods

n/a	Involved in the study
☒	☐ ChIP-seq
☒	☐ Flow cytometry
☒	☐ MRI-based neuroimaging

Plants

Seed stocks

Report on the source of all seed stocks or other plant material used. If applicable, state the seed stock centre and catalogue number. If plant specimens were collected from the field, describe the collection location, date and sampling procedures.

Novel plant genotypes

Describe the methods by which all novel plant genotypes were produced. This includes those generated by transgenic approaches, gene editing, chemical/radiation-based mutagenesis and hybridization. For transgenic lines, describe the transformation method, the number of independent lines analyzed and the generation upon which experiments were performed. For gene-edited lines, describe the editor used, the endogenous sequence targeted for editing, the targeting guide RNA sequence (if applicable) and how the editor was applied.

Authentication

Describe any authentication procedures for each seed stock used or novel genotype generated. Describe any experiments used to assess the effect of a mutation and, where applicable, how potential secondary effects (e.g. second site T-DNA insertions, mosiacism, off-target gene editing) were examined.
