# Supplementary material for: C-terminal evolutionary remodelling of isoleucyl-tRNA synthetases is a prokaryote-specific strategy for tuning aminoacylation rate
Source: Nat Commun. 2026 Oct 12;17:11575. doi: 10.1038/s41467-026-78241-1 (PMC13752280; doi:10.1038/s41467-026-78241-1)
Supplement: Supplementary file 2 [file 41467_2026_78241_MOESM2_ESM.pdf]

nature portfolio

Peer Review File

C-terminal evolutionary remodelling of isoleucyl-tRNA synthetases is a prokaryote-specific strategy for tuning aminoacylation rate

Corresponding Author: Professor Ita Gruic-Sovulj

Parts of this Peer Review File have been redacted as indicated to maintain the confidentiality of unpublished data.

This file contains all reviewer reports in order by version, followed by all author rebuttals in order by version.

Version 0:

Reviewer comments:

Reviewer #1

(Remarks to the Author)
This manuscript examines the evolutionary diversification of isoleucyl-tRNA synthetase (IleRS) with a focus on the structural and functional roles of its C-terminal domains. While the catalytic and editing domains are well understood, the forces shaping the C-terminal domain architecture remain unclear. Using structural, kinetic, and phylogenetic analyses, the authors revisit the traditional IleRS1 and IleRS2 classification and propose a revised framework that includes a third archaeal clade. Their data suggest that several tRNA recognition elements, including D-loop contacts and the INS subdomain, are not strictly required for activity and may constrain catalytic turnover. The authors extended these findings into an elegant bacterial complementation assay to further validate their findings in cells. Overall, the study provides a compelling revision of IleRS classification using a mix of structural and phylogenetic data supported by strong biochemistry analysis.

Comments to authors:
1)Overall, the phylogeny section is logical and compelling. However, in line 112 “novel” should be replaced with a 3rd clade, especially since the position of IleRS1 to IleRS-A is not entirely stable. Additionally, the choice of root was not justified well in the text. Conceptually it makes sense, but the ancestral claim raised is root-dependent. Were alternative outgroups tested (like other aaRS), is ValRS too divergent for the claims?

2)As a minor comment, the resolution of the PmIleRS2 is rather low, table 1 should include the cc1/2 and I/sigma for the resolution cut off choice. Despite the low resolution the use of unbiased (omit maps) was highly compelling for the conclusions presented.

3)238-243 The description of distinct tRNA binding modes is somewhat misleading. Both PmIleRS2 and SaIleRS1 share a conserved ARD2 framework for tRNA engagement. However, the INS subdomain, which mediates specific contacts with the D-loop, is unique to the IleRS2 C-terminal architecture and is absent in IleRS1. Therefore, the observed differences in D-loop versus D-stem interactions likely reflect the presence or absence of the INS insertion rather than fundamentally distinct binding modes.

4)Figure 3. It would be helpful to include the WT anticodon sequence directly in the figure or legend. While it can be inferred from the single-point mutation labels, providing the reference sequence explicitly would improve readability / interpretation.

5)Lines 269–272. Given that the interpretation relies on distinguishing the activation and aminoacyl transfer steps, explicitly referring to the reaction scheme in Fig. 1A would help readers follow the mechanistic logic. The sentence could also be shortened or split to improve readability.

6)The point mutants and INS deletion are interpreted under a unified model of weakened D-loop interactions; however, INS deletion (and flexible Gly loop substitution) shows only a small reduction in kcat. This suggests a more complex relationship between INS-mediated binding and turnover than implied.

7)The complementation assay was a thoughtful addition to the paper. Perhaps a bit more information for how the assay works will aid more general readers “The complementation assay is conducted in the presence of mupirocin, which inhibits endogenous E. coli IleRS1 and shifts dependence to the expressed PmIleRS2 variants.”

8)Lines 393-400 The proposed evolutionary sequence linking HUP phylogeny to the order of C-terminal domain (CTD) evolution may be somewhat overinterpreted and indirect. In particular, the statement that RS2 CTD architecture evolved first is inferred indirectly from HUP topology and domain organization, and alternative explanations such as domain loss or recombination are not discussed. Similarly, the conclusion that the IleRS1 ancestor lacked an INS subdomain based on its absence in IleRS1 enzymes seems strong, especially given the evidence presented for ongoing and repeated INS loss observed.

9)In the methods the information for how domain boundaries were established should have more information given their importance for the phylogenetic analysis. What were the specific criteria, structural guides, or sequence motifs used to establish these boundaries.

10)For refinement in PHENIX, were secondary structure restraints applied to both the RNA and protein components? The reviewer appreciates the detailed description of the refinement strategy for this challenging low-resolution dataset and the care taken to minimize model bias.

Reviewer #2

(Remarks to the Author)
The manuscript entitled "C-terminal evolutionary remodelling of isoleucyl-tRNA synthetases is a prokaryote-specific strategy for tuning aminoacylation rate," by Modrusan et al., integrates phylogenetic analysis, X-ray crystallography, and steady-state and pre-steady-state kinetics to investigate the evolutionary history and functional significance of the C-terminal domains of isoleucyl-tRNA synthetase (IleRS). The authors have proposed a three-clade classification of IleRS (IleRS-A, IleRS1, IleRS2), reported the first crystal structure of a P. megaterium IleRS2:tRNA complex containing the INS subdomain, and systematically characterized the catalytic consequences of INS subdomain loss in P. megaterium IleRS2:tRNA.

The manuscript is well written and easy to follow. I think it makes a meaningful contribution to our understanding of aminoacyl-tRNA synthetase evolution by proposing a revised three-clade classification of IleRS and linking it to C-terminal domain remodeling and changes in aminoacylation turnover rate. The experimental breadth is reasonable, with integration of structural, kinetic, and phylogenetic approaches. However, the major weaknesses in this manuscript are limited experimental support for the IleRS-A classification, overinterpretation of the X-ray structural data using misleading electron-density maps, and insufficient discussion of the mechanistic gap in explaining the kcat elevation upon INS disruption. The manuscript is novel and interesting, but requires substantive revisions. Here are some comments and suggestions to substantially strengthen the manuscript.

1. The proposal of IleRS-A as a distinct clade is the central phylogenetic contribution of this work, yet it rests on a narrow experimental foundation. Only a single IleRS-A member (M. thermoautotrophicum) has been experimentally characterized (mupirocin sensitivity), with all remaining assignments inferred from sequence features alone.

2. The X-ray crystallographic structural data are overinterpreted. The 5.3 Å resolution cannot resolve the precise interactions between amino acid side chains and tRNA, a fundamental limitation of structural data. Although the article mentions this limitation in the methods section, it is not adequately addressed in the presentation and discussion of the results. Specifically, the key interacting residues (R964, R721, K862, F865, etc.) shown in Figure 2 are inferred from homology comparisons with yeast structures rather than direct observation. This "inference is fact" narrative appears throughout the text. It is recommended that the authors strictly distinguish between "directly observed" and "inferred possible" interactions in their language to avoid misleading readers.

3. The electron density maps are presented with a counter level of 0.6 sigma and a carve radius of 4.6 Å. It is a misleading practice to show a good electron density map. How does this map look with a reasonable counter level of 1 sigma and a carve radius of < 2.0Å?

4. The manuscript does not discuss any crystal contacts that could affect domain conformations.

5. The table of crystallographic data collection and refinement statistics differs in several parameters between the table from the PDB validation report and the one presented in Supplementary Table 1.

6. A cartoon of the complex, color-coded by the crystallographic B-factors, would be useful to include in the supplementary information to access the more dynamic regions in the structure.

7. The core structural comparison in this paper is between PmIleRS2 (5.3 Å resolution, bacterial IleRS2) and SaIleRS1 (PDB: 1FFY). Mechanistic conclusions drawn from this comparison—particularly regarding differences in tRNA D-loop and D-stem binding—may be limited by the significant resolution difference. How can one ensure that the structural differences between the two arise from incompleteness in the PmIleRS2 model due to resolution limitations, rather than from genuine biological differences?
8. The reported ~77° rotation of the INS subdomain upon tRNA binding (Figure S5) is a structurally interesting observation.

At 5.3 Å resolution, however, rigid-body domain placement is heavily influenced by the starting model and refinement protocol. The authors should provide additional validation of this conformational change to confirm that this rearrangement occurs in solution and is not an artifact of crystal packing or molecular replacement bias.

9. The authors used an E. coli mupirocin complementation system to assess the in vivo function of the IleRS2 variant. This design has a serious limitation: it is essentially an overexpression-complementation experiment in a heterologous host. The fact that ΔINS requires high IPTG concentration (200 μM) and tRNA co-overexpression for complementation can be interpreted as either INS being non-essential or as INS loss causing significant functional impairment that requires compensation through overexpression. What would the results be if a similar experiment were conducted in a eukaryotic cell?

10. In single-turnover transfer experiments, what is the efficiency of IleRS: Ile-AMP complex pre-formation, and how does incomplete complex formation affect the reported ktrans values?

11. Line 202 S731 is mentioned in the figure legend (line 202) but does not appear in Figure 2D. It might be S713.

12. Figure 4: Each data point should include error bars if the experiment was performed in triplicate. Also, data should be shown, at least in the supplementary information, for the doubling of the enzyme concentration.

Reviewer #3

(Remarks to the Author)
Journal: Nature Communications
Manuscript number: 700813_0_art_file_12252
Title: C-terminal evolutionary remodelling of isoleucyl-tRNA synthetases is a prokaryote-specific strategy for tuning aminoacylation rate

It is shown that, although IleRS enzymes have traditionally been subdivided into two groups based on their C-terminal domains, they are better described as having split into three evolutionary clades. In addition to bacterial IleRS1 and archaeal/bacterial/eukaryotic IleRS2, it is proposed a new clade of deep-branching archaeal IleRS that they dubbed IleRS-A.
Their phylogenetic analysis indicates that IleRS1 diverged from IleRS-A, likely driven by a replacement of the C-terminal domain. They also found evidence for ongoing C-terminal remodelling, with a concerted loss of the INS subdomain across the prokaryotic tree of life.
To better understand the role of ARD3 and INS, and the forces shaping their evolution, authors solved the crystal structure of P. megaterium IleRS2 in complex with tRNA and performed a detailed kinetic characterisation of ARD3, INS, and tRNA mutants. They found that P. megaterium IleRS2 recognises the anticodon bases with slightly higher fidelity than P. megaterium IleRS1. It is also shown that INS, which is present exclusively in the ARD2 domain of IleRS2 and IleRS-A, first served as a tRNA-binding domain in archaea and bacteria but later evolved into a tRNA recognition element in eukaryotes. In summary, frame the replacement of ARD3 by ZBD to form the IleRS1 clade and the ongoing loss of INS as prokaryotic strategies for increased aminoacylation turnover. However, deeper phylogenetic analysis revealed that some archaeal IleRSs, so far attributed to the IleRS2 clade, actually cluster more closely with IleRS1. This observation raised the question of whether C-terminal domain types correspond to a clade separation. To clarify the evolutionary history of IleRS, a database of 96,219 IleRS sequences spanning bacteria, archaea, and eukaryotes and annotated their protein domain architectures was built.
They found that the standard two-group division scheme does not conform to the clade structure of the phylogenetic tree. In addition to IleRS1 and IleRS2, authors identified a novel clade of archaeal IleRS (IleRS-A). This clade has a C-terminal domain organisation like IleRS2 but has a HUP domain that is more closely related to that of IleRS1 (HUP1).
IleRS-A has a HUP domain that is more closely related to that of IleRS1 (termed HUP-1) but shares a type 2 C-terminal domain organisation with IleRS2, which comprises ARD1-ARD3 and optionally an ~80 amino acid insertion within the ARD2 domain (INS). In contrast, IleRS1 has a treble clef zinc-binding domain (ZBD) and lacks INS, which is referred to as the type 1 C-terminal organization.
They also determined the first crystal structure of an IleRS that contains the more evolutionarily unstable INS domain, making it a particularly useful point of comparison with the tRNA-bound structure of yeast IleRS2 17 and the tRNA- and mupirocin-bound structure of Staphylococcus aureus IleRS1 (SaIleRS1).

I highlight the result that IleRS split into three clades based on the HUP domain and C-terminus.
The present manuscript is a comprehensive analysis that renders a novel view of IleRS phylogeny, showing that the early archaeal split gave rise to the IleRS-A and IleRS2 lineages, followed by the subsequent emergence of IleRS1 within the IleRS-A branch. The deep-rooted position of IleRS-A strongly suggests that its HUP-1 domain more closely resembles the ancestral IleRS synthetic domain.

MAJOR
The scientific methods are rigorous, the objectives are clear, it is well explained and justified, the experimental procedures are modern and sound, the experimental design is appropriate, the statistical analyses are sound, in each step controls are wisely chosen, and the results are at the cutting edge and interesting. Figures are of high quality and well explained.
Maybe the authors would like to add a comment about ARSs research has recently expanded far beyond their canonical roles in translation to emerge as central players in signaling, human disease, and therapeutic interventions.

I think that this manuscript is appropriate for Nature Communications.

Minor
Lines 155-156:
“Thus, current evidence supports a model in which IleRS evolution involved two independent passages from archaeal to bacterial to eukaryotic forms.”

Outstanding and outlandish. Eukarya diverged from Archaea (Lochiarcheota).
Maybe a diagram can be useful.

Line 362: Replace “EcIleRS1” by E. coli IleRS1.
Lines 410-411: “…bacteria encode two tRNA isoacceptors”
Reference 4: Giegé.
Reference 15: Mycobacterium tuberculosis
Reference 16: Insights into Editing from an Ile-tRNA Synthetase Structure
with tRNAIle and Mupirocin.
Reference 19: Söll, D. & Söll, S.
Reference 20: Methanobacterium thermoautotrophicum
Reference 25: Escherichia coli
Reference 26: Escherichia coli
Reference 29: Pseudomonas fluorescens
Reference 30: Staphylococcus aureus
Reference 31: Escherichia coli
Reference 35: Eldred, E.W. & Schimmel, P.R.
Reference 39: Escherichia coli
Reference 40, 42, and 47: tRNAIle

Version 1:

Reviewer comments:

Reviewer #1

(Remarks to the Author)
The authors have done a thorough job addressing my original comments, and I appreciate the thoughtful responses and effort that went into the revision. In particular, the additional phylogenetic analysis, new and revised figures, and revisions to the discussion of the structural and kinetic data have addressed my primary concerns and notably improved the clarity of the manuscript. The new Supplementary Figure 5 is especially helpful and makes the proposed evolutionary model much easier to follow.

Overall, I am satisfied that the authors have adequately addressed the points raised in my initial review, and I have no further concerns.

(Remarks on code availability)

Reviewer #2

(Remarks to the Author)
The authors have adequately addressed my concerns in this revised version of the manuscript. They performed several additional experiments, analyzed the data more rigorously, and discussed the results in greater detail. I believe the revised manuscript now presents compelling research on the C-terminal domains of isoleucyl-tRNA synthetase, which would contribute significantly to the field. The manuscript is acceptable, and I have no further comments.

(Remarks on code availability)
I skipped reviewing this section because I don't consider myself an expert in this coding area.

Reviewer #3

(Remarks to the Author)
Journal: Nature Communications
Manuscript number: 700813_0_art_file_12252
Title: C-terminal evolutionary remodelling of isoleucyl-tRNA synthetases is a prokaryote-specific strategy for tuning aminoacylation rate

Authors have answered all my concerns. They have added new figures that enrich the presentation and reinforce the

conclusions.

(Remarks on code availability)

Open Access This Peer Review File is licensed under a Creative Commons Attribution 4.0 International License, which permits use, sharing, adaptation, distribution and reproduction in any medium or format, as long as you give appropriate credit to the original author(s) and the source, provide a link to the Creative Commons license, and indicate if changes were made.
In cases where reviewers are anonymous, credit should be given to 'Anonymous Referee' and the source.
The images or other third party material in this Peer Review File are included in the article's Creative Commons license, unless indicated otherwise in a credit line to the material. If material is not included in the article's Creative Commons license and your intended use is not permitted by statutory regulation or exceeds the permitted use, you will need to obtain permission directly from the copyright holder.
To view a copy of this license, visit https://creativecommons.org/licenses/by/4.0/

We would like to thank the reviewers for their careful reading of the manuscript and the numerous suggestions that improved its quality. We carefully address all the concerns raised and suggestions given by the reviewers below.

Reviewer #1 (Remarks to the Author):

This manuscript examines the evolutionary diversification of isoleucyl-tRNA synthetase (IleRS) with a focus on the structural and functional roles of its C-terminal domains. While the catalytic and editing domains are well understood, the forces shaping the C-terminal domain architecture remain unclear. Using structural, kinetic, and phylogenetic analyses, the authors revisit the traditional IleRS1 and IleRS2 classification and propose a revised framework that includes a third archaeal clade. Their data suggest that several tRNA recognition elements, including D-loop contacts and the INS subdomain, are not strictly required for activity and may constrain catalytic turnover. The authors extended these findings into an elegant bacterial complementation assay to further validate their findings in cells. Overall, the study provides a compelling revision of IleRS classification using a mix of structural and phylogenetic data supported by strong biochemistry analysis.

Comments to authors:
1) Overall, the phylogeny section is logical and compelling. However, in line 112 “novel” should be replaced with a 3rd clade, especially since the position of IleRS1 to IleRS-A is not entirely stable. Additionally, the choice of root was not justified well in the text. Conceptually it makes sense, but the ancestral claim raised is root-dependent. Were alternative outgroups tested (like other aaRS), is ValRS too divergent for the claims?

*We thank the reviewer for the suggestions. Indeed, outgroup selection can have a significant impact on the inferred root. While it is true that ValRS is more closely related to IleRS (**Supplementary Fig. 2A**), and is thus a more natural choice as an outgroup, we agree that it is worth testing to see if rooting by LeuRS also identifies IleRS-A (or HUP-1 more broadly) as being the primal clade. We provide **the newly added Supplementary Fig. 2** to address this question, as reproduced below. As previously reported, all three of our top-scoring ValRS-rooted trees of the HUP synthetic domain (**Supplementary Fig. 2B**, left) identify IleRS-A as the primal clade within the family. When LeuRS is used as an outgroup (**Supplementary Fig. 2B**, right), the position of the root is less stable: Two of the top-scoring trees identify IleRS1 as the primal clade, and one identifies IleRS-A as the primal clade. Note also that the branch lengths of the outgroups in the LeuRS-rooted trees are significantly longer. Thus, the LeuRS-rooted trees support the result that HUP-1 is the primal HUP form but do neither definitively identify IleRS-A or IleRS1 as the primal clade. Given the stability of the ValRS-rooted trees across several core domains (**Supplementary Fig. 3)**, and the overlap with the LeuRS-rooted trees of the synthetic domain, we remain confident in our ValRS rooting.*

Substituting “novel” for “third” is meaning-preserving with respect to the entailment that a distinct clade exists; both lexical variants carry the same truth conditions in this context. The difference

lies in whether this clade has been identified before. As it has not, we have opted to retain the original wording.

Newly added Supplementary Fig. 2 *with caption:*

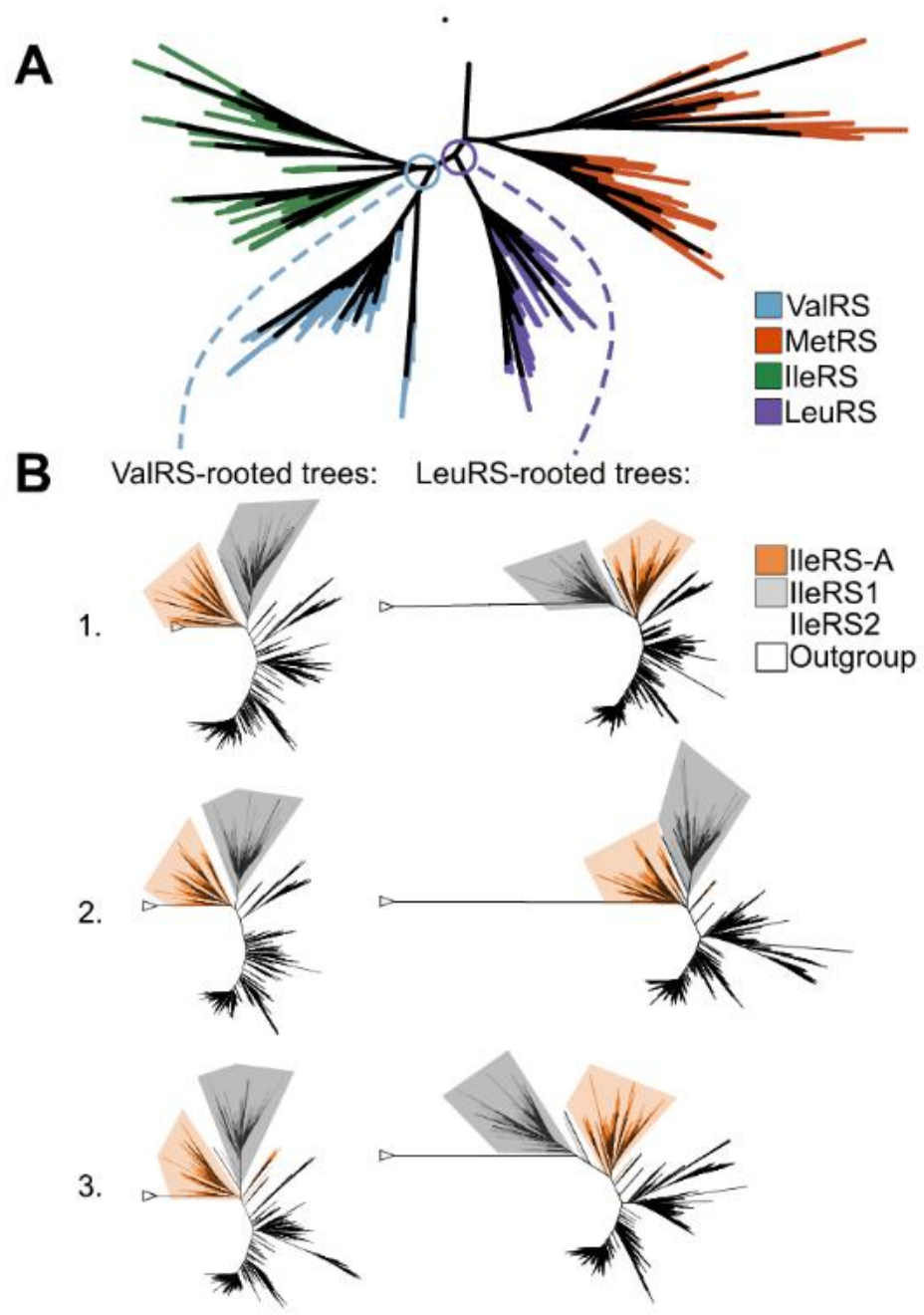

Supplementary Fig. 2: Outgroup selection. *(A) Phylogenetic tree of full-length sequences of MetRS, LeuRS, ValRS, IleRS, CysRS (not shown), and ArgRS (not shown). MetRS, LeuRS, ValRS, IleRS form a cohesive clade, where IleRS is more closely related to ValRS than to LeuRS. For each AARS family, sequences were retrieved from EggNOG v5.0 and clustered to 50% identity. One representative sequence from the top 100 largest clusters within that family was gathered. Sequence alignment, trimming, and tree building were performed as described in the Methods section. (B) The top three trees of the IleRS synthetic domain were assessed by log-likelihood using ValRS (left) or LeuRS (right) as the outgroup. Sequence alignment, extraction of the synthetic domain, and tree building was performed as described in the main text. Note that while ValRS rooting consistently identifies IleRS-A as the primal clade, rooting with LeuRS is less stable, positioning the root in either IleRS1 or IleRS-A, both of which belong to the HUP-1 clade.*

*We have updated the main text as follows (lines 186-193): "Although ValRS is more closely related to IleRS (**Supplementary Fig. 2A**), and thus the natural choice for an outgroup, we also considered whether LeuRS-rooted trees similarly position IleRS-A as the primal IleRS form (**Supplementary Fig. 2B**). Owing to its greater evolutionary distance, the position of root when using LeuRS as an outgroup was less stable, although reliably localised in the HUP-1 clade (IleRS1 or IleRS-A). Taken together with the stable ValRS rooting closest to IleRS-A, we conclude that the last common ancestor of contemporary IleRS synthetic domains was IleRS-A-like."*

2) As a minor comment, the resolution of the PmIleRS2 is rather low, table 1 should include the cc1/2 and I/sigma for the resolution cut off choice. Despite the low resolution the use of unbiased (omit maps) was highly compelling for the conclusions presented.

*We thank the reviewer for this comment. The CC1/2 and I/sigma values were mistakenly dropped from the table and are now added (**updated Table S1**), as obtained from the final XSCALE statistics.*

3) 238-243 The description of distinct tRNA binding modes is somewhat misleading. Both PmIleRS2 and SaIleRS1 share a conserved ARD2 framework for tRNA engagement. However, the INS subdomain, which mediates specific contacts with the D-loop, is unique to the IleRS2 C-terminal architecture and is absent in IleRS1. Therefore, the observed differences in D-loop versus D-stem interactions likely reflect the presence or absence of the INS insertion rather than fundamentally distinct binding modes.

*We agree with the reviewer that the phrasing can be misleading and have changed the text to: "The structural data indicate that $tRNA^{Ile}$ recognition by PmIleRS2 (**Fig. 2A**) differs from SaIleRS1[16]: PmIleRS2 uses INS to interact with the D-loop, while SaIleRS1, lacking the INS subdomain, engages the D-stem of the $tRNA^{Ile}$ via ARD1 (**Supplementary Fig. 7, Supplementary Fig. 14**)."* We have also relocated this sentence to lines 382-385 to improve flow. F*urther, in reply to question 7 from reviewer #2 below, we have newly **added Supplementary Fig. 14**.*

4) Figure 3. It would be helpful to include the WT anticodon sequence directly in the figure or legend. While it can be inferred from the single-point mutation labels, providing the reference sequence explicitly would improve readability/interpretation.

*The WT anticodon sequence (GAU) was added to the title of Fig. 3 and to the figure itself (**updated Fig. 3**)*

5) Lines 269–272. Given that the interpretation relies on distinguishing the activation and aminoacyl transfer steps, explicitly referring to the reaction scheme in Fig. 1A would help readers follow the mechanistic logic. The sentence could also be shortened or split to improve readability.

We have clarified the text, referenced Fig 1A, and split the sentence for improved readability.
*The revised text (lines 335-342): Although the R721A and R964A IleRS2 mutants cannot form Ile-$tRNA^{Ile}$ (**Table 1**), they can participate in the first step of Ile-$tRNA^{Ile}$ formation, Ile activation (**Fig. 1A**), with k_{cat}/K_M values comparable to those of the WT enzyme (**Supplementary Table 3**). This demonstrates that the mutations influence only the second step, aminoacyl transfer (**Fig. 1A**). The transfer of the aminoacyl moiety to tRNA could be assessed independently of the*

activation step by mixing the preformed IleRS:Ile-AMP complex with a limiting amount of $tRNA^{Ile}$ *under single-turnover conditions.*

6) The point mutants and INS deletion are interpreted under a unified model of weakened D-loop interactions; however, INS deletion (and flexible Gly loop substitution) shows only a small reduction in kcat. This suggests a more complex relationship between INS-mediated binding and turnover than implied.

We thank the reviewer for this comment and agree that deleting the entire INS introduces a weak kinetic penalty. Specifically, ΔINS exhibits k_{cat} *(activation) and* k_{trans} *(aminoacyl transfer) that are 3 times smaller than those of the WT. Further, ΔINS lacks the effect observed in single and double INS mutants, which is a 3-fold increase in aminoacylation* k_{cat}*. Thus, a small, reproducible negative effect on catalytic rates (independent of tRNA involvement in the reaction) is observed, in contrast to that observed in INS mutants. Although ΔINS retained the overall fold of the WT, it is possible that whole-subdomain deletion produces a small fold perturbation (not observed by CD (**Supplementary Fig. 15**)) that results in the observed rate effect. We did not explore whether different linkage lengths and insertion positions would eliminate this small kinetic effect. Our primary goal was to show that, even in the absence of the entire INS subdomain, the protein remains highly functional, thereby providing a rationale for the observed natural variability in INS in IleRS2. To address the effect observed only for ΔINS, we added a sentence in the revised version (lines 409-410): "This small reduction in* k_{cat} *is likely due to a small conformational perturbation in ARD2 induced by deletion of the entire INS subdomain."*

7) The complementation assay was a thoughtful addition to the paper. Perhaps a bit more information for how the assay works will aid more general readers "The complementation assay is conducted in the presence of mupirocin, which inhibits endogenous E. coli IleRS1 and shifts dependence to the expressed PmIleRS2 variants."

We thank the reviewer for this remark and have changed the text from "To explore whether INS is required in vivo, we assessed the capacity of INS mutants to rescue E. coli growth in the presence of mupirocin, an inhibitor of its sole endogenous IleRS1."
to
"To explore whether INS is required in vivo, we assessed the capacity of INS mutants to rescue E. coli growth in the presence of mupirocin. Because mupirocin inhibits the sole endogenous IleRS1 of E. coli, growth is dependent on heterologously expressed PmIleRS2 mutants." (lines 422-425)

8) Lines 393-400 The proposed evolutionary sequence linking HUP phylogeny to the order of C-terminal domain (CTD) evolution may be somewhat overinterpreted and indirect. In particular, the statement that RS2 CTD architecture evolved first is inferred indirectly from HUP topology and domain organization, and alternative explanations such as domain loss or recombination are not

discussed. Similarly, the conclusion that the IleRS1 ancestor lacked an INS subdomain based on its absence in IleRS1 enzymes seems strong, especially given the evidence presented for ongoing and repeated INS loss observed.

*We thank the reviewer for the comment, and we now better explain and motivate our proposed model of IleRS evolution. We include a **newly added Supplementary Fig. 5,** which lays out our proposed evolutionary scenario in greater detail and provides an expanded rationale that better communicates the limitations of our historical inference.*

*Newly added **Supplementary Fig. 5** with caption:*

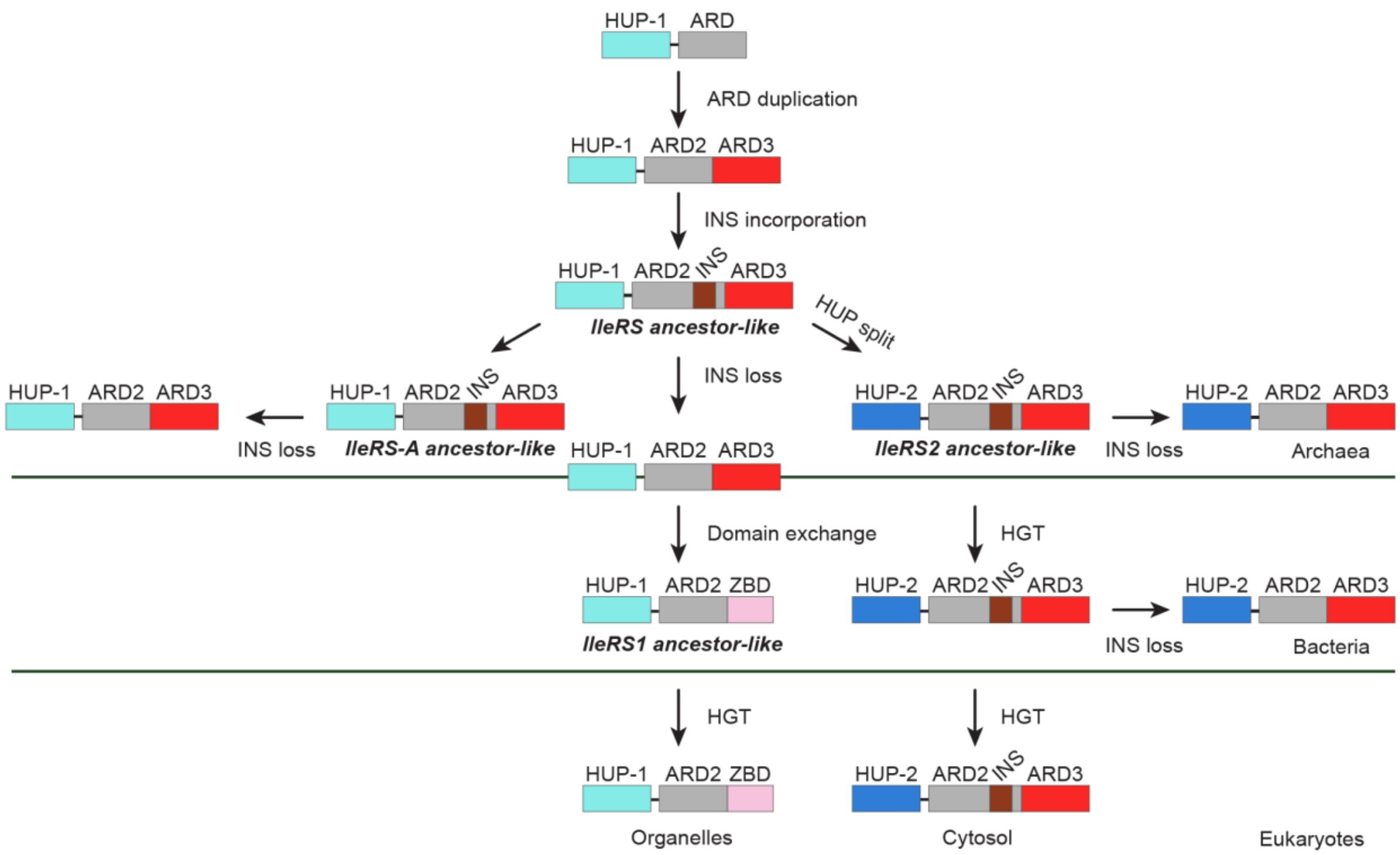

Supplementary Fig. 5: Proposed model of IleRS evolution. *HUP-1 (cyan), HUP-2 (blue), ARD2 (grey), INS (brown), ARD3 (red) and ZBD (light pink) domains are shown as coloured rectangles. The remaining domains (ED, CP1, CP2 and ARD1), whose evolution is not addressed in this model, are represented by a line connecting HUP and ARD. Based on the ValRS-rooted tree, we hypothesise that a HUP-1 domain served as the last common synthetic domain of IleRS. As ARD2 and ARD3 are homologous domains, ARD duplication was the likely first event of C-terminal remodelling. The next stage of C-terminal domain remodelling occurred with the accretion of the INS subdomain within ARD2. We deem the presence of INS in the ARD ancestor domain to be less likely because it would imply that (a) ARD3 had completely lost its copy of the INS subdomain prior to the IleRS ancestor, as no known ARD3 domain has an INS insertion, thereby necessitating an additional event of structural remodelling, and (b) both ARD2 and ARD3 underwent INS loss in nearly equivalent ways, despite the two parent domains playing different structural roles. The presence of INS in the IleRS ancestor is supported by the fact that the majority of contemporary domains within the IleRS-A clade contain the INS subdomain, including the deepest rooting branches. Subsequent transitions – such as the exchange of ARD3 with ZBD in the emergence of IleRS1 – are implied by the phylogenetic tree of the synthetic domain presented in Figure 1. At this resolution, we cannot state*

whether the loss of the INS domain during IleRS1 evolution occurred in archaea or bacteria. HGT stands for horizontal gene transfer.

*We have slightly revised the text in the discussion section (lines 485-499), which now reads: "Our analysis affords a novel view of IleRS phylogeny (**Supplementary Fig. 5**). The deep-rooted position of IleRS-A suggests that the HUP-1 domain more closely resembles an ancestral IleRS synthetic domain. The type 2 C-terminal domain architecture likely evolved first, presumably through duplication of an ancestral ARD domain and subsequent insertion of the INS subdomain into ARD2. An early archaeal split gave rise to the IleRS-A and IleRS2 lineages, followed by the subsequent emergence of IleRS1. The observation that all IleRS1 enzymes lack the INS subdomain indicates that the ancestor of IleRS1 had an ARD2 domain without an INS subdomain. Finally, we note that the emergence of IleRS1 likely coincided with the acquisition of ZBD and the loss of ARD3."*

9) In the methods the information for how domain boundaries were established should have more information given their importance for the phylogenetic analysis. What were the specific criteria, structural guides, or sequence motifs used to establish these boundaries.

The domain boundaries used in this work were adapted from Brkic et al., Nat Commun 14, 5498 (2023), and are based on an analysis of the structure of PmIleRS2 (PDB: 8C8V) and PmIleRS1 (PDB: 8C9E). The boundaries were rechecked against the solved structures of IleRS2 from yeast (PDB: 8WND) and IleRS1 from S. aureus (PDB: 1QU2). These domain boundaries are largely consistent with the Evolutionary Classification of Domains database. We have updated the text to make this point more explicit (lines 591-592): "Using previously reported domain boundaries for PmIleRS2 and PmIleRS1[22], reference IleRS crystal structures (PDB:8C8V[22], PDB:8C9E[22], PDB:8WND[17], PDB:1QU2[16]),…."

10) For refinement in PHENIX, were secondary structure restraints applied to both the RNA and protein components? The reviewer appreciates the detailed description of the refinement strategy for this challenging low-resolution dataset and the care taken to minimize model bias.

We thank the reviewer for their positive comment. Yes, secondary restraints were applied for both the protein (Ramachandran, secondary structure, NCS) and the tRNA (base pair, stacking pair, NCS). A clarifying sentence, "In order to prevent overfitting, secondary structure restraints were applied for both the protein (Ramachandran, secondary structure, torsion-angle NCS) and the tRNA (base pair, stacking pair, torsion-angle NCS)", is now added in the methods section (lines 712-715).

Reviewer #2 (Remarks to the Author):

The manuscript entitled "C-terminal evolutionary remodelling of isoleucyl-tRNA synthetases is a prokaryote-specific strategy for tuning aminoacylation rate," by Modrusan et al., integrates phylogenetic analysis, X-ray crystallography, and steady-state and pre-steady-state kinetics to investigate the evolutionary history and functional significance of the C-terminal domains of isoleucyl-tRNA synthetase (IleRS). The authors have proposed a three-clade classification of IleRS (IleRS-A, IleRS1, IleRS2), reported the first crystal structure of a P. megaterium IleRS2:tRNA complex containing the INS subdomain, and systematically characterized the catalytic consequences of INS subdomain loss in P. megaterium IleRS2:tRNA.

The manuscript is well written and easy to follow. I think it makes a meaningful contribution to our understanding of aminoacyl-tRNA synthetase evolution by proposing a revised three-clade classification of IleRS and linking it to C-terminal domain remodeling and changes in aminoacylation turnover rate. The experimental breadth is reasonable, with integration of structural, kinetic, and phylogenetic approaches. However, the major weaknesses in this manuscript are limited experimental support for the IleRS-A classification, overinterpretation of the X-ray structural data using misleading electron-density maps, and insufficient discussion of the mechanistic gap in explaining the kcat elevation upon INS disruption. The manuscript is novel and interesting, but requires substantive revisions. Here are some comments and suggestions to substantially strengthen the manuscript.

1. The proposal of IleRS-A as a distinct clade is the central phylogenetic contribution of this work, yet it rests on a narrow experimental foundation. Only a single IleRS-A member (M. thermoautotrophicum) has been experimentally characterized (mupirocin sensitivity), with all remaining assignments inferred from sequence features alone.

We thank the reviewer for this comment. To address it, we produced two IleRS-A enzymes: one from the Woesearchaeota archaeon (WaIleRS-A) and another from the Candidatus Micrarchaeota archaeon (MaIleRS-A) and one archaeal IleRS2 enzyme from the Candidatus Diapherotrites archaeon (DaIleRS2). The positions of the proteins on the tree are given in the ***updated Fig 1****.*

We assessed the sensitivity of these IleRS enzymes towards mupirocin, as shown in the ***newly added Supplementary Fig. 4D****. The results clearly indicate that WalleRS-A is sensitive to mupirocin, but that both MalleRS-A and DalleRS2 are resistant. Thus, WalleRS-A and DalleRS2 behaved as expected, and MalleRS-A did not. This result may suggest variability in mupirocin sensitivity even within IleRS-A, possibly related to other organismal features, the environment, and evolutionary selection.*

We therefore questioned whether tolerance to swapping the 1st and 3rd positions in the HIGH signature motif could better reflect clade-specific features. We previously showed that this swap can occur naturally in IleRS2, but is neither found nor tolerated upon its introduction in IleRS1 (Brkic et al., Nat Commun 14, 5498 (2023)). Thus, the GIHT-WaIleRS-A, GVHT-MaIleRS-A, and

*GAHN-DaIleRS2 mutants were produced and tested for Ile activation (**Supplementary Fig. 4A-C**). Both IleRS-A mutants were strongly inactivated, as previously shown for IleRS1 enzymes from E. coli and P. megaterium, while archaeal IleRS2 activity was only slightly impaired by the exchange, analogous to that previously observed for IleRS2 from T. thermophilus and P. megaterium.*

We encountered significant challenges in expressing archaeal proteins and are currently developing improved expression assays for an expanded kinetic analysis.

*Newly added **Supplementary Fig. 4** with caption:*

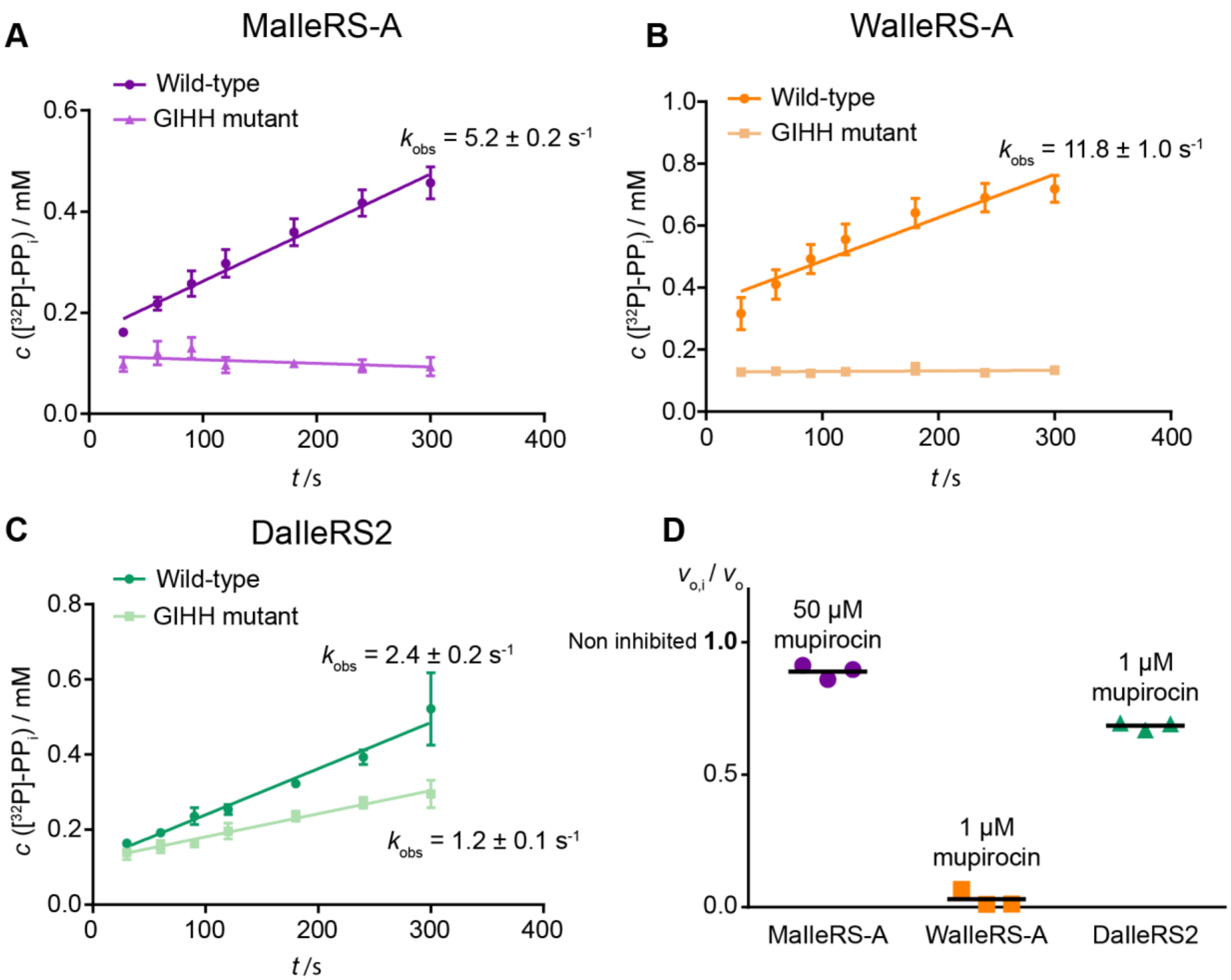

Supplementary Fig. 4: Tolerance to the GIHH motif and sensitivity to mupirocin by archaeal enzymes. *(A-C) The introduction of the GIHH motif inactivated IleRS-A from Woesearchaeota archaeon (WaIleRS-A) and Candidatus Micrarchaeota archaeon (MaIleRS-A), while IleRS2 from Candidatus Diapherotrites archaeon (DaIleRS2) remained highly active. GIHH represents the non-canonical motif in which the 1st and 3rd positions are swapped. Due to sequence variability of the HIGH motif, the corresponding non-canonical motif sequences in these enzymes are GIHT (WaIleRS-A), GVHT (MaIleRS-A), and GAHN (DaIleRS2). For simplicity, we collectively refer to these motif variants as the GIHH throughout the manuscript. (D) Inhibition was tested at 1 µM mupirocin. At that value, WaIleRS-A was almost completely inhibited, showing sensitivity to mupirocin similar to IleRS1 enzymes, which have Ki*

values in the nM or even pM range[1–4]. As expected, DaIleRS2 was not significantly inhibited, consistent with the Ki for IleRS2 enzymes being in the µM range[4–6]. Surprisingly, MaIleRS-A was not inhibited even with 50 µM mupirocin. The rates in (A-D) were obtained by measuring 32[P]ATP-PPi exchange, a standard assay for testing the amino acid activation step, i.e., the first step of aminoacylation[7]. The ATP concentration was 4 mM, and the Ile concentration was 10 x K_M (Ile) for the corresponding enzyme when testing WT and GIHH mutant activity in (A-C) or 1 x K_M (Ile) when testing mupirocin inhibition (D). v_0 and $v_{0,i}$ are the initial velocities in the absence and presence of mupirocin, respectively. $v_{0,i}/v_0$ is the fractional activity in the presence of mupirocin at the stated concentration. The data represent three independent experiments. For panels (A–C), each experiment was fitted individually, and the resulting k_{obs} values were used to calculate the mean ± SEM shown in the figure. For visualisation of the error bars, data points from all three experiments were fitted together. For panel (D), fractional activity was determined for each independent experiment, and the mean value is indicated by the horizontal line.

*The production and analysis of WT enzymes and GIHH mutants are described in the methods section (lines 769-772, 794-805); experimental data are shown in **Supplementary Fig. 4**, presented above, and in the **updated Figure 1**.*

***The final text in the revised version** (lines 126-151) is as follows:*
*Plotting the occurrence of the primary non-canonical HIGH motif, referred to here as GIHH, demonstrates that it is a feature of HUP-2[22] but is entirely absent from IleRS-A and IleRS1 (**Fig. 1D**). In the GIHH motif, the highly conserved first and third amino acid positions of the canonical HIGH motif are swapped. Note that sequence variability exists within both motifs; thus, these terms are used herein as group names rather than exact sequence.*

*To determine whether our updated classification scheme meaningfully relates to properties of the synthetic domain, we identified three archaeal enzymes for biochemical characterisation: the IleRS-As from Woesearchaeota (WaIleRS-A) and Candidatus Micrarchaeota (MaIleRS-A), and the IleRS2 from Candidatus Diapherotrites (DaIleRS2). Based on the properties of the characterised HUP-1 synthetic domains to date[22,25–28] we predict that IleRS-A enzymes should (a) not tolerate the non-canonical GIHH motif and (b) exhibit sensitivity to mupirocin. As expected, both WaIleRS-A and MaIleRS-A were fully inactivated when the 1st and 3rd positions of their respective HIGH motifs were swapped (**Supplementary Fig. 4A and B**), whereas DaIleRS2 exhibited only a twofold lower rate of Ile activation (**Supplementary Fig. 4C**), similar to that of previously reported for IleRS2 enzymes[22].*

*Next, we tested whether these archaeal enzymes responded to mupirocin as expected. As observed for various IleRS1s[25–28] and the IleRS-A from Methanobacterium thermoautotrophicum[20], we found that WaIleRS-A is sensitive to mupirocin (**Supplementary Fig. 4D**). Likewise, DaIleRS2 was resistant to mupirocin (**Supplementary Fig. 4D**), as expected for an IleRS2[15,21,22,27,29]. Interestingly, MaIleRS-A was also resistant to high µM concentrations of mupirocin (**Supplementary Fig. 4D**). This unexpected result suggests greater variability in mupirocin resistance than expected. Nevertheless, our biochemical data generally support the assignment of an IleRS-A and IleRS1 clades as having biochemically similar synthetic domains, which we refer to here as the HUP-1 synthetic domain (**Fig. 1**).*

2. The X-ray crystallographic structural data are overinterpreted. The 5.3 Å resolution cannot resolve the precise interactions between amino acid side chains and tRNA, a fundamental limitation of structural data. Although the article mentions this limitation in the methods section, it is not adequately addressed in the presentation and discussion of the results. Specifically, the key interacting residues (R964, R721, K862, F865, etc.) shown in Figure 2 are inferred from homology comparisons with yeast structures rather than direct observation. This "inference is fact" narrative appears throughout the text. It is recommended that the authors strictly distinguish between "directly observed" and "inferred possible" interactions in their language to avoid misleading readers.

We believe that although the resolution of the X-ray data is limited to 5.3 Å, it is worth including in the manuscript, especially because we took great care to process the data, refine the structure, and present it in a way that accounts for its limitations. However, we agree with the reviewer that we should more carefully distinguish between homology modelling and direct observation and have adjusted the text (see below) and the legend to Fig. 2 accordingly. To better communicate the kinetic support for the structural information, we plotted the fold change in k_{cat} relative to WT for the tested residues in the ***updated Fig. 2****.*

The revised text *(lines 262-312): When compared to the structure of PmIleRS2 without $tRNA^{Ile}$ (PDB: 8C8V)[22,] we find that $tRNA^{Ile}$ binding promotes a ~77° rotation and ~28 Å displacement of the INS subdomain in all four complexes in the asymmetric unit (****Supplementary Fig. 9 and Supplementary Fig. 10****), enabling its engagement with the D-loop by motion around a hinge in the ARD2 domain (****Supplementary Fig. 7, Supplementary Fig. 11****). In this INS subdomain conformation, K862 and F865 become positioned close to bases U20 and U21 of the $tRNA^{Ile}$, suggesting a role in phosphate backbone recognition (****Fig. 2B****).*

*To recognise the anticodon loop, our model indicates that ARD3 (****Fig. 2A****) may be involved in recognition of the first anticodon base, G34, through the pocket formed by the conserved residues Q961, R964, K965, and L969 (****Fig. 2C****), while the second and third anticodon nucleotides, A35 and U36, may be recognised by ARD1 residues R721, S713, F651 and N647 (Fig. 2D). In both cases, comparison with the high-resolution yeast IleRS2:$tRNA^{Ile}$ structure supported the assignment of the recognition pockets (****Supplementary Fig. 12****).*

*To corroborate the structural data, we investigated the putative functional role of these residues in $tRNA^{Ile}$ recognition by PmIleRS2 using mutagenesis and steady-state kinetic analysis. Substitution of R964, shown to participate in G34 recognition in yeast (****Supplementary Fig. 12****) and possibly also in PmIleRS2 (****Fig. 2C****), abolished the capacity of PmIleRS2 to charge $tRNA^{Ile}$ with Ile, confirming its key role in G34 recognition. The aminoacylation rate for the R964A mutant was below 0.001 s^{-1}, demonstrating a 10^3-fold drop relative to the wild-type (WT) enzyme (****Table 1****). The barely detectable activity of the R964A mutant precluded the determination of a K_M ($tRNA^{Ile}$). We next mutated two highly conserved ARD3 residues located nearby G34, Q961 and K965. The catalytic efficiency (k_{cat}/K_M) for the Q961A and K965A mutants decreased 10-fold relative to WT (****Table 1****), suggesting a supporting role in G34 recognition together with R964.*

*The next two anticodon bases, A35 and U36, may be recognised by the strictly conserved R721 within the ARD1 domain of PmIleRS2 (**Fig. 2D**), as inferred by comparison with the yeast IleRS2 structure (**Supplementary Fig. 12**). Similar to R964A, the R721A mutation decreased the aminoacylation rate to below 0.001 s^{-1} (**Table 1**). Mutation of ARD1 residues N647, F651, and S713 to alanine (**Fig. 2D**) decreased the aminoacylation k_{cat}/K_M by 5 to 30-fold, mainly via a kcat effect (**Table 1**). These results demonstrate that R721 is critical for A35 and U36 recognition, with other residues in ARD1 further refining PmIleRS2 specificity.*

Anticodon recognition by PmIleRS2 is more stringent than by PmIleRS1

*Although IleRS1 and IleRS2 both utilise ARD1 and ARD3 to read the anticodon triplet, SaIleRS1 recognises the G34 base via backbone and stacking interactions, whereas yeast IleRS2 uses specific side-chain recognition (**Supplementary Fig. 12**), which is likely preserved in PmIleRS2. Contacts with A35 involve analogous residues from ARD1 in both IleRS1 and IleRS2 enzymes, whereas the reading of U36 differs between them. Protein contacts to U36 present in yeast IleRS2 (**Supplementary Fig. 12**) and likely in PmIleRS2, where they are conserved on sequence level, are replaced in SaIleRS1 by interactions with U33 of the $tRNA^{Ile}$ itself (**Supplementary Fig. 12**). Thus, the structural data indicate that IleRS2 exhibits more specific $tRNA^{Ile}$ anticodon recognition than IleRS1.*

***The revised legend for Fig. 2**.*

*PmIleRS2 is colored according to domain borders, and the 2mFo–DFc Fourier difference map (grey mesh) is carved 4.6 Å around the model and contoured at 1.0 σ. **B-D**) D-loop and anticodon recognition by PmIleRS2. The position of the INS subdomain insertion into ARD2 suggests interactions with the D-loop in the vicinity of U20 and U21 nucleotides. (B). The pocket formed by residues R964, L969, Q961, and K965 within ARD3 is found in proximity of the first anticodon base, G34 (C), while the second and third bases, A35 and U36, are close to residues R721, N647, F651, and S713 in the ARD1 subdomain (D). **E)** Kinetic analysis of the participation of these residues in $tRNA^{Ile}$ recognition by mutagenesis (**Table 1**), with the fold change in k_{cat} relative to the WT plotted. **F)** Sequence alignment showing that the residues involved in anticodon recognition in PmIleRS2 are highly conserved, in contrast to those involved in D-loop recognition. Residues that are mutated and experimentally tested are marked with arrows.*

3. The electron density maps are presented with a counter level of 0.6 sigma and a carve radius of 4.6 Å. It is a misleading practice to show a good electron density map. How does this map look with a reasonable counter level of 1 sigma and a carve radius of < 2.0Å?

In the originally submitted manuscript, we had made the following statement in the legend of Figure 2: "… PmIleRS2 is colored according to domain borders, and the $2mF_o–DF_c$ Fourier difference map (grey mesh) is carved 4.6 Å around the model and contoured at 0.9 σ (panels A,C and D) or 0.6 σ (panel B)." We had chosen a lower contour level for panel (B) because the INS domain is less well resolved.

To simplify the description of the figure, we now visualise the $2mF_o–DF_c$ Fourier difference map in all panels at the requested contour level of 1.0 σ. The optimal map carving radius around molecules A (protein) and a (tRNA) in the asymmetric unit is still set to 4.6 Å, in order to not cut into the map surface at the chosen contour level, but to prevent overlap with the map areas of the neighbouring molecules in the ASU. The visualised cutout is not manipulated in any way. At the 2 Å carving radius and 1 σ level suggested by the reviewer, the map would represent an artificial cutout of a map segment and simply reproduce the outer shape of the model. ***An updated Fig. 2*** *has been introduced as well as the changes introduced in the figure legend.*

Furthermore, to better visualise the INS subdomain and also account for the issues raised below, we added ***a new Supplementary Fig. 7*** *(introduced in line 228), where the B-factors, unbiased $mF_o–DF_c$ OMIT and $2mF_o–DF_c$ Fourier difference maps are shown.*

Newly added ***Supplementary Fig. 7*** *with caption:*

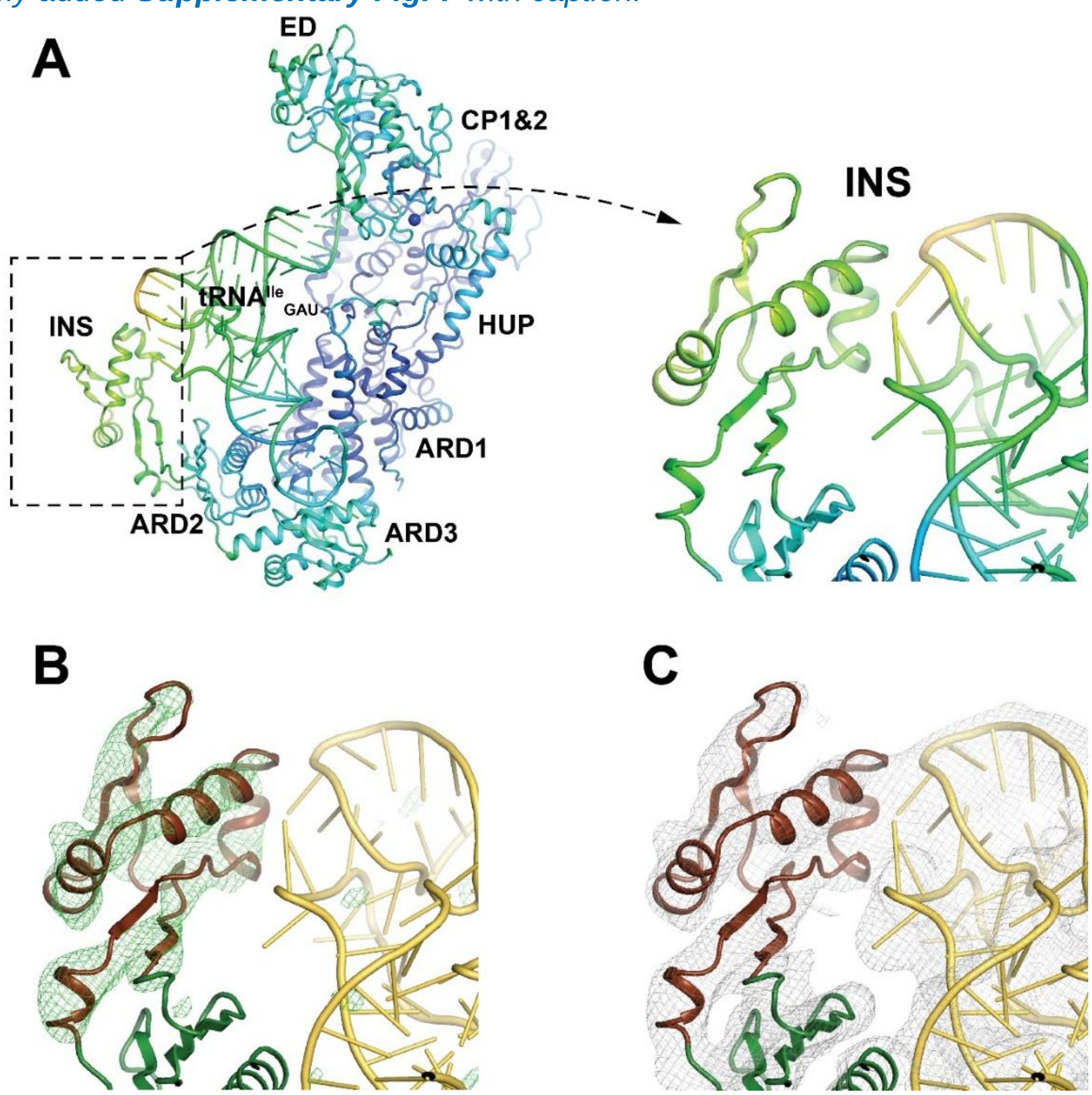

Supplementary Fig. 7: Direct experimental evidence for the binding of the INS subdomain to the elbow region of the tRNA^{Ile} in the crystal structure. *(A) Representative B-factor distribution in the PmIleRS2:tRNA^{Ile}:ATP complex (exemplified here by chains A,a in the ASU), indicating that the INS subdomain (inset) and tRNA^{Ile} elbow regions are more flexible than the remaining parts of the complex. The*

B-factor distribution of all chains in the ASU is visualised in ***Supplementary Fig. 11.*** *(B) The INS subdomain is clearly recognisable in an unbiased mFo–DFc OMIT map (green mesh, contoured at 3.0 σ), allowing precise rigid body docking with only minor positional errors. The map was obtained by omission of the INS subdomain from the model, random shift of all remaining atoms by 0.25 Å and resetting B-factors to remove model bias, followed by refinement to convergence. (C) The INS subdomain is clearly visible in the 2mFo–DFc Fourier difference map, although at a lower contour level than the rest of the molecule (here shown at 0.5 σ as a grey mesh, contoured 4.6 Å around the model).*

[editorial note: reviewer-only data redacted]

4. The manuscript does not discuss any crystal contacts that could affect domain conformations.

From the domain rearrangements observed when the four IleRS2:tRNA copies in the ASU are superimposed, and by comparison with the yeast structure (PDB 8WND, chains A,T), we observe the largest conformational differences in the areas of ARD3 or INS domains, the latter changing position together with the bound tRNA elbow region (now visualised in a ***newly added***

***Supplementary Fig. 10**, panels A-E, introduced in line 266). Although these distortions likely arise from contacts with neighbouring complexes in the crystal lattice, the overall similar arrangement of protein domains and tRNAs suggests that the conformational changes are not functionally relevant, and therefore, we decided to not further discuss them in the main text of the manuscript. However, we have now added a short extra section" Initial structural comparison of the four PmIleRS2:tRNA^{Ile} complexes in the asymmetric unit", in the methods (lines 732-742) stating that the complexes display intrinsic flexibility in certain areas, which is also reflected by the B-factor distribution (see below, **newly added Supplementary Fig. 11**).*

Newly added Supplementary Fig. 10 with caption:

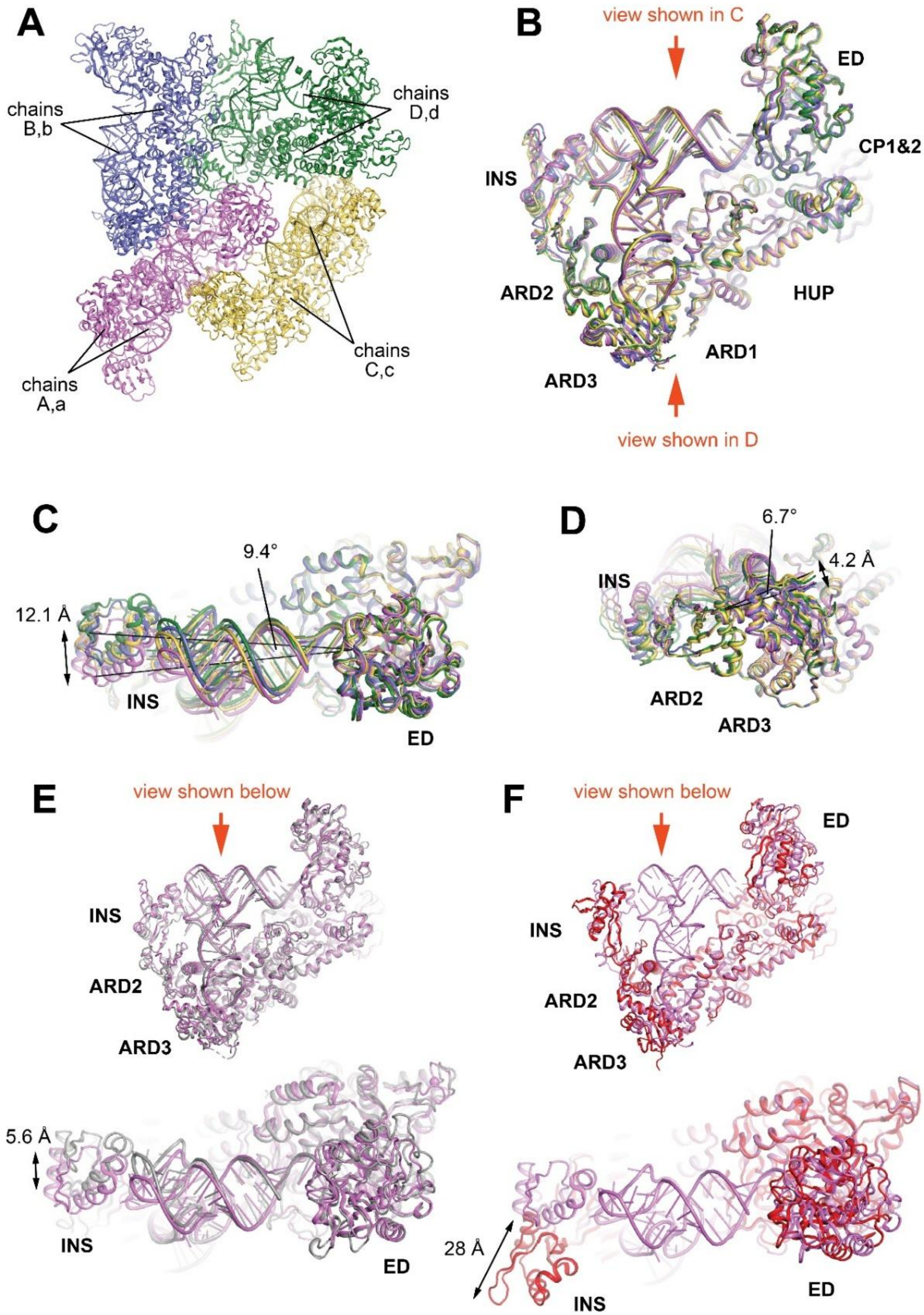

Supplementary Fig. 10: Local domain rearrangements between the four PmIleRS2:tRNA^{Ile}:ATP complexes in the asymmetric unit of the crystal. *A comparative analysis of PmIleRS2 domain rearrangements within and between the four copies in the ASU revealed only moderate conformational*

differences, indicating that these are likely induced by crystal packing and do not affect the overall architecture of the IleRS2:tRNA^{Ile}:ATP complexes. This suggests intrinsic flexibility rather than distinct functional states. ***(A)*** *Localisation of the four IleRS2:tRNA^{Ile}:ATP complexes in the ASU.* ***(B)*** *In the side view of the four superimposed complexes, the domains are very similarly arranged.* ***(C)*** *The top view reveals a swinging motion of the INS subdomain in concert with the bound tRNA elbow around a hinge point localised at the CCA-3' end of the tRNA.* ***(D)*** *The bottom view shows slight repositioning of the ARD3 domain.* ***(E)*** *Superposition of the yeast structure (grey, PDB 8WND8, chains A,T) onto chains A,a (magenta) reveals a conformational change in a similar range.* ***(F)*** *Compared to the tRNA-free structure (red, PDB 8C8V5), the repositioning of the INS domain is more dramatic, suggesting a flexibly attached INS domain that becomes positioned in a specific manner only in the presence of tRNA^{Ile}.*

5. The table of crystallographic data collection and refinement statistics differs in several parameters between the table from the PDB validation report and the one presented in Supplementary Table 1.

We appreciate the careful comparison of the tables and now also report the number of reflections used during refinement by PHENIX in an extra line, as well as the data completeness, the number of molecules / ASU, the overall B-factor and the one from the Wilson plot. We further specify the I/σ and CC1/2 obtained from XSCALE, as suggested by reviewer #1.

6. A cartoon of the complex, color-coded by the crystallographic B-factors, would be useful to include in the supplementary information to access the more dynamic regions in the structure.

We added a ***new Supplementary Fig. 11*** *(introduced in line 268) showing the B-factor distribution in the ASU, the ellipsoids from the TLS refinement, as well as a side-by-side comparison of the B-values of each of the four complexes in the ASU. The distribution of the B-values in the elbow region of the tRNAs and the bound INS domains reflects the increased mobility and the conformational heterogeneity in that area when the four complexes in the ASU are superimposed.*

Newly added ***Supplementary Fig. 11*** *with caption:*

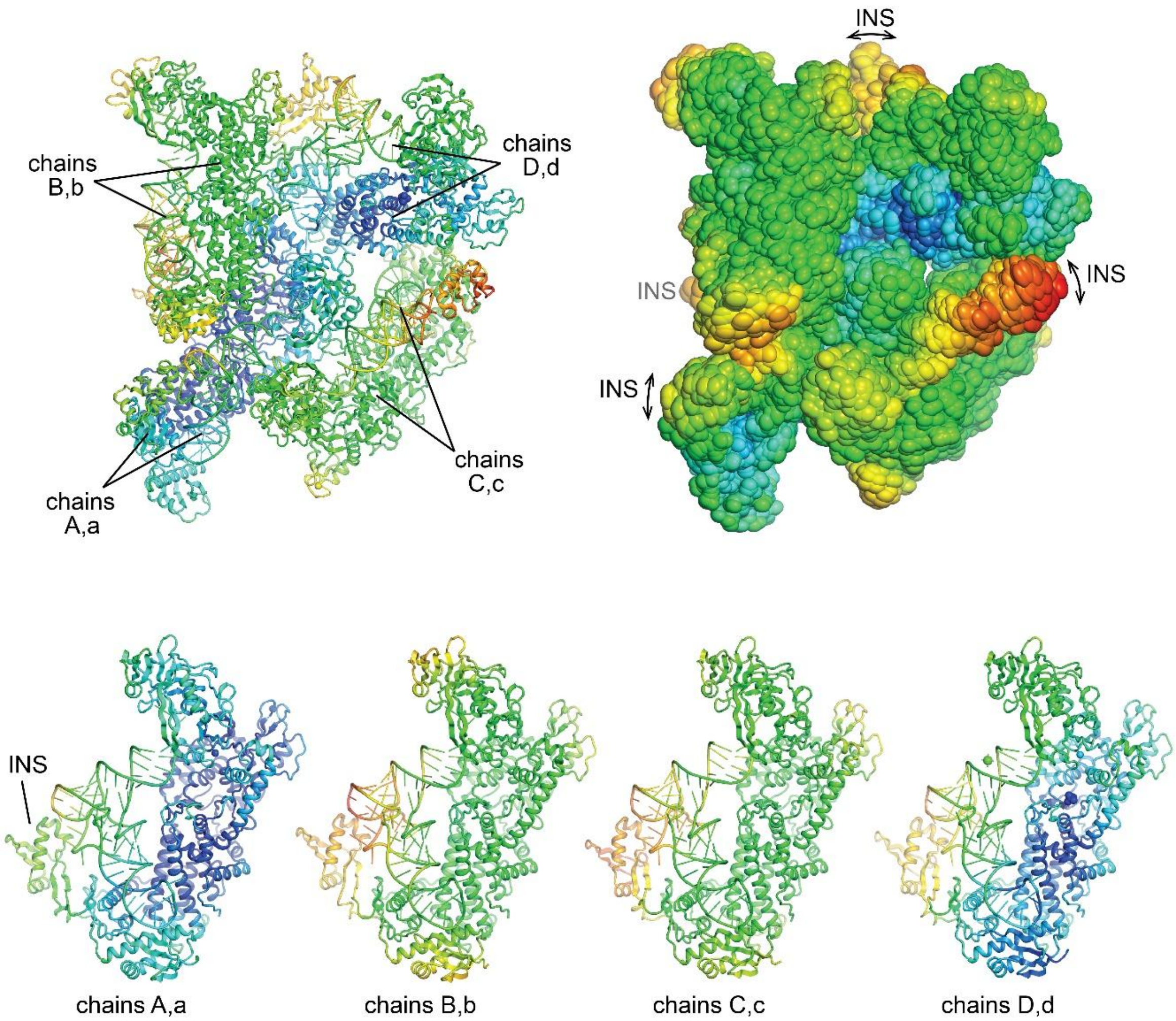

Supplementary Fig. 11: B-factor distribution in the four PmIleRS2:tRNA^{Ile}:ATP complexes as arranged within the ASU or as side-by-side comparison*. The INS subdomain bound to the tRNA^{Ile} elbow region represents the most flexible part, reflecting the conformational heterogeneity in that area when the four complexes are superimposed. The directional mobility can be recognised in the ellipsoidal anisotropic B-factor representation obtained by TLS refinement***.**

7. The core structural comparison in this paper is between PmIleRS2 (5.3 Å resolution, bacterial IleRS2) and SaIleRS1 (PDB: 1FFY). Mechanistic conclusions drawn from this comparison—particularly regarding differences in tRNA D-loop and D-stem binding—may be limited by the significant resolution difference. How can one ensure that the structural differences between the two arise from incompleteness in the PmIleRS2 model due to resolution limitations, rather than from genuine biological differences?

Even at a limited resolution of 5.3 Å, the most important contacts between the tRNA and IleRS2 can be clearly distinguished from those in the better-resolved PDB 1FFY. This is especially true for tRNA backbone contacts and the shifted ARD1 domain. Further, the area of the anticodon is drastically rearranged. To better visualise this, we generated a ***newly added Supplementary Fig***

***14**, where the tRNA of 1FFY is aligned onto our structure, together with the 2mFo–DFc Fourier difference map contoured at 1σ.*

*Text edits were introduced in response to reviewer #1's question 3, and **Supplementary Fig 14** is cited (line 385).*

*Newly added **Supplementary Fig 14** with caption:*

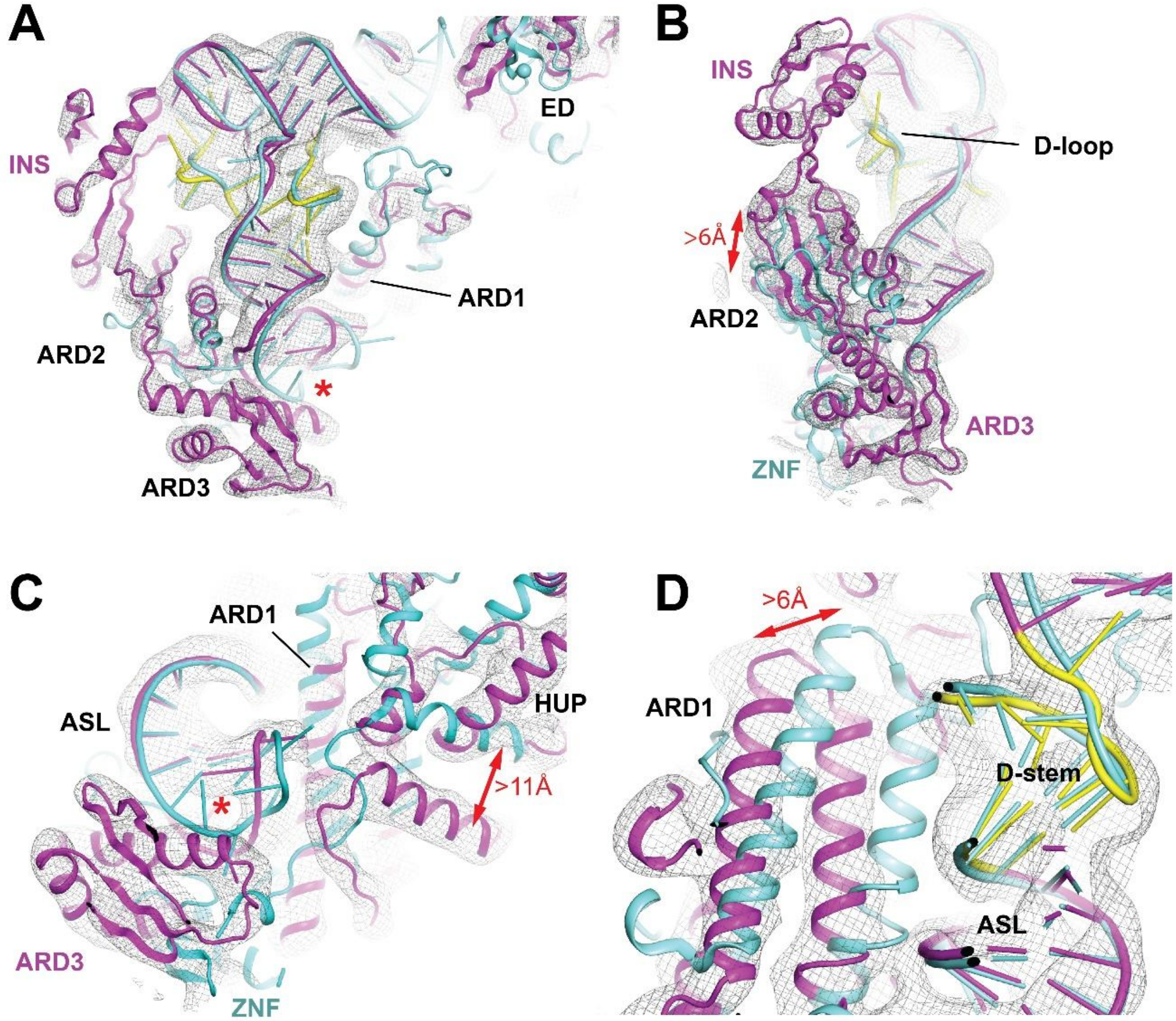

***Supplementary Fig. 14: Structural differences in tRNA^{Ile} recognition between PmIleRS2 and SaIleRS. (A, B)** Different views showing the superimposed SaIleRS (PDB 1FFY9, cyan) and PmIleRS2 (magenta), with the 2mFo–DFc Fourier map (grey mesh) contoured at 1.0 σ and the tRNA^{Ile} D-stem and D-loop areas highlighted in yellow. The INS subdomain is present only in PmIleRS2, where it binds the D-loop of tRNA^{Ile}, while the ARD3 replaces the zinc-binding domain (ZBD) present in SaIleRS, with the ARD1 and ARD2 domains considerably shifted. As a reference for superposition, the tRNA acceptor arms were aligned. **(C)** The anticodon loop (red asterisk) adopts an alternative conformation when bound to PmIleRS2 compared to SaIleRS, while the HUP and ARD1 domains are shifted upwards. **(D)** In PmIleRS2, the ARD1 domain is detached from the D-stem of the tRNAIle, while in SaIleRS it forms close contact.*

8. The reported ~77° rotation of the INS subdomain upon tRNA binding (Figure S5) is a structurally interesting observation. At 5.3 Å resolution, however, rigid-body domain placement is heavily influenced by the starting model and refinement protocol. The authors should provide additional validation of this conformational change to confirm that this rearrangement occurs in solution and is not an artifact of crystal packing or molecular replacement bias.

*As shown in the **newly added Supplementary Fig. 7** and the accompanying Extra Figure for reviewers, the presence and location of the INS domain can be clearly established by calculating unbiased OMIT maps for all four IleRS2:tRNA complexes in the ASU, allowing rigid body docking of this domain (taken from PDB 8C8V, a similar IleRS2 structure without tRNA) with minor alignment errors. Superposition of all four complexes in the ASU reveals that this domain can become directionally displaced together with the elbow region of the tRNA, without detachment of the interaction interfaces (now shown in the **newly added Supplementary Fig. 10**). Further, in the yeast IleRS2:tRNA complex (PDB 8WND), the INS domain is positioned within a similar conformational range. These observations are indicative of intrinsic flexibility in the tRNA-bound state rather than a molecular mechanism. However, the position of the INS domain in the tRNA-free IleRS2 structure, where it is held in place by a crystal contact with a symmetry-related molecule, demonstrates that it can be repositioned drastically, suggesting that this domain is flexibly attached in solution and becomes captured in a specific conformational state upon tRNA binding, but within a certain range of motion.*

9. The authors used an E. coli mupirocin complementation system to assess the in vivo function of the IleRS2 variant. This design has a serious limitation: it is essentially an overexpression-complementation experiment in a heterologous host. The fact that ΔINS requires high IPTG concentration (200 μM) and tRNA co-overexpression for complementation can be interpreted as either INS being non-essential or as INS loss causing significant functional impairment that requires compensation through overexpression. What would the results be if a similar experiment were conducted in a eukaryotic cell?

We thank the reviewer for this comment, but we cannot see an advantage to testing complementation in a eukaryotic cell, since the enzymes are bacterial rather than eukaryotic. Furthermore, eukaryotic cells are less sensitive to mupirocin. Eukaryotic cytosolic IleRS are type 2; K_i for mupirocin for yeast IleRS is 8 μM, while Ki for PmIleRS2 is 1 μM. Thus, PmIleRS2 cannot complement cytosolic IleRS in yeast. The mitochondrial enzymes are of type 1 and thus likely sensitive, but entry of mupirocin into mitochondria might be restricted. Moreover, mitochondrial complementation could be significantly impaired by improper folding of the imported bacterial enzyme and by the unique features of mitochondrial translation.

We think the presented complementation assay supports the main message that the INS subdomain could have been lost "relatively easily" during evolution. ΔINS successfully complemented inhibited E. coli IleRS when produced at a high expression level and in the presence of co-overexpressed tRNA. Interpreting the "overexpression conditions" could be relative, as the reviewer points out. We think it should be compared with anticodon variants, which, even under these "harsh" complementation conditions, were completely inactive, illustrating

significant functional impairment. Importantly, INS mutants (single and double) complement growth at minimal IPTG, as does the WT enzyme. Harsher conditions were required for ΔINS relative to the INS point mutants, because, as noted in response to question 6 of reviewer #1, removing the entire INS induces a small effect on the rate, likely due to a minor fold change.

We agree with the reviewer that complementation assays have their limitations. Accordingly, we conducted them more thoughtfully than the usual procedure by testing several IPTG concentrations to distinguish between weak and strong complementation and co-expression with the homologous tRNA. Given the use of all controls and various conditions, and the strong correlation with in vitro kinetic data, we think it provides a reasonably accurate picture of the in vivo activity of the variants.

10. In single-turnover transfer experiments, what is the efficiency of IleRS: Ile-AMP complex pre-formation, and how does incomplete complex formation affect the reported ktrans values?

There are several issues inherent to single-turnover kinetics and to our system that are related to this question, so the reported k_{trans} should not be affected by incomplete complex formation (if it occurs at all). First, single-turnover kinetics are performed at a higher enzyme concentration than tRNA; in our case, IleRS was 10 to 50 µM and tRNA was 1 to 5 µM. Under these conditions, the fraction of "active or AA-AMP bound" enzyme is higher than that of the tRNA, and the reaction proceeds fast with that fraction. Second, activation is significantly faster than aminoacyl transfer (50.2 s^{-1} (WT); 46.6 s^{-1} (R721A); 40.3 s^{-1} (R964A); 14.4 s^{-1} (ΔINS) vs 1.2 s^{-1}, 0.0014 s^{-1}, 0.0001 s^{-1}, and 0.3 s^{-1}, respectively). Given that enzymes are preincubated with ATP and an amino acid, and that activation is fast, the majority of enzymes (being in significant surplus anyway) should be in AA-AMP-bound form. Finally, if the two enzyme fractions are present in solution, one could observe two distinct kinetic phases and fit the curve to a double exponential.

*Given that our curves fit well to a monoexponential equation (see below and **newly added Supplementary Fig. 13**) and that the activation step is much faster than the transfer step, we can say with high confidence that the measured rate is not influenced by the activation-step chemistry. However, we were concerned that it could be influenced by tRNA binding (given the large K_M effect) and thus tested the transfer at twice the tRNA concentration. We did not observe an increase in rate (**newly added Supplementary Fig. 13**), confirming that what is measured is indeed the aminoacyl transfer chemistry.*

11. Line 202 S731 is mentioned in the figure legend (line 202) but does not appear in Figure 2D. It might be S713.

We thank the reviewer for observing this error. We corrected it in the revised version.

12. Figure 4: Each data point should include error bars if the experiment was performed in triplicate. Also, data should be shown, at least in the supplementary information, for the doubling of the enzyme concentration.

We thank the reviewer for this remark and understand the reasons for the request. However, for the reasons described below, we believe Fig. 4, in its current form, is the most appropriate way to present these data. Therefore, we are inclined to leave Fig. 4 showing representative curves and the mean value ± SEM calculated from k_{trans} values obtained in independent experiments, and to provide all individual time-course traces with corresponding fits in the newly added ***Supplementary Fig. 13****.*

To account for the inherent variability of biological systems, independent replicates were intentionally performed using different enzymes and tRNA preparations and, in some cases, preparations derived from different bacterial clones after transformation. This approach ensures the measured kinetic parameters are robust across independently prepared samples. As a result, greater variability in the raw time-course data is expected, making it more appropriate to analyse each experiment independently rather than pool all data points into a single fit. As shown in ***Supplementary Fig. 13****, each individual dataset is well described by a monoexponential equation, as indicated by high R^2 and low Sy,x values. Importantly, despite the observed variability among replicates, the fitted k_{trans} values are highly consistent for the corresponding protein or tRNA. We updated the legend for Fig. 4 by adding "For each protein or tRNA, data from the three independent experiments were fitted individually (****Supplementary Fig. 13****). The resulting k_{trans} values were used to calculate the mean ± SEM, which are shown in this figure together with representative curves."*

We have also included the data with doubled tRNA concentration (colored red) in ***Supplementary Fig. 13****. Doubling the tRNA concentration did not affect k_{trans}, indicating that the chemical step, rather than the binding event, is captured by k_{trans}.*

During the revision of our data, we realised an unintentional error in k_{trans} for the R721 and R964A mutants, which we corrected in the ***updated Fig. 4****. Instead of $k_{obs} < 0.0001$ s^{-1} reported for both mutants, k_{obs} for R721A is 0.001 s^{-1} and for R964A is < 0.001 s^{-1}. The effect remains the same; the drop in k_{obs} is 10^3-fold (instead of 10^4) and is the same as for the tRNA U36C variant. The text is changed accordingly (line 344 and Fig 2 caption, line 357).*

Newly added Supplementary Fig. 13 *with caption:*

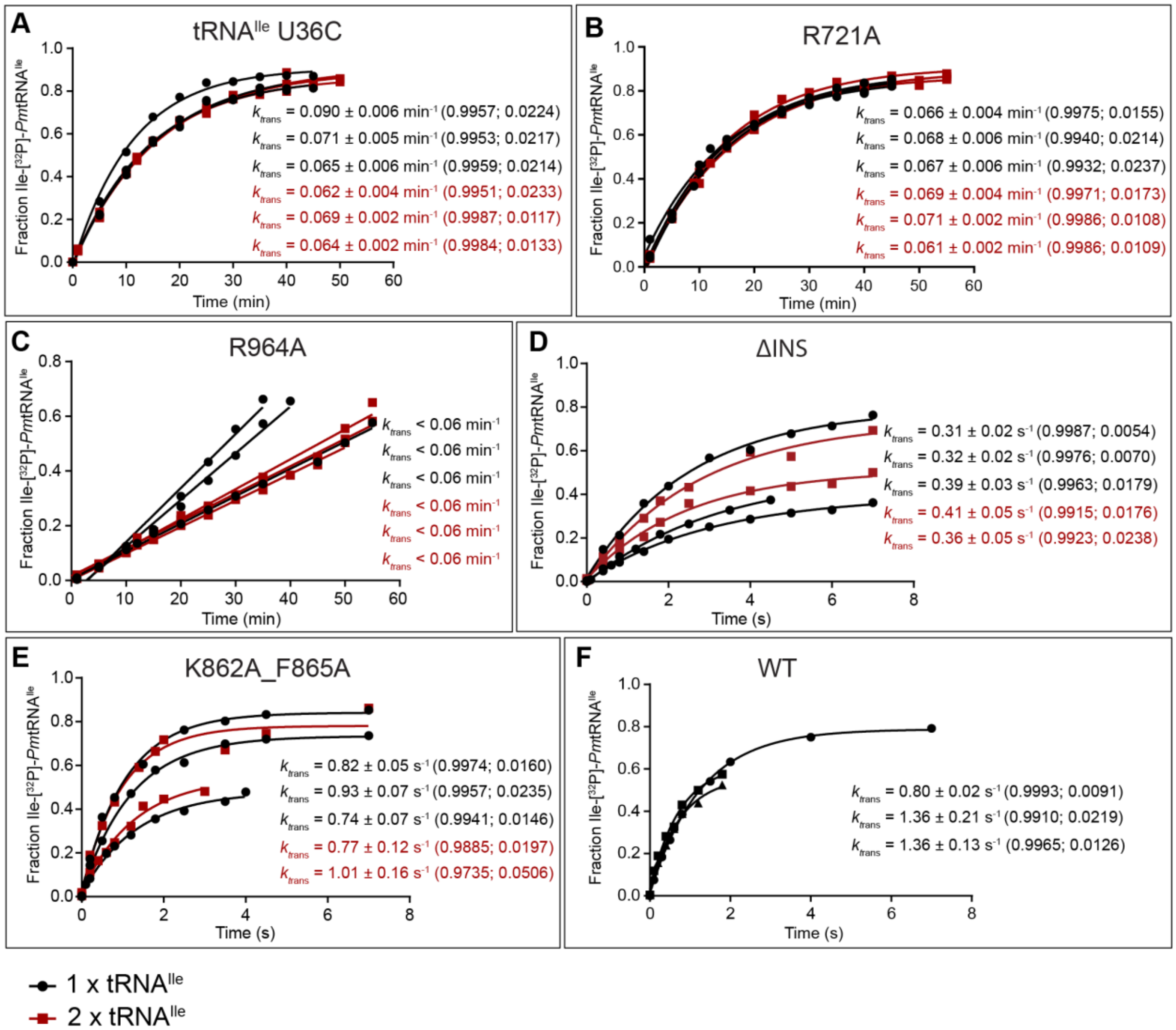

Supplementary Fig. 13: Time courses with fitted curves in single-turnover experiments. *For each protein and tRNA variant, three independent kinetic experiments were performed, and data were individually fitted to the equation* $y = Y_0 + A\,x\,(1 - e^{-k_{trans}\,x\,t})$. *Independent experiments were conducted using different protein or tRNA*^{Ile} *preparations and, in some cases, preparations derived from different bacterial clones after transformation. For each fit, the best-fit value of* k_{trans} ± *SE, along with the corresponding goodness-of-fit parameters (*R^2; $S_{y,x}$*), is reported. The data show that the experimental time courses are well described by the monoexponential curve, and that the independently determined* k_{trans} *values are highly consistent for each IleRS protein or tRNA*^{Ile}. *The resulting* k_{trans} *values were used to calculate the mean ± SEM shown in* ***Fig. 4*** *of the main text. The red-labelled timepoints and curves described experiments in which the concentration of tRNA*^{Ile} *was doubled while keeping the protein surplus, thereby maintaining single-turnover conditions. The same* k_{trans} *obtained at both tRNA*^{Ile} *concentrations shows that* k_{trans} *reflects the chemical step, not the binding step.*

During the revision of all data, we realised that in some cases we reported mean ± SEM obtained from fitting all data points collectively instead of the mean ± SEM calculated from values obtained by fitting individual replicates, which we consider the more appropriate approach for the reasons

*discussed above. Although the resulting values are very similar, for consistency we used the latter approach throughout the manuscript. Therefore, we updated the reported k_{cat}/K_M values for tRNAU20G in **Table 1** (0.045 ± 0.014 is replaced by 0.040 ± 0.009; 0.031 ± 0.005 is replaced by 0.030 ± 0.004, and 0.047 ± 0.002 is replaced by 0.053 ± 0.016) and data in **Supplementary Table 3** (we enclose original and revised version)*

Original

***Supplementary Table 3.** Steady-state kinetic parameters for isoleucine activation by wild-type and mutants PmIleRS2[a].*

		k_{cat} / s^{-1}	K_M (Ile) / μM	k_{cat} / K_M / s^{-1} $μM^{-1}$	Relative change[b] k_{cat} / K_M
	WT	50.2 ± 1.5	64.5 ± 6.7	0.78 ± 0.02	1
ARD1	R721A	46.6 ± 2.4	51.4 ± 9.3	0.88 ± 0.04	1.1
ARD3	R964A	40.3 ± 2.5	58.4 ± 12.4	0.7 ± 0.1	0.9
	ΔINS	14.4 ± 0.76	61.7 ± 11.3	0.24 ± 0.02	0.3

[a] Activation was tested using the ATP-PPi exchange assay.

[b] Relative change was defined as the ratio of mutants to wild-type enzyme PmIleRS2.

Values represent mean ± SEM of at least three independent experiments.

Revised

***Supplementary Table 3.** Steady-state kinetic parameters for isoleucine activation by wild-type and mutants PmIleRS2[a].*

		k_{cat} / s^{-1}	K_M (Ile) / μM	k_{cat} / K_M / s^{-1} $μM^{-1}$	Relative change[b] k_{cat} / K_M
	WT	50.3 ± 1.9	64.8 ± 3.4	0.78 ± 0.02	1
ARD1	R721A	47.4 ± 3.5	54.6 ± 5.8	0.88 ± 0.04	1.1
ARD3	R964A	40.6 ± 3.9	61.7 ± 13.6	0.70 ± 0.12	0.9
	ΔINS	13.9 ± 0.88	62.4 ± 1.3	0.23 ± 0.01	0.3

[a] Activation was tested using the ATP-PPi exchange assay.

[b] Relative change was defined as the ratio of mutants to wild-type enzyme PmIleRS2.

Values represent mean ± SEM of at least three independent experiments. Data from each experiment were fitted individually, and the resulting values were used to calculate the mean ± SEM.

Reviewer #3 (Remarks to the Author):

It is shown that, although IleRS enzymes have traditionally been subdivided into two groups based on their C-terminal domains, they are better described as having split into three evolutionary clades. In addition to bacterial IleRS1 and archaeal/bacterial/eukaryotic IleRS2, it is proposed a new clade of deep-branching archaeal IleRS that they dubbed IleRS-A.

Their phylogenetic analysis indicates that IleRS1 diverged from IleRS-A, likely driven by a replacement of the C-terminal domain. They also found evidence for ongoing C-terminal remodelling, with a concerted loss of the INS subdomain across the prokaryotic tree of life.
To better understand the role of ARD3 and INS, and the forces shaping their evolution, authors solved the crystal structure of P. megaterium IleRS2 in complex with tRNA and performed a detailed kinetic characterisation of ARD3, INS, and tRNA mutants. They found that P. megaterium IleRS2 recognises the anticodon bases with slightly higher fidelity than P. megaterium IleRS1. It is also shown that INS, which is present exclusively in the ARD2 domain of IleRS2 and IleRS-A, first served as a tRNA-binding domain in archaea and bacteria but later evolved into a tRNA recognition element in eukaryotes.
In summary, frame the replacement of ARD3 by ZBD to form the IleRS1 clade and the ongoing loss of INS as prokaryotic strategies for increased aminoacylation turnover. However, deeper phylogenetic analysis revealed that some archaeal IleRSs, so far attributed to the IleRS2 clade, actually cluster more closely with IleRS1. This observation raised the question of whether C-terminal domain types correspond to a clade separation. To clarify the evolutionary history of IleRS, a database of 96,219 IleRS sequences spanning bacteria, archaea, and eukaryotes and annotated their protein domain architectures was built.
They found that the standard two-group division scheme does not conform to the clade structure of the phylogenetic tree. In addition to IleRS1 and IleRS2, authors identified a novel clade of archaeal IleRS (IleRS-A). This clade has a C-terminal domain organisation like IleRS2 but has a HUP domain that is more closely related to that of IleRS1 (HUP1).
IleRS-A has a HUP domain that is more closely related to that of IleRS1 (termed HUP-1) but shares a type 2 C-terminal domain organisation with IleRS2, which comprises ARD1-ARD3 and optionally an ~80 amino acid insertion within the ARD2 domain (INS). In contrast, IleRS1 has a treble clef zinc-binding domain (ZBD) and lacks INS, which is referred to as the type 1 C-terminal organization.
They also determined the first crystal structure of an IleRS that contains the more evolutionarily unstable INS domain, making it a particularly useful point of comparison with the tRNA-bound structure of yeast IleRS2 17 and the tRNA- and mupirocin-bound structure of Staphylococcus aureus IleRS1 (SaIleRS1).

I highlight the result that IleRS split into three clades based on the HUP domain and C-terminus.
The present manuscript is a comprehensive analysis that renders a novel view of IleRS phylogeny, showing that the early archaeal split gave rise to the IleRS-A and IleRS2 lineages, followed by the subsequent emergence of IleRS1 within the IleRS-A branch. The deep-rooted position of IleRS-A strongly suggests that its HUP-1 domain more closely resembles the ancestral IleRS synthetic domain.

MAJOR
The scientific methods are rigorous, the objectives are clear, it is well explained and justified, the experimental procedures are modern and sound, the experimental design is appropriate, the statistical analyses are sound, in each step controls are wisely chosen, and the results are at the cutting edge and interesting. Figures are of high quality and well explained.

Maybe the authors would like to add a comment about ARSs research has recently expanded far beyond their canonical roles in translation to emerge as central players in signaling, human disease, and therapeutic interventions.

We thank the reviewer for this remark and have introduced the following in the discussion (lines 524-527).
AARSs have been recognised for important moonlighting roles outside translation[43,44]. Although we cannot rule out the possibility that ZBD contributes to non-canonical AARS functions, our data demonstrate its distinct features in canonical aminoacylation.

I think that this manuscript is appropriate for Nature Communications.

Minor
Lines 155-156:
"Thus, current evidence supports a model in which IleRS evolution involved two independent passages from archaeal to bacterial to eukaryotic forms."

Outstanding and outlandish. Eukarya diverged from Archaea (Lochiarcheota). Maybe a diagram can be useful.

*We thank the reviewer for raising this point, and we are glad to clarify our meaning. We should emphasise that this sentence refers only to the evolution of IleRS itself, not to speciation events. Understood in that sense, the statement is directly supported by the tree: the eukaryotic IleRS clades nest within bacterial sequences, and the bacterial sequences nest within archaeal sequences (**Fig. 1**).*

While this is a surprising result, it reflects the extent to which the IleRS tree records the successive takeover of new IleRS forms over time rather than the history of the organisms carrying them. As Woese noted, the AARS enzymes are fundamentally modular in their action, and therefore readily transferred and swapped as functional units. For this reason, their gene trees track enzyme replacement rather than species divergence — making them precisely the enzymes one would not want to use to infer species-level evolutionary events.

*To make it clearer, we provide a **new Supplementary Fig 5 (**given in the response to Q8 of the reviwer#1) describing a proposed evolutionary scenario for IleRS and updated the sentence, which now reads "Thus, current evidence supports a model (**Supplementary Fig. 5**) in which IleRS evolution involved two independent passages from archaeal to bacterial to eukaryotic parent organisms." (lines 197-198)*

Line 362: Replace "EcIleRS1" by E. coli IleRS1.
We replaced EcIleRS1" by E. coli IleRS1 (line 448)

Lines 410-411: "…bacteria encode two tRNA isoacceptors"

We added $tRNA^{Ile}$ (line 509)

Reference 4: Giegé.
Reference 15: Mycobacterium tuberculosis
Reference 16: Insights into Editing from an Ile-tRNA Synthetase Structure with tRNAIle and Mupirocin.
Reference 19: Söll, D. & Söll, S.
Reference 20: Methanobacterium thermoautotrophicum
Reference 25: Escherichia coli
Reference 26: Escherichia coli
Reference 29: Pseudomonas fluorescens
Reference 30: Staphylococcus aureus
Reference 31: Escherichia coli
Reference 35: Eldred, E.W. & Schimmel, P.R.
Reference 39: Escherichia coli
Reference 40, 42, and 47: tRNAIle

We updated the references as suggested by the reviewer and also identified and corrected several additional errors in the cited references in the revised version.
