# Supplementary material for: C-terminal evolutionary remodelling of isoleucyl-tRNA synthetases is a prokaryote-specific strategy for tuning aminoacylation rate
Source: Nat Commun. 2026 Oct 12;17:11575. doi: 10.1038/s41467-026-78241-1 (PMC13752280; doi:10.1038/s41467-026-78241-1)
Supplement: Supplementary file 3 [file 41467_2026_78241_MOESM3_ESM.pdf]

Supplementary information

C-terminal evolutionary remodelling of isoleucyl-tRNA synthetases is a prokaryote-specific strategy for tuning aminoacylation rate

Petra Modrusan[1], Alojzije Brkic[1], Andre Lecona Buttelli[2], Marc Leibundgut [3], Igor Zivkovic[1], Nenad Ban[3]*, Liam M. Longo[2,4]*, Ita Gruic-Sovulj[1]*

[1]University of Zagreb Faculty of Science, Department of Chemistry, Zagreb, Croatia

[2]Earth-Life Science Institute, Institute of Science Tokyo, Tokyo, Japan

[3] Department of Biology, Institute of Molecular Biology and Biophysics, ETH Zürich, Zürich, Switzerland

[4]Blue Marble Space, Seattle, USA

*Corresponding authors

Correspondence to:

Ita Gruic-Sovulj, University of Zagreb Faculty of Science, Department of Chemistry, Horvatovac 102A, 10000 Zagreb, Croatia, gruic@chem.pmf.hr

Liam M. Longo, Earth-Life Science Institute, Institute of Science Tokyo, 2-12-1-IE-1 Ookayama, Meguro, 152-8550 Tokyo, Japan, llongo@elsi.jp

Nenad Ban, Department of Biology, Institute of Molecular Biology and Biophysics, ETH Zürich, 8093 Zürich, Switzerland, ban@mol.biol.ethz.ch

Supplementary Figure 1. Validation and performance of IleRS hidden Markov models (HMMs).

Supplementary Figure 2. Outgroup selection.

Supplementary Figure 3. Maximum-likelihood phylogenies of individual IleRS domains.

Supplementary Figure 4. Tolerance to the GIHH motif and sensitivity to mupirocin by archaeal enzymes.

Supplementary Figure 5. Proposed model of IleRS evolution.

Supplementary Figure 6. Unbiased electron density maps are used to build and confirm the presence of bound tRNA.

Supplementary Figure 7. Direct experimental evidence for the binding of the INS subdomain to the elbow region of the $tRNA^{Ile}$ in the crystal structure.

Supplementary Figure 8. Architecture of the *Pm*IleRS2:$tRNA^{Ile}$:ATP complex and comparison with the structures of eukaryotic IleRS2 and bacterial IleRS1 enzymes bound to tRNAs.

Supplementary Figure 9. Structural rearrangements occur in *Pm*IleRS2 upon $tRNA^{Ile}$ binding.

Supplementary Figure 10. Local domain rearrangements between the four *Pm*IleRS2:$tRNA^{Ile}$:ATP complexes in the asymmetric unit of the crystal.

Supplementary Figure 11. B-factor distribution in the four *Pm*IleRS2:$tRNA^{Ile}$:ATP complexes as arranged within the asymmetric unit or as a side-by-side comparison.

Supplementary Figure 12. Yeast IleRS2 exhibits more stringent anticodon recognition than *S. aureus* IleRS1 and contacts the D-loop by base-specific interactions.

Supplementary Figure 13. Time courses with fitted curves in single-turnover experiments.

Supplementary Figure 14. Structural differences in $tRNA^{Ile}$ recognition between *Pm*IleRS2 and *Sa*IleRS1.

Supplementary Figure 15. Truncation of the INS subdomain does not perturb the overall *Pm*IleRS2 fold.

Supplementary Figure 16. Complementation of mupirocin-inhibited *E. coli* growth by *Pm*IleRS2 variants at various IPTG-induced expression levels.

Supplementary Table 1. Summary of data collection and refinement statistics of the determined structure.

Supplementary Table 2. Comparison of *Pm*IleRS1 and *Pm*IleRS2 aminoacylation rates toward $tRNA^{Ile}$ GAU and its variants.

Supplementary Table 3. Steady-state kinetic parameters for isoleucine activation by wild-type and mutant *Pm*IleRS2 enzymes.

Supplementary Table 4. Primers used to design *Pm*IleRS2 and *Pm*$tRNA^{Ile}$ GAU mutants.

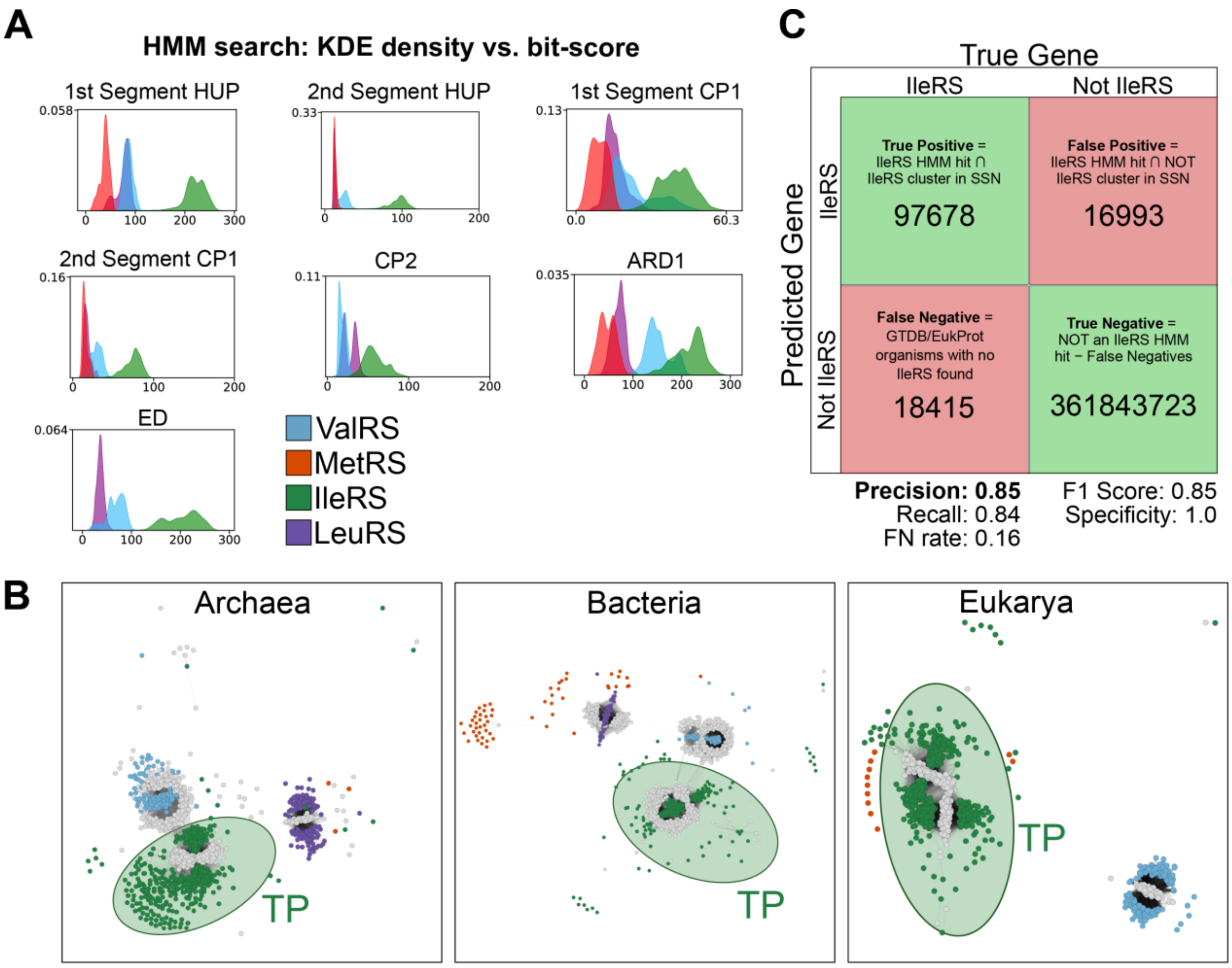

Supplementary Fig. 1: Validation and performance of IleRS hidden Markov models (HMMs). (A) Distribution of normalised domain alignment scores for IleRS, ValRS, LeuRS, and MetRS families, illustrating the discrimination capacity of the HMMs. Each panel corresponds to one IleRS domain HMM, and colour coding reflects the aminoacyl-tRNA synthetase (AARS) family as indicated in the legend. **(B)** Sequence similarity networks (SSNs) of putative IleRS, validated IleRS, and other class I AARS sequences. Nodes represent clustered protein sequences, and edges indicate significant sequence similarity (see Methods). Node colours follow the scheme in (A). Sequences collected from GTDB and EukProt are rendered as grey nodes. Edge shade indicates bit scores; darker edges represent higher similarity and lighter edges represent lower similarity. **(C)** Confusion matrix summarising the classification performance of the IleRS HMMs against the GTDB and EukProt databases. True positives (TP) represent sequences clustering with known IleRS genes. False positives correspond to sequences assigned to non-IleRS AARS clusters (ValRS, LeuRS, or MetRS). False negatives denote organisms in which no IleRS genes were detected. This is likely to be an overestimate because genome incompleteness can result in the absence of an IleRS gene. True negatives represent all genes searched that did not return an IleRS hit, minus the estimated false negative count. The raw data used for HMM construction and validation, SSN construction and

filtering, and a curated set of 97,678 full-length IleRS protein sequences have been deposited in the Zenodo repository under DOI: 10.5281/zenodo.21453230[1].

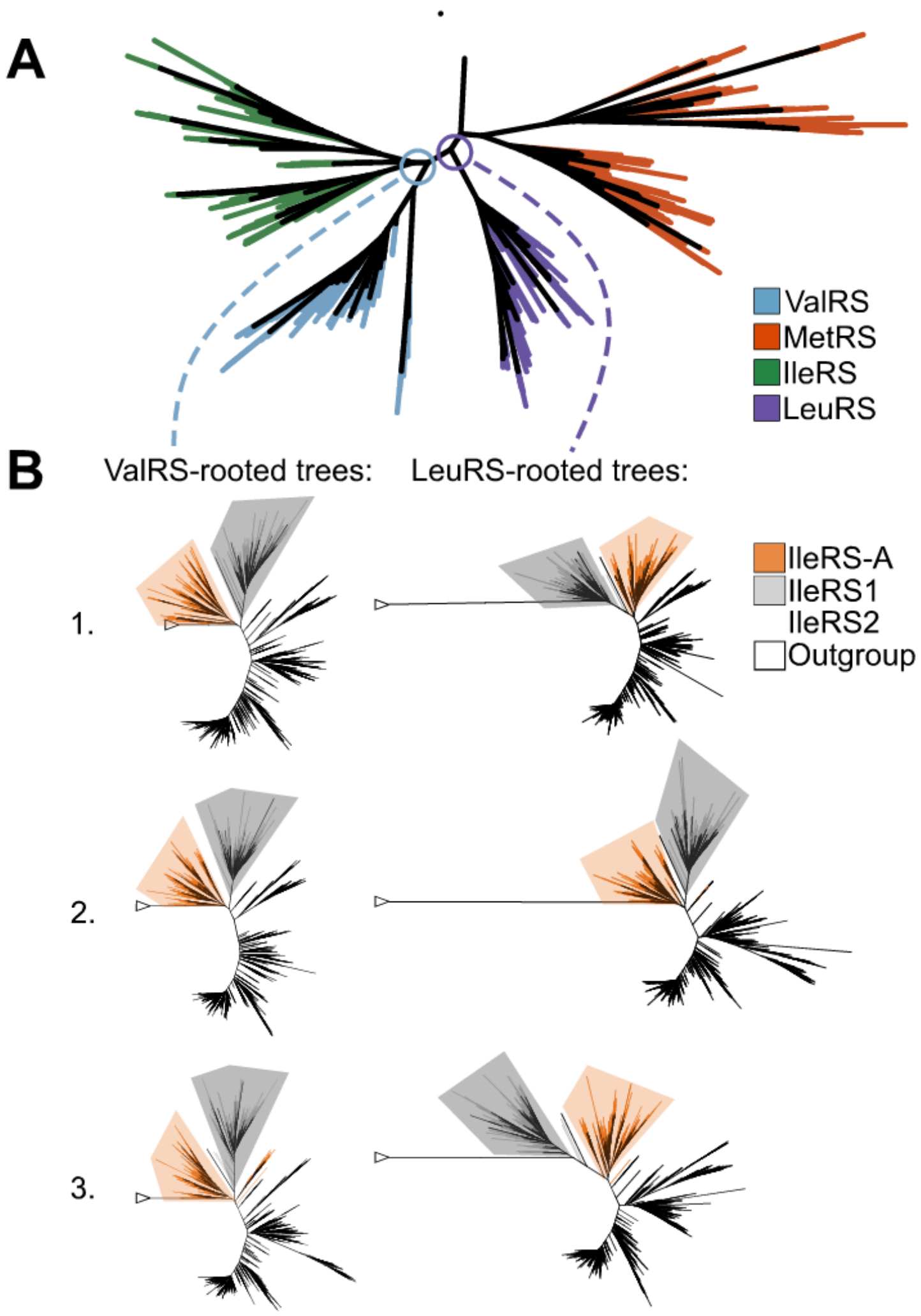

Supplementary Fig. 2: Outgroup selection. (A) Phylogenetic tree of full-length sequences of MetRS, LeuRS, ValRS, IleRS, CysRS (not shown), and ArgRS (not shown). MetRS, LeuRS, ValRS, IleRS form a cohesive clade, where IleRS is more closely related to ValRS than to LeuRS. For each AARS family, sequences were retrieved from EggNOG v5.0 and clustered to 50% identity. One representative sequence from the top 100 largest clusters within that family was gathered. Sequence alignment, trimming, and tree building were performed as described in the Methods section. **(B)** The top three trees of the IleRS synthetic domain were assessed by log-likelihood using ValRS (left) or LeuRS (right) as the outgroup. Sequence alignment, extraction of the synthetic domain, and tree building was performed as described in the main text. Note that while ValRS rooting consistently identifies IleRS-A as the primal clade, rooting with LeuRS is less stable, positioning the root in either IleRS1 or IleRS-A, both of which belong to the HUP-1 clade. AARS and IleRS phylogenetic trees, including the underlying sequence sets and alignments used to build them, have been deposited in the Zenodo repository under DOI: 10.5281/zenodo.21453230[1]

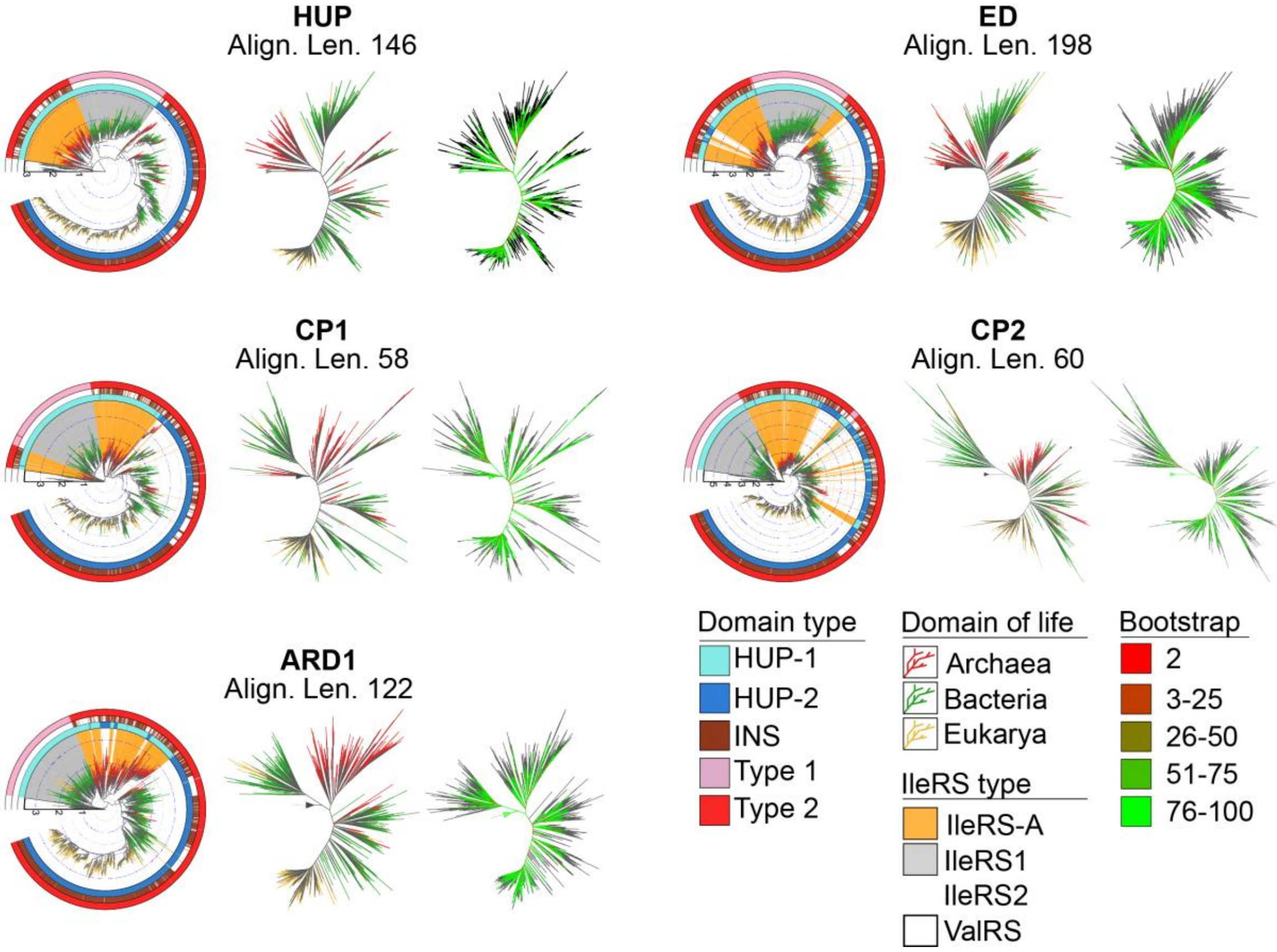

Supplementary Fig. 3: Maximum-likelihood phylogenies of individual IleRS domains. Maximum-likelihood trees are shown for the catalytic (HUP), editing (ED), connective peptide 1 (CP1), connective peptide 2 (CP2), and anticodon recognition 1 (ARD1) domains, each rooted using the homologous ValRS domain. For each domain, three representations are provided (left to right): a rooted tree with annotated outer rings, an unrooted topology, and an unrooted tree coloured by bootstrap support. In the rooted representations, the concentric outer rings denote (from inner to outer): the HUP domain type, the presence of the INS insertion, and the C-terminal domain type. Branch colours indicate the domain of life (Archaea, Bacteria, Eukarya), with bootstrap values displayed using the colour scale shown. Alignment lengths are indicated above each panel. Across the HUP, ED, and CP1 trees, the ValRS-outgroup-inferred root consistently falls within archaeal IleRS-A sequences, whereas in CP2 and ARD1, the ValRS-outgroup-inferred root is positioned near IleRS-A but between the IleRS1 and IleRS-A clades. In all trees, IleRS-A sequences (characterised by HUP-1 and C-terminal type 2) form a coherent and well-supported cluster, even though neither HUP nor C-terminal sequence information was included in these domain-specific alignments (with the exception of the HUP tree). Domain-level phylogenetic trees, including the underlying sequence sets and alignments used to build them, have been deposited in the Zenodo repository under DOI: 10.5281/zenodo.21453230[1].

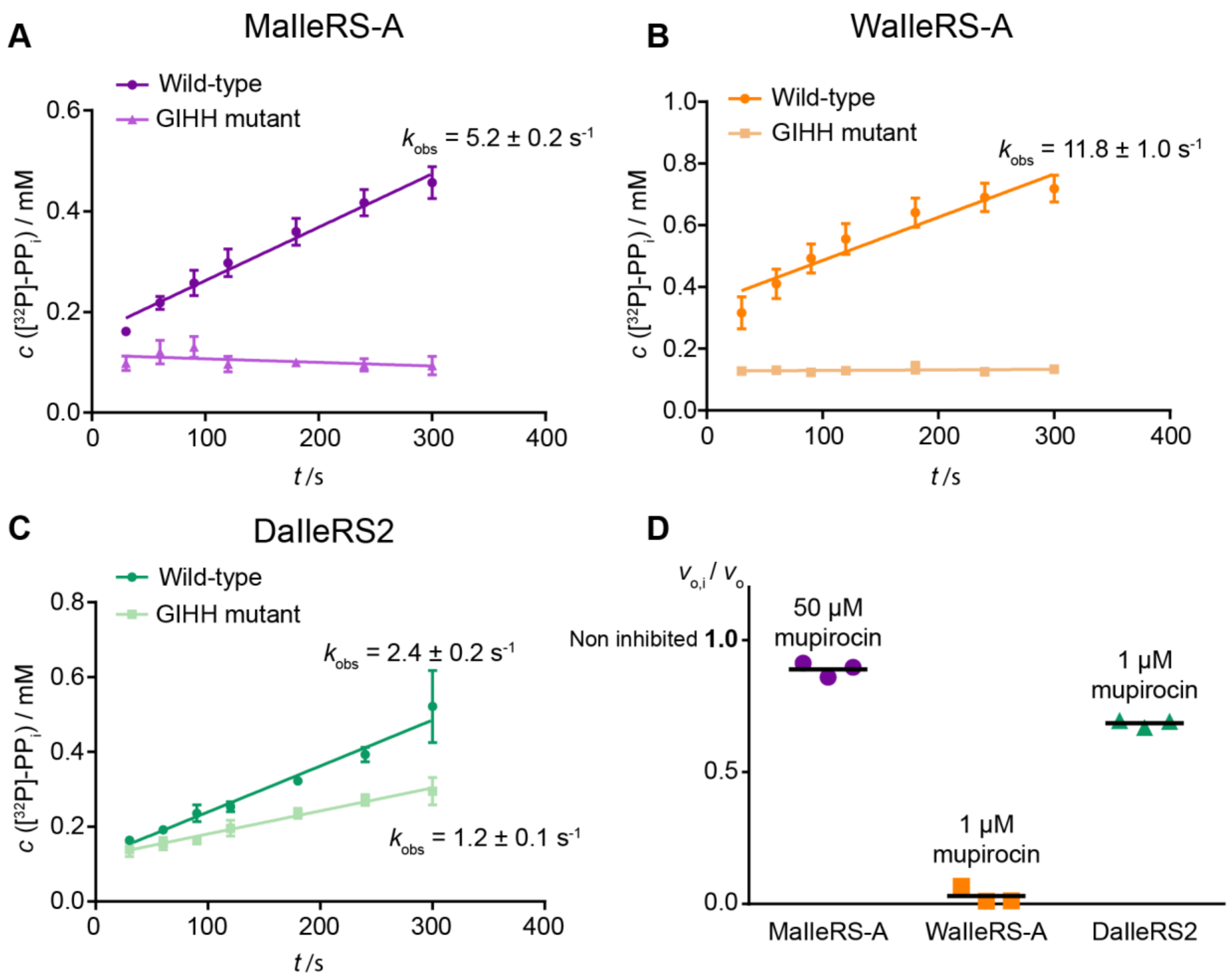

Supplementary Fig. 4: Tolerance to the GIHH motif and sensitivity to mupirocin by archaeal enzymes. (A-C) The introduction of the GIHH motif inactivated IleRS-A from Woesearchaeota archaeon (WaIleRS-A) and *Candidatus* Micrarchaeota archaeon (MaIleRS-A), while IleRS2 from *Candidatus* Diapherotrites archaeon (DaIleRS2) remained highly active. GIHH represents the non-canonical motif in which the 1st and 3rd positions are swapped. Due to sequence variability of the HIGH motif, the corresponding non-canonical motif sequences in these enzymes are GIHT (WaIleRS-A), GVHT (MaIleRS-A), and GAHN (DaIleRS2). For simplicity, we collectively refer to these motif variants as the GIHH throughout the manuscript. Wild-type (WT) and GIHH mutant variants are shown in dark and light shades, respectively: purple for MaIleRS-A, orange for WaIleRS-A, and green for DaIleRS2. **(D)** Inhibition was tested at 1 μM mupirocin. At that value, WaIleRS-A was almost completely inhibited, showing sensitivity to mupirocin similar to IleRS1 enzymes, which have K_i values in the nM or even pM range[2–5]. As expected, DaIleRS2 was not significantly inhibited, consistent with the K_i for IleRS2 enzymes being in the μM range[5–7]. Surprisingly, MaIleRS-A was not inhibited even with 50 μM mupirocin. The rates in (A-D) were obtained by measuring [^{32}P]ATP-PP_i exchange, a standard assay for testing the amino acid activation step, *i.e.*, the first step of aminoacylation[8]. The ATP concentration was 4 mM, and the Ile concentration was 10 x K_M (Ile) for the

corresponding enzyme when testing WT and GIHH mutant activity in (A-C) or 1 x K_M (Ile) when testing mupirocin inhibition (D). v_0 and $v_{0,i}$ are the initial velocities in the absence and presence of mupirocin, respectively. $v_{0,i}/v_0$ is the fractional activity in the presence of mupirocin at the stated concentration. The data represent three (n=3) independent experiments. For panels (A–C), each experiment was fitted individually, and the resulting k_{obs} values were used to calculate the mean ± SEM shown in the figure. For visualisation of the error bars, data points from all three experiments were fitted together. For panel (D), fractional activity was determined for each independent experiment, and the mean value is indicated by the horizontal line. Source data are provided as a Source Data file.

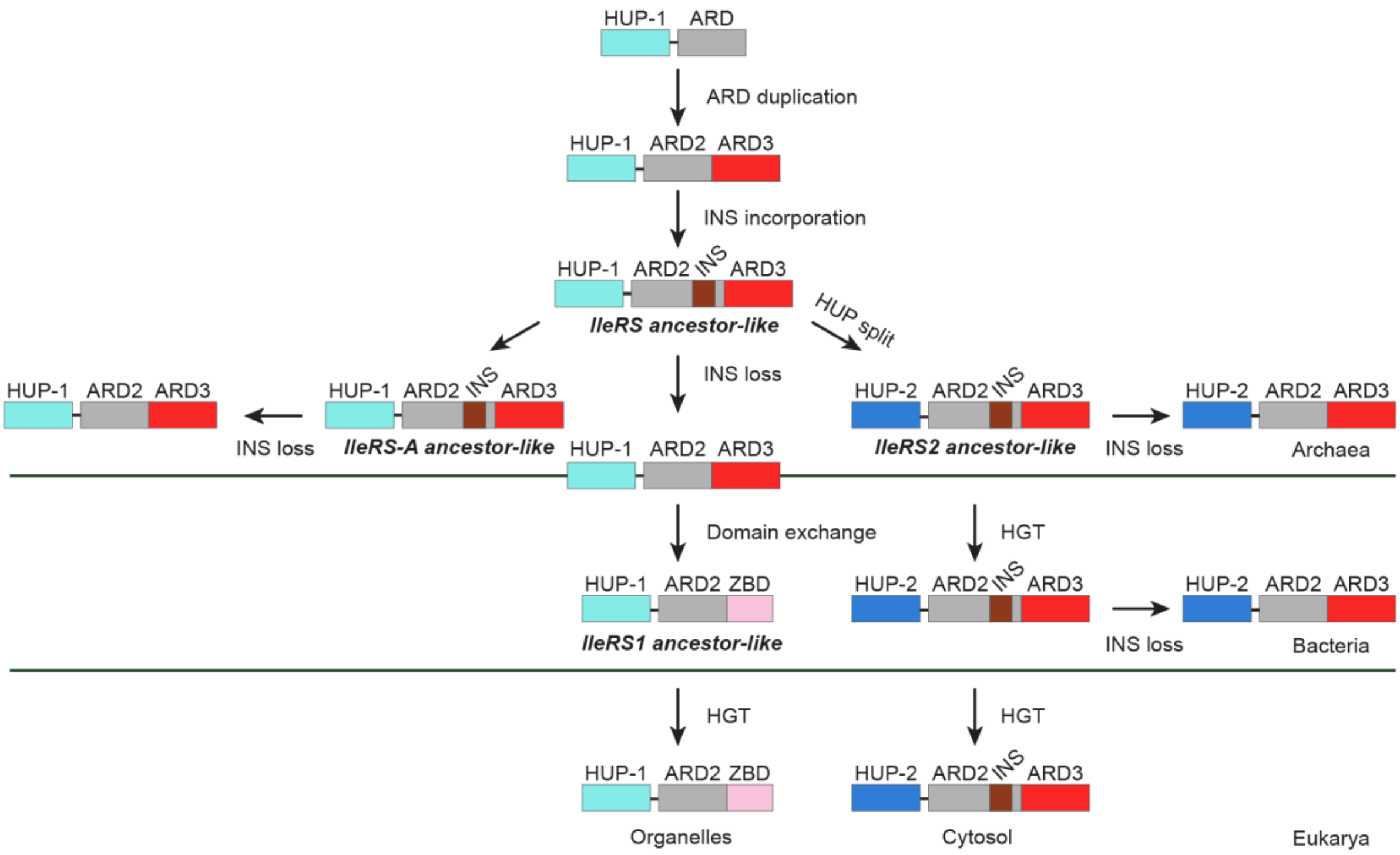

Supplementary Fig. 5: Proposed model of IleRS evolution. Based on the ValRS-rooted tree, we hypothesise that a HUP-1 domain served as the last common synthetic domain of IleRS. As ARD2 and ARD3 are homologous domains, ARD duplication was the likely first event of C-terminal remodelling. The next stage of C-terminal domain remodelling occurred with the accretion of the INS subdomain within ARD2. We deem the presence of INS in the ARD ancestor domain to be less likely because it would imply that (a) ARD3 had completely lost its copy of the INS subdomain prior to the IleRS ancestor, as no known ARD3 domain has an INS insertion, thereby necessitating an additional event of structural remodelling, and (b) both ARD2 and ARD3 underwent INS loss in nearly equivalent ways, despite the two parent domains playing different structural roles. The presence of INS in the IleRS ancestor is supported by the fact that the majority of contemporary domains within the IleRS-A clade contain the INS subdomain, including the deepest rooting branches. Subsequent transitions – such as the exchange of ARD3 with ZBD in the emergence of IleRS1 – are implied by the phylogenetic tree of the synthetic domain presented in Fig. 1. At this resolution, we cannot state whether the loss of the INS domain during IleRS1 evolution occurred in archaea or bacteria. HGT stands for horizontal gene transfer. Domains and colouring scheme: HUP-1 (cyan), HUP-2 (blue), ARD2 (anticodon recognition domain 2; grey), INS (insertion into ARD2; brown), ARD3 (anticodon recognition domain 3; red) and ZBD (zinc-binding domain; light pink). The remaining domains (editing (ED), connective peptides 1 and 2 (CP1, CP2) and ARD1 (anticodon recognition domain 1), whose evolution is not addressed in this model, are

represented by a line connecting HUP (catalytic domain) and ARD (ancestral anticodon recognition domain).

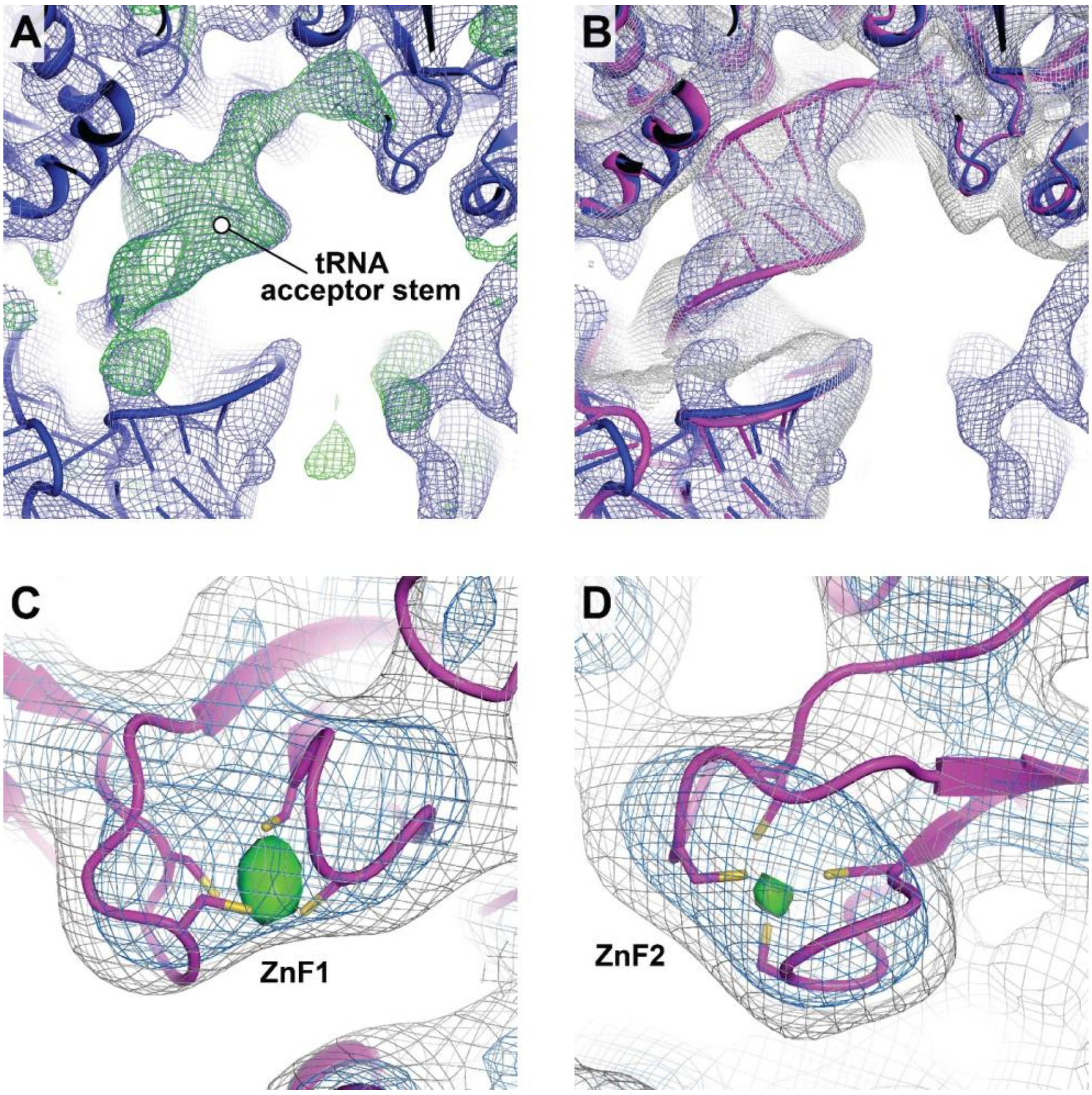

Supplementary Fig. 6: Unbiased electron density maps are used to build and confirm the presence of bound tRNA. (A) The initial molecular replacement model (blue cartoon), with parts of the tRNA acceptor stem omitted, showed strong unbiased positive mF_o–DF_c Fourier difference density for the missing tRNA segments (green mesh, contoured at 2 σ) after domain-wise rigid body refinement. The corresponding 2mF_o–DF_c Fourier difference map (blue mesh) is contoured at 0.7 σ. **(B)** Completed model of the acceptor stem of the tRNA molecule. The 2mF_o–DF_c Fourier difference map shown in panel (A) was subjected to 4-fold real-space non-crystallographic symmetry (NCS) averaging around the displayed reference molecule, allowing modelling of the missing $tRNA^{Ile}$ residues (magenta) into the averaged map (grey). **(C)** Calculation of the anomalous difference Fourier map (green isosurface, contoured at 4.8 σ) revealed the positions of the zinc ions in the two zinc fingers (zinc ions are coordinated by C181, C184, C387, and C390 in ZnF1 and C459, C462, C500, and C503 in ZnF2, respectively). The height of the anomalous peaks was used to monitor the progression and convergence of the building and refinement procedure, resulting in considerable improvement of R_{work} and R_{free}. Additionally, an unbiased simulated annealing composite OMIT map is shown at 0.8 σ and 1.8 σ contour levels (grey and blue meshes, respectively).

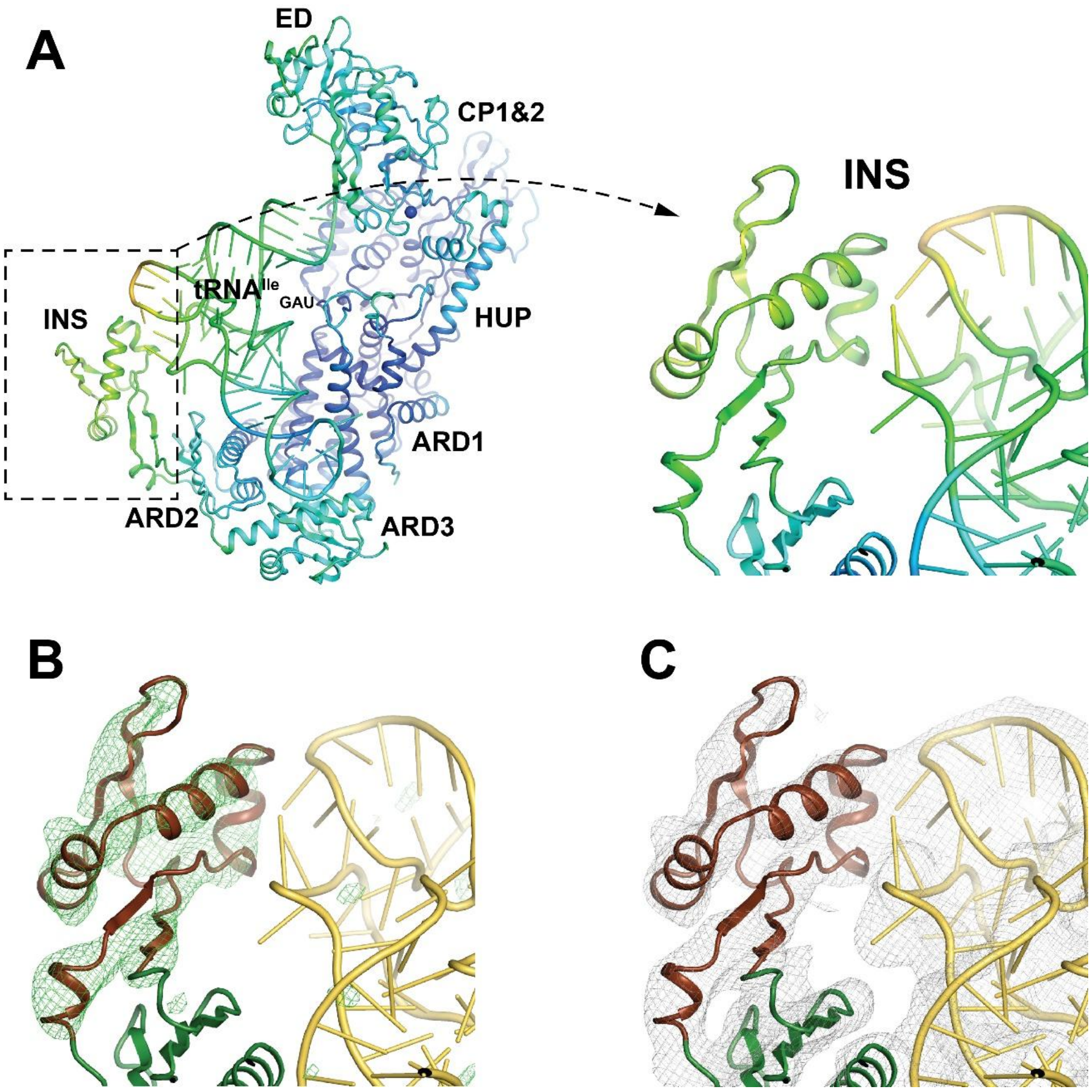

Supplementary Fig. 7: Direct experimental evidence for the binding of the INS subdomain to the elbow region of the tRNA^{Ile} in the crystal structure. **(A)** Representative B-factor distribution in the *Pm*IleRS2:tRNA^{Ile}:ATP complex (exemplified here by chains A,a in the asymmetric unit (ASU)), indicating that the INS subdomain (inset) and tRNA^{Ile} elbow regions are more flexible than the remaining parts of the complex. The structure is coloured according to B-factor, with blue indicating lower and red indicating higher values. The B-factor distribution of all chains in the asymmetric unit is visualised in Supplementary Fig. 11. **(B)** The INS subdomain is clearly recognisable in an unbiased mF_o–DF_c OMIT map (green mesh, contoured at 3.0 σ), allowing precise rigid body docking with only minor positional errors. The map was obtained by omission of the INS subdomain from the model, random shift of all remaining atoms by 0.25 Å and resetting B-factors to remove model bias, followed by refinement to convergence. **(C)** The INS subdomain is clearly visible in the 2mF_o–DF_c Fourier difference map, although at a lower contour level than the rest of the molecule (here shown at 0.5 σ as a grey mesh, contoured 4.6 Å around the model). HUP is the catalytic domain, ED is

the editing domain, CP1&2 are connective peptides 1 and 2, ARD1-3 are anticodon recognition domains (1-3), and INS is the insertion in ARD2.

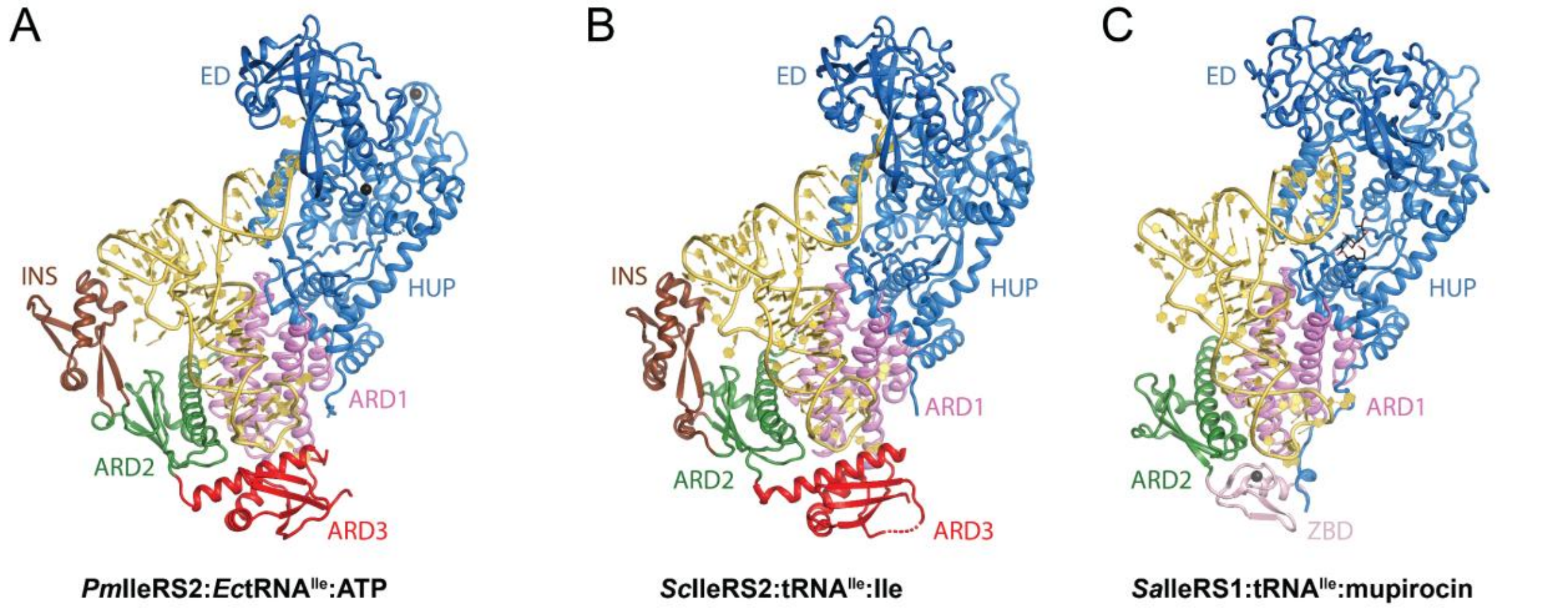

Supplementary Fig. 8: Architecture of the *Pm*IleRS2:tRNA^{Ile}:ATP complex and comparison with the structures of eukaryotic IleRS2 and bacterial IleRS1 enzymes bound to tRNAs. (A) The *Pm*IleRS2:tRNA^{Ile}:ATP complex features a conserved HUP domain, two connective peptides (CP) hosting the editing domain (ED), and the tRNA anticodon binding domains: ARD1 (violet), ARD2 (green), and ARD3 (red). Insertion into ARD2 (INS, brown) interacts with the tRNA D-loop. The acceptor stem is oriented toward the editing site, while the anticodon loop is positioned within a pocket formed by the ARD1 and ARD3 domains. Because only weak residual mF_o–DF_c Fourier difference density was observed for the bound ATP cofactors, the ligand was not modelled. **(B)** The *S. cerevisiae* IleRS2:tRNA^{Ile}:Ile complex (PDB: 8WND)[9] displays an overall architecture analogous to that of *Pm*IleRS2. **(C)** In contrast, the *S. aureus* IleRS1:tRNA^{Ile}:mupirocin complex (PDB: 1FFY)[10] lacks INS and features a zinc-binding domain (ZBD, light pink) at the C-terminus.

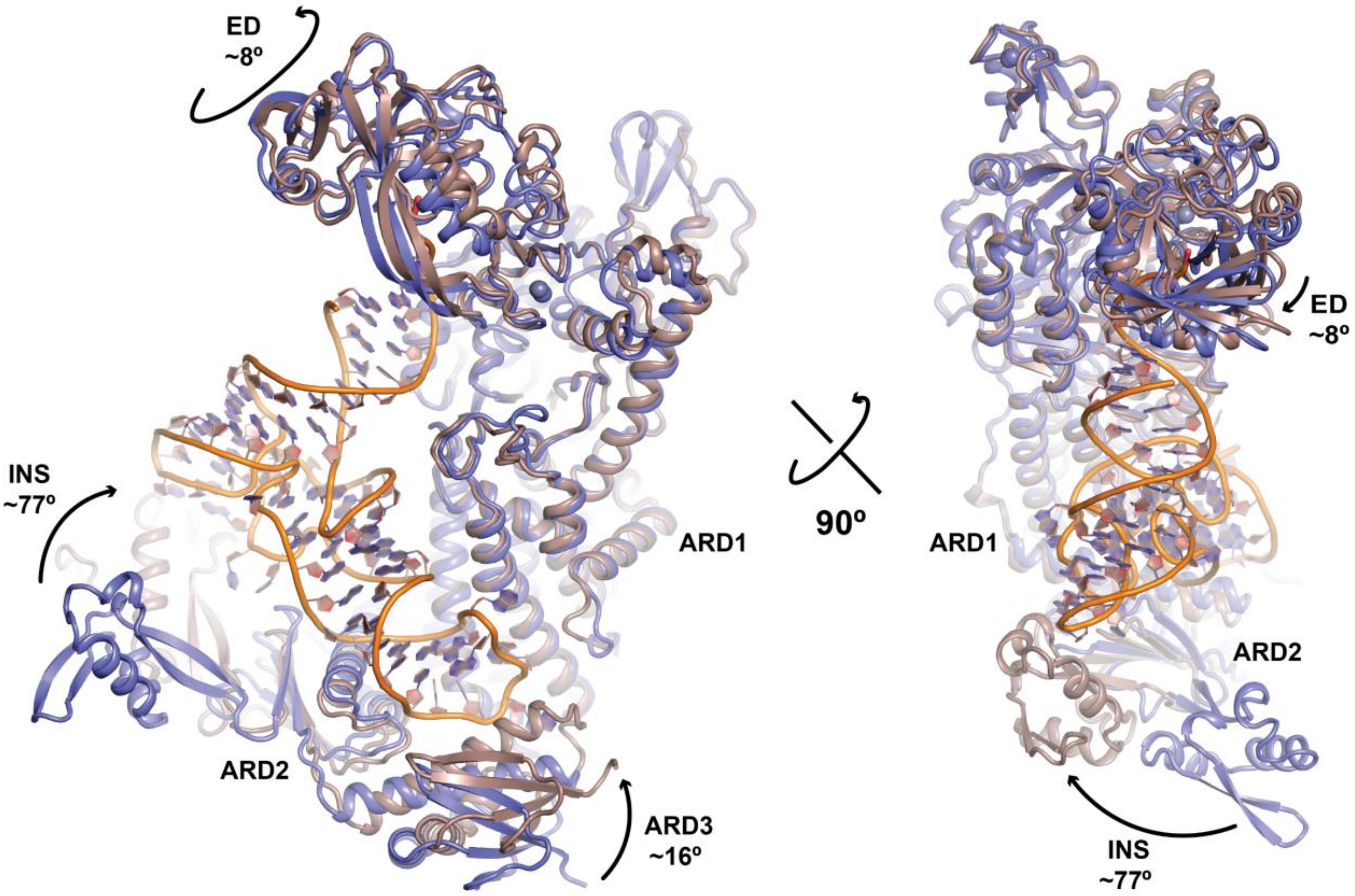

Supplementary Fig. 9: Structural rearrangements occur in *Pm*IleRS2 upon tRNA^{Ile} binding. Superposition of the *Pm*IleRS2:tRNA^{Ile}:ATP complex (brown) with the *Pm*IleRS2:Ile-AMS complex (PDB: 8C8V)[6] (blue), shown in orientations perpendicular to the longest (left) and shortest (right) axes of the complex. The alignment reveals steric rearrangements of *Pm*IleRS2 domains upon tRNA^{Ile} binding. This rearrangement involves repositioning of the editing domain and closure of the subdomains within the C-terminal anticodon-binding domain toward the tRNA. Notably, INS establishes contacts with the D-loop of the tRNA^{Ile}. ED is the editing domain, ARD1-3 are anticodon recognition domains (1-3), and INS is the insertion in ARD2.

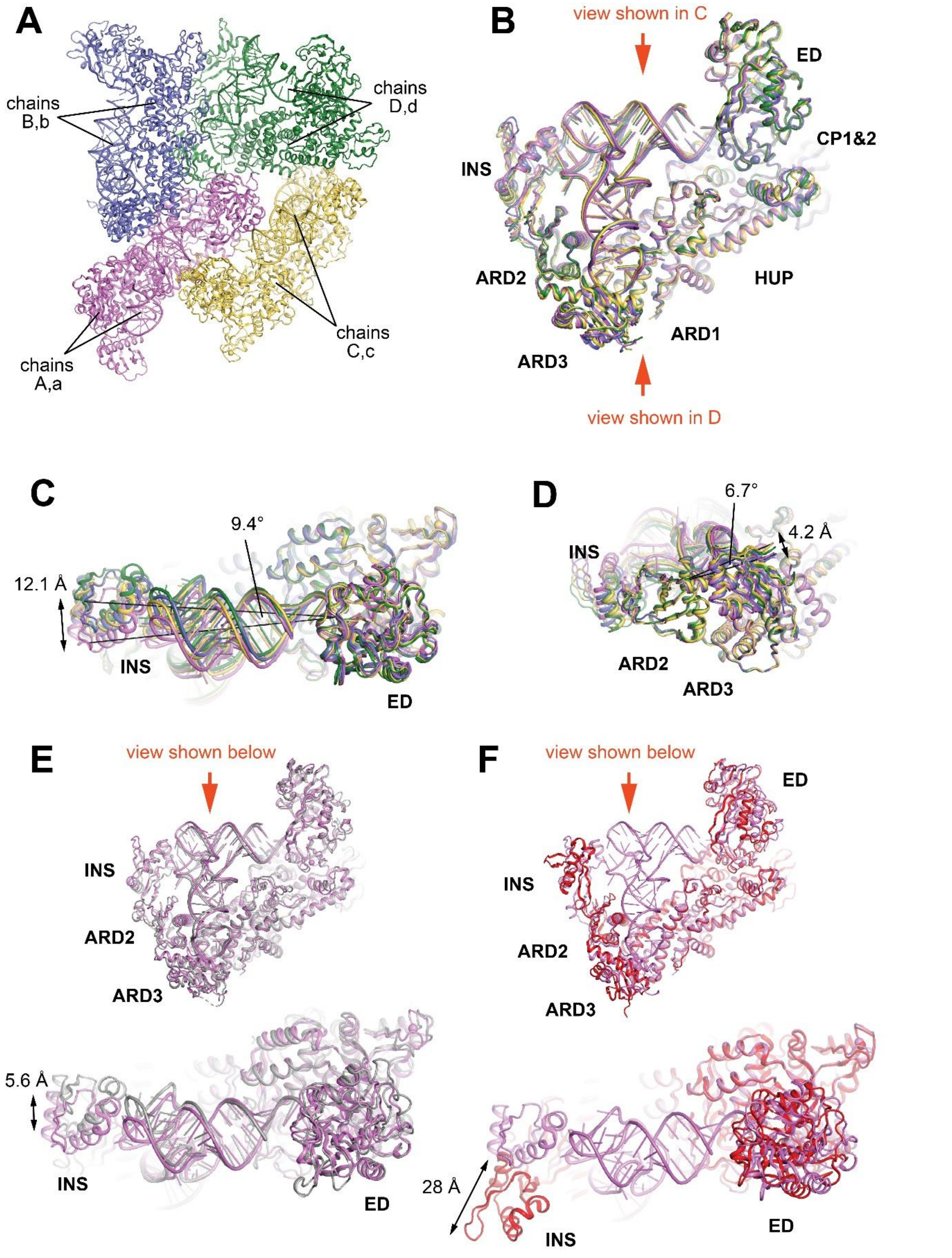

Supplementary Fig. 10: Local domain rearrangements between the four *Pm*IleRS2:tRNA^{Ile}:ATP complexes in the asymmetric unit (ASU) of the crystal. A comparative analysis of *Pm*IleRS2 domain rearrangements within and between the four copies in the ASU revealed only moderate conformational differences, indicating that these are likely

induced by crystal packing and do not affect the overall architecture of the IleRS2:tRNA^{Ile}:ATP complexes. This suggests intrinsic flexibility rather than distinct functional states. **(A)** Localisation of the four IleRS2:tRNA^{Ile}:ATP complexes in the ASU. **(B)** In the side view of the four superimposed complexes, the domains are very similarly arranged. **(C)** The top view reveals a swinging motion of the INS subdomain in concert with the bound tRNA elbow around a hinge point localised at the CCA-3' end of the tRNA. **(D)** The bottom view shows slight repositioning of the ARD3 domain. **(E)** Superposition of the yeast structure (grey, PDB 8WND[9], chains A,T) onto chains A,a (magenta) reveals a conformational change in a similar range. **(F)** Compared to the tRNA-free structure (red, PDB 8C8V[6]), the repositioning of the INS domain is more dramatic, suggesting a flexibly attached INS domain that becomes positioned in a specific manner only in the presence of tRNA^{Ile}. HUP is the catalytic domain, ED is the editing domain, CP1&2 are connective peptides 1 and 2, ARD1-3 are anticodon recognition domains (1-3), and INS is the insertion in ARD2.

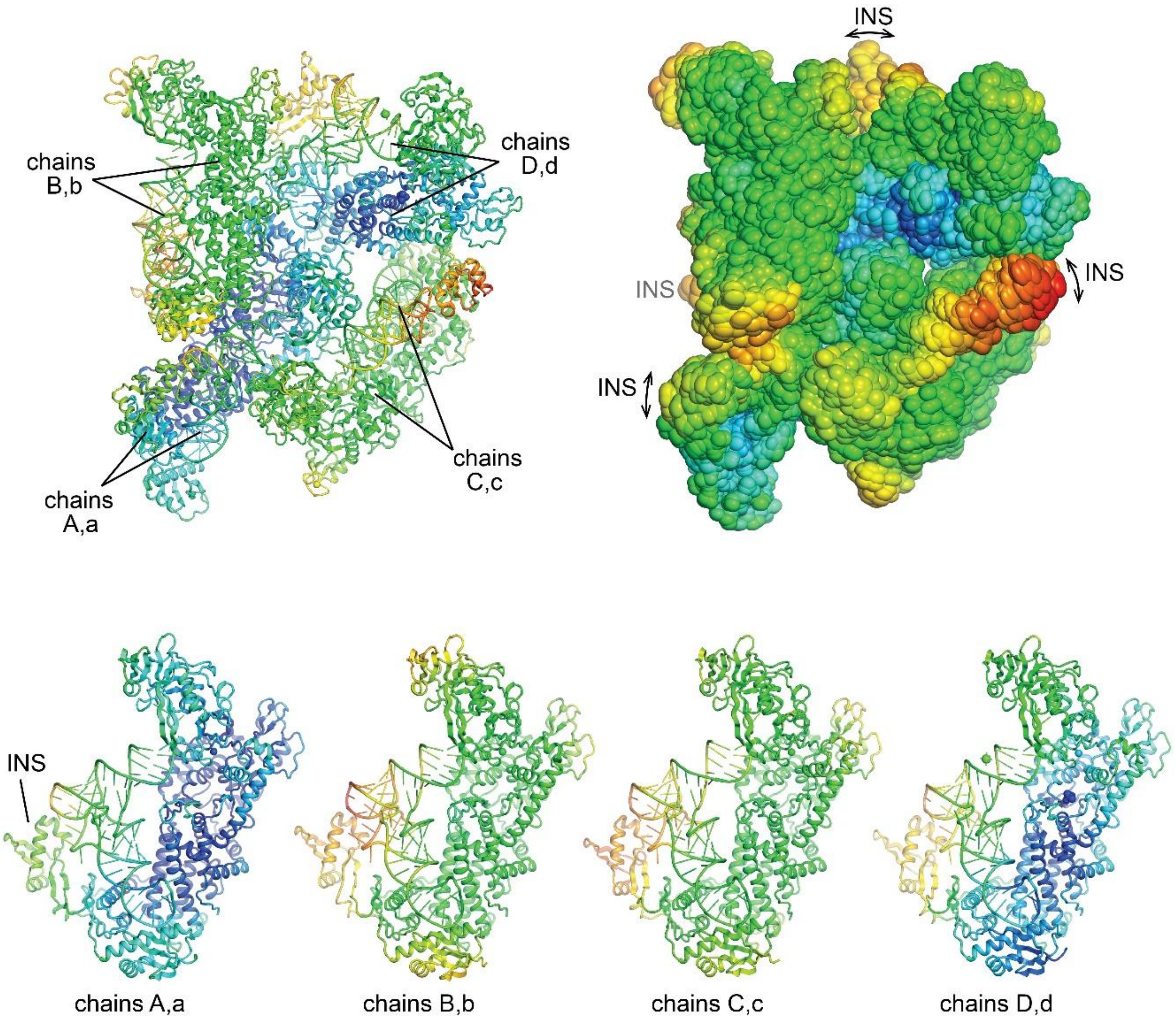

Supplementary Fig. 11: B-factor distribution in the four *Pm*IleRS2:tRNA^{Ile}:ATP complexes as arranged within the asymmetric unit or as a side-by-side comparison. The INS subdomain bound to the tRNA^{Ile} elbow region represents the most flexible part, reflecting the conformational heterogeneity in that area when the four complexes are superimposed. The directional mobility can be recognised in the ellipsoidal anisotropic B-factor representation obtained by TLS (translation-rotation-screw-rotation) refinement. Structures are coloured according to B-factor, with blue indicating lower and red indicating higher values.

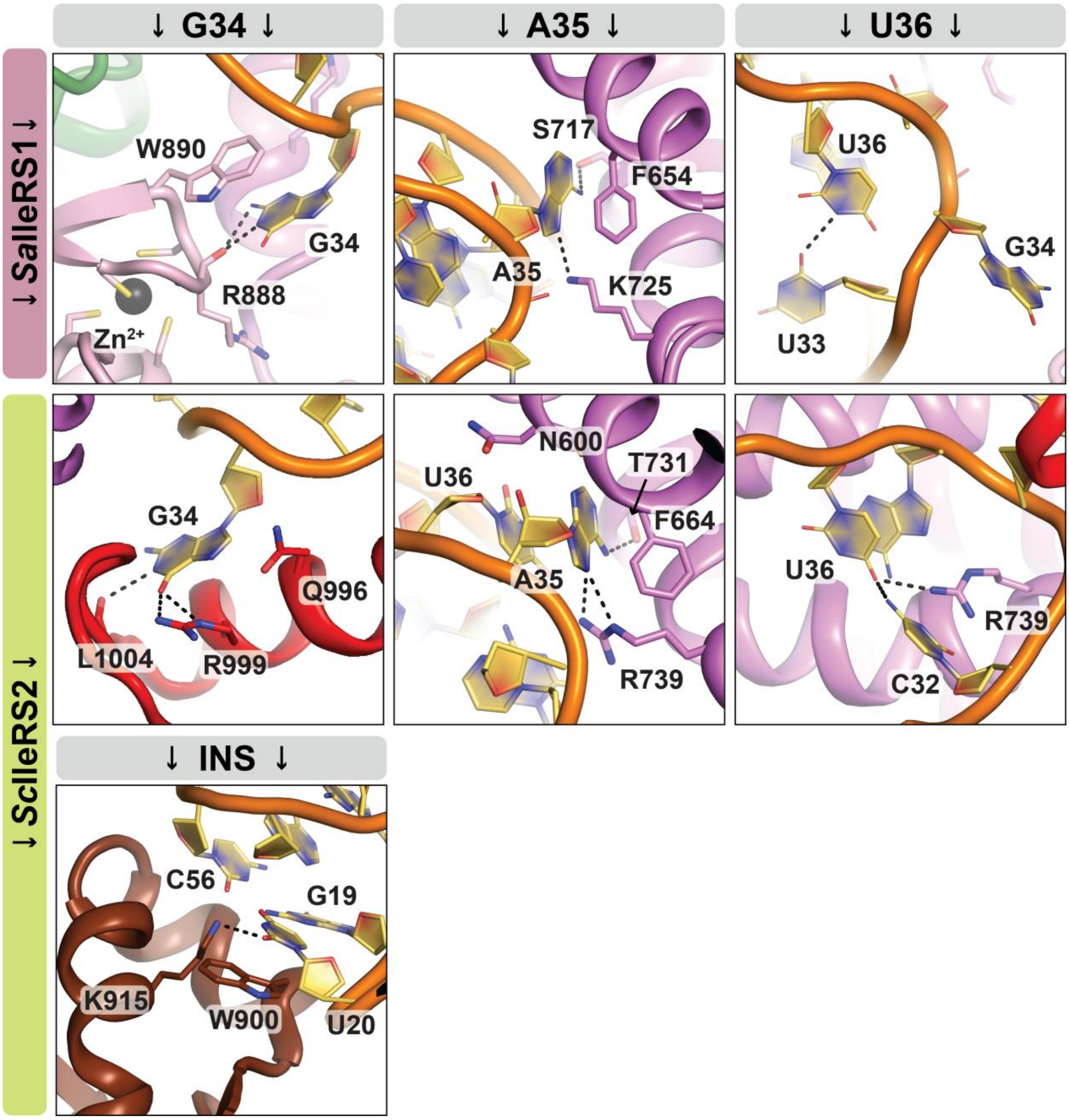

Supplementary Fig. 12: Yeast IleRS2 exhibits more stringent anticodon recognition than *S. aureus* IleRS1 and contacts the D-loop by base-specific interactions. Recognition of the three $tRNA^{Ile}$ anticodon bases (G34, A35, U36) by *Sa*IleRS1 (PDB: 1FFY)[10] and *Sc*IleRS2 (PDB: 8WND)[9] is shown in the upper and middle panels. The lower panels depict recognition of the $tRNA^{Ile}$ D-loop by *Sc*IleRS2 (PDB: 8WND)[9].

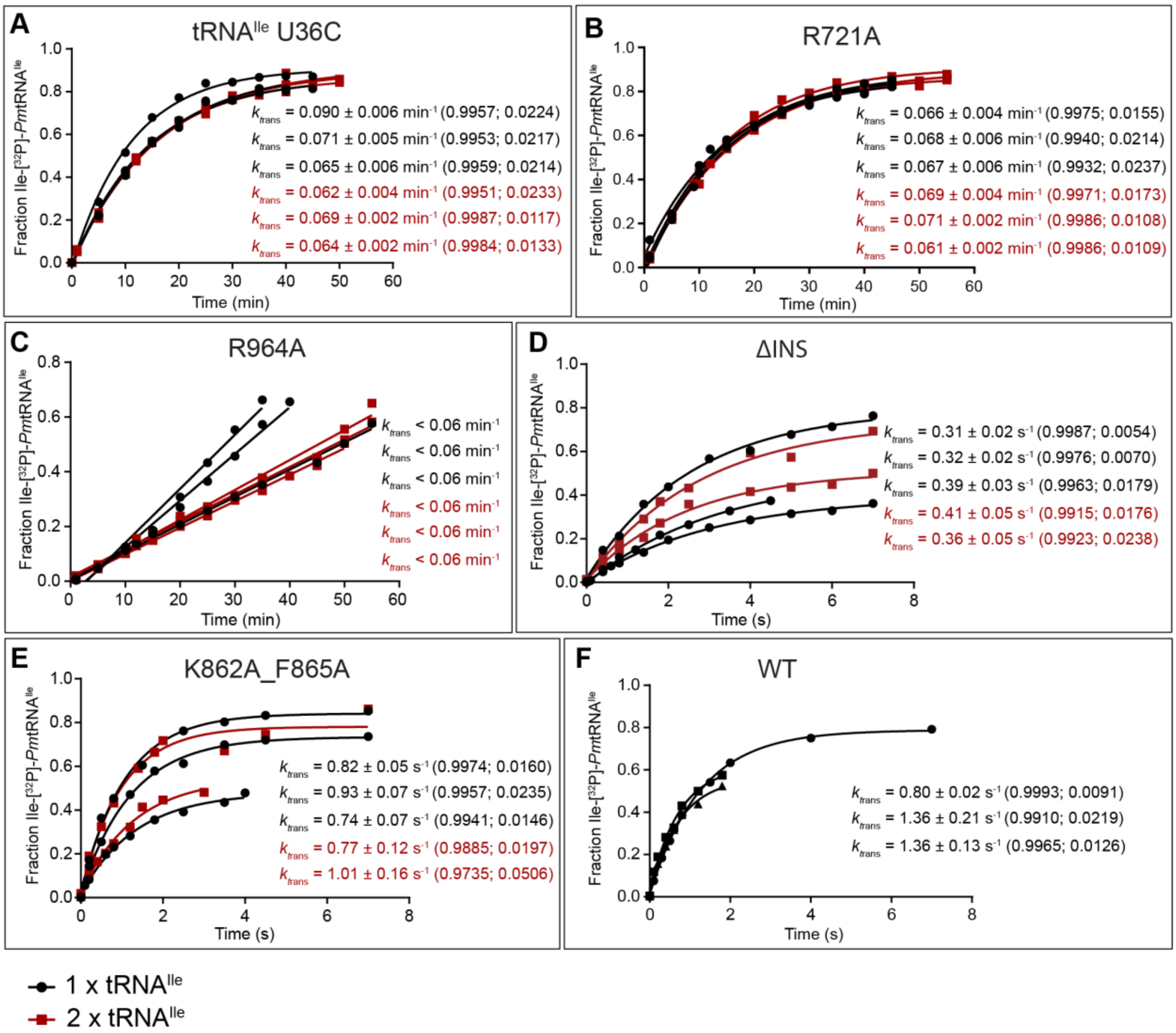

Supplementary Fig. 13: Time courses with fitted curves in single-turnover experiments. For each protein and tRNA variant, three (n=3) independent kinetic experiments were performed, and data were individually fitted to the equation $y = Y_0 + A \times (1 - e^{-k_{\text{trans}} \times t})$. Independent experiments were conducted using different protein or $tRNA^{Ile}$ preparations and, in some cases, preparations derived from different bacterial clones after transformation. For each fit, the best-fit value of k_{trans} ± SE, along with the corresponding goodness-of-fit parameters (R^2; $S_{y,x}$), is reported. The data show that the experimental time courses are well described by the monoexponential curve, and that the independently determined k_{trans} values are highly consistent for each IleRS protein or $tRNA^{Ile}$. The resulting k_{trans} values were used to calculate the mean ± SEM shown in Fig. 4 of the main text. The red-labelled timepoints and curves describe experiments in which the concentration of $tRNA^{Ile}$ was doubled while keeping the protein surplus, thereby maintaining single-turnover conditions. The same k_{trans} obtained at both $tRNA^{Ile}$ concentrations shows that k_{trans} reflects the chemical step, not the binding step. Source data are provided as a Source Data file.

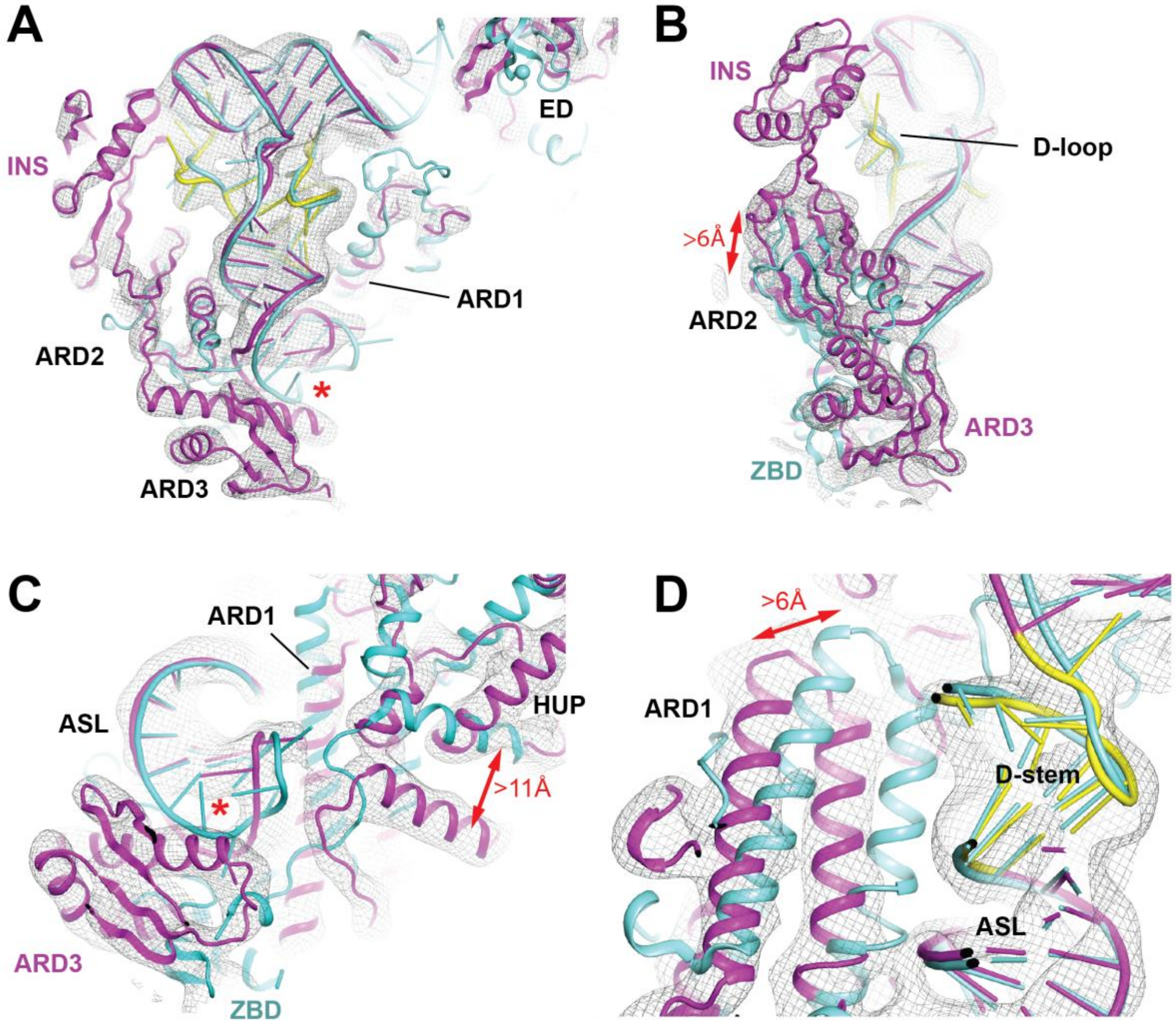

Supplementary Fig. 14: Structural differences in tRNA^{Ile} recognition between *Pm*IleRS2 and *Sa*IleRS1. (A, B) Different views showing the superimposed *Sa*IleRS1 (PDB: 1FFY[10], cyan) and *Pm*IleRS2 (magenta), with the 2mF_o–DF_c Fourier map (grey mesh) contoured at 1.0 σ and the tRNA^{Ile} D-stem and D-loop areas highlighted in yellow. The INS subdomain is present only in *Pm*IleRS2, where it binds the D-loop of tRNA^{Ile}, while ARD3 replaces the zinc-binding domain (ZBD) present in *Sa*IleRS1, with the ARD1 and ARD2 domains considerably shifted. As a reference for superposition, the tRNA acceptor arms were aligned. **(C)** The anticodon loop (red asterisk) adopts an alternative conformation when bound to *Pm*IleRS2 compared to *Sa*IleRS1, while the HUP and ARD1 domains are shifted upwards. **(D)** In *Pm*IleRS2, the ARD1 domain is detached from the D-stem of the tRNA^{Ile}, while in *Sa*IleRS1 it forms close contact**.** HUP is the catalytic domain, ED is the editing domain, ARD1-3 are anticodon recognition domains (1-3), and INS is the insertion in ARD2. ASL stands for tRNA anticodon stem-loop.

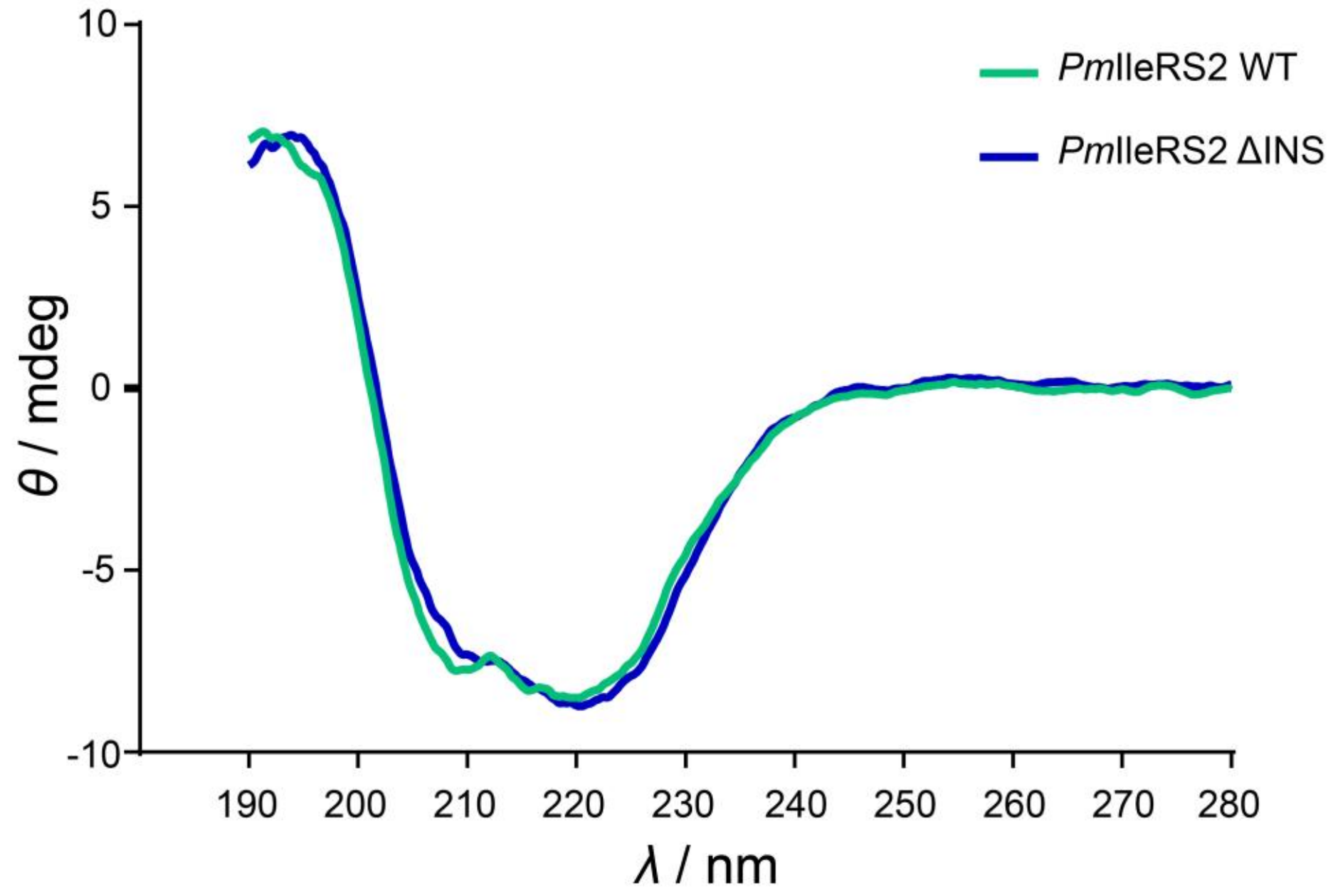

Supplementary Fig. 15: Truncation of the INS subdomain does not perturb the overall *Pm*IleRS2 fold. Circular dichroism (CD) spectrum of *Pm*IleRS2 WT (green) and ΔINS (blue) (1 mg mL^{-1}) in 1 mM NaH_2PO_4 pH 7.4 at 25 °C; l = 0.1 mm; V = 30 μL. CD spectroscopy did not reveal a structural perturbation related to the excision of INS.

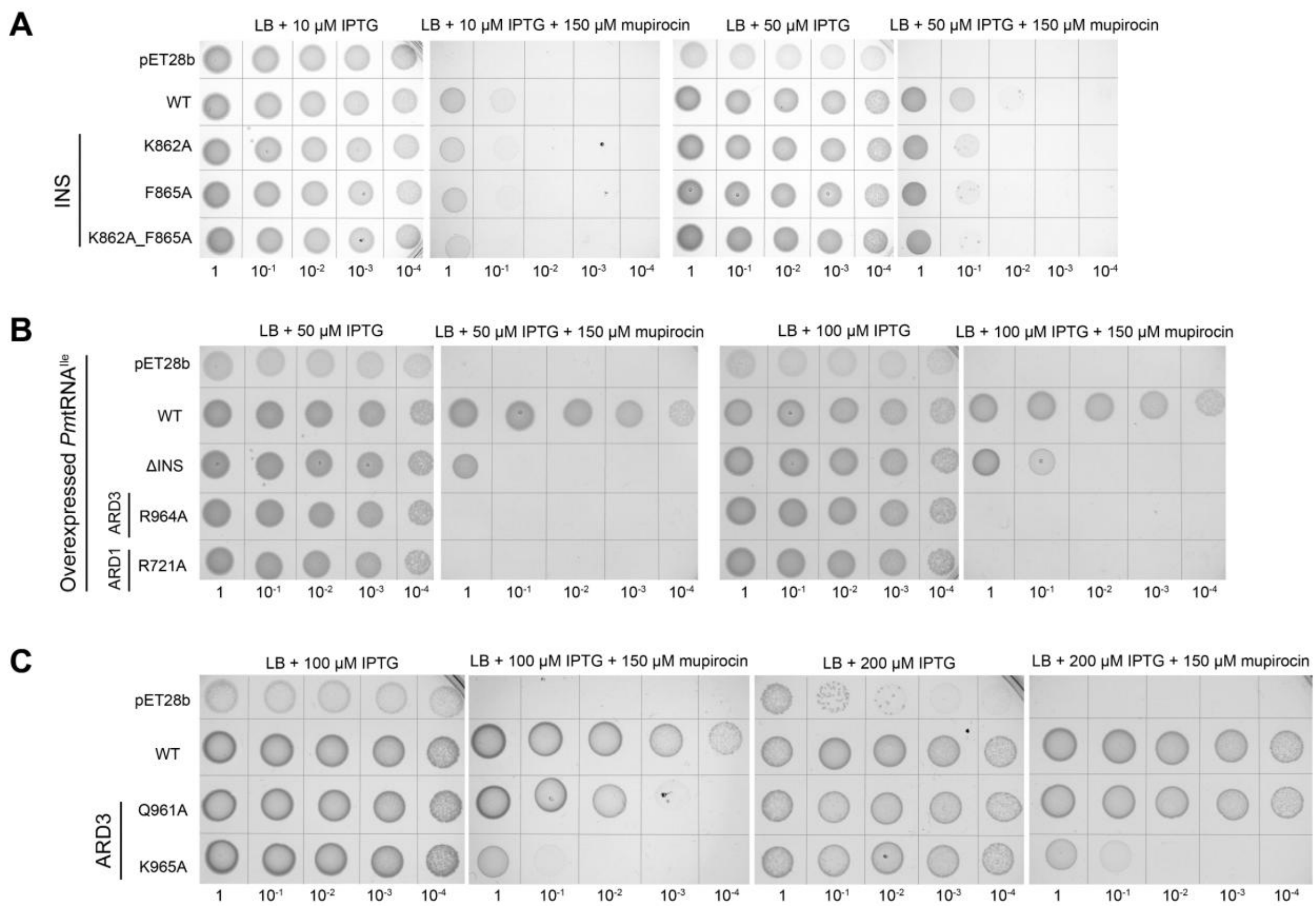

Supplementary Fig. 16: **Complementation of mupirocin-inhibited *E. coli* growth by *Pm*IleRS2 variants at various IPTG-induced expression levels.** Functionality of the *Pm*IleRS2 mutants *in vivo* was assessed by their ability to complement mupirocin-inhibited *E. coli* IleRS1. Expression of the mutants was induced from the pET28b plasmid using various isopropyl-β-D-1-thiogalactopyranoside (IPTG) concentrations. **A)** INS mutants rescue *E. coli* growth at all IPTG concentrations used. **B)** ΔINS fail to complement inactivated IleRS1 at 50 μM IPTG, while complementing it weakly at 100 μM IPTG, in both cases, *Pm*tRNA^{Ile} was co-overexpressed. Anticodon-recognising mutants R964A and R721A cannot complement *Ec*IleRS1 in any of these conditions. **C**) Q961A and K965A rescue *E. coli* growth at 100 μM and 200 μM IPTG. For each panel, growth without mupirocin is shown on the left, the negative control (empty plasmid with and without overexpressed tRNA^{Ile}) is positioned at the top, and the positive control (overexpression of the WT) is placed in the second row. The experiments were repeated at least twice.

Supplementary Table 1. Summary of data collection and refinement statistics of the determined structure.

**Complex**	***Pm*IleRS2:*Ec*tRNA^{Ile} GAU:ATP**
**PDB ID old / extended**	9U0O / pdb_00009U0O
**Data collection and processing** (XDS & XSCALE, BUILD=20220220)	
Wavelength (Å)	1.000
Resolution range*(Å)	48.51-5.30 (5.44-5.30)
Space group	$P2_12_12_1$
Unit cell dimensions	a=121.75 Å, b=147.01 Å, c=344.11 Å, α=β=γ=90°
Unique reflections*	23078 (1660)
Multiplicity	13.5
Completeness (%)	99.8 (99.6)
R-merge (%)*	10.5 (206.4)
I/σ(I)	13.37 (1.63)
$CC_{1/2}$	1.0 (0.77)
**Coordinate refinement** (PHENIX 1.21.2_5419)	
Reflections used in refinement	20481 (1271)
Completeness (%)	88.7 (77.0)
R_{work} / R_{free} (%)*	20.5/25.0 (36.8/44.3)
R_{free} test set size (%)	8.67
Model composition	
Number of copies / ASU	4
Non-hydrogen atoms	39,884
Protein residues	4128
RNA residues	304
Ligands: Zn^{2+} / SO_4 / Mg^{2+} x $6H_2O$	8 / 1 / 1
B-factor from Wilson plot (Å^2)	250
B-factors min/mean/max (Å^2)	
Overall	213/373/659
Protein	213/364/627
RNA	257/421/659
Ligands	247/351/480
**Model Validation** (MolProbity 4.5.2)	
General	
R.m.s. deviations	
Bond lengths (Å)	0.004
Bond angles (°)	0.72
Clashscore	5.7
Protein	
Rotamer outliers (%)	0.66
CaBLAM outliers (%)	2.0
Ramachandran plot	
Favored (%)	95.19
Allowed (%)	4.54
Disallowed (%)	0.27
RNA	
Pucker outliers (%)	0
Bond outliers (%)	0
Angle outliers (%)	0
Suite outliers (%)	16.8
(*) Values for highest resolution shell are indicated in parentheses	

Supplementary Table 2. Comparison of *Pm*IleRS1 and *Pm*IleRS2 aminoacylation rates toward $tRNA^{Ile}$ GAU and its variants[a,b].

	**Substrate**	**k_{obs} / s^{-1}**
*Pm*IleRS1	$tRNA^{Ile}$	0.41 ± 0.06
	$tRNA^{Ile}$ U20G	0.29 ± 0.04
	$tRNA^{Ile}$ U21G	0.30 ± 0.03
	$tRNA^{Ile}$ G19C	0.31 ± 0.03
	$tRNA^{Ile}$ G34C	0.026 ± 0.003
	$tRNA^{Ile}$ U36C	0.025 ± 0.003
	$tRNA^{Ile}$ U36A	0.029 ± 0.002
	$tRNA^{Ile}$ A35U	0.010 ± 0.004
*Pm*IleRS2	$tRNA^{Ile}$	0.33 ± 0.03
	$tRNA^{Ile}$ U20C	0.47 ± 0.05
	$tRNA^{Ile}$ U21G	0.58 ± 0.09
	$tRNA^{Ile}$ U21C	0.60 ± 0.04
	$tRNA^{Ile}$ G19C	0.46 ± 0.02
	$tRNA^{Ile}$ G34C	0.0033 ± 0.0003
	$tRNA^{Ile}$ U36C	0.0015 ± 0.0005
	$tRNA^{Ile}$ U36A	0.00133 ± 0.00003
	$tRNA^{Ile}$ A35U	0.0025 ± 0.0005

[a]$tRNA^{Ile}$ wild-type and its variants were prepared by *in vitro* transcription.

[b]Observed aminoacylation rate constant under substrate saturation conditions.

Values represent the mean ± SEM of three (n=3) independent experiments. Data from each experiment were fitted individually, and the resulting values were used to calculate the mean ± SEM. Source data are provided as a Source Data file.

Supplementary Table 3. Steady-state kinetic parameters for isoleucine activation by wild-type and mutant *Pm*IleRS2 enzymes[a].

		k_{cat} / s^{-1}	K_M (Ile) / µM	k_{cat} / K_M / s^{-1} μM^{-1}	Relative change[b] k_{cat} / K_M
	WT	50.3 ± 1.9	64.8 ± 3.4	0.78 ± 0.02	1
**ARD1**	R721A	47.4 ± 3.5	54.6 ± 5.8	0.88 ± 0.04	1.1
**ARD3**	R964A	40.6 ± 3.9	61.7 ± 13.6	0.70 ± 0.12	0.9
	ΔINS	13.9 ± 0.88	62.4 ± 1.3	0.23 ± 0.01	0.3

[a] Activation was tested using the [^{32}P]ATP-PP_i exchange assay.

[b] Relative change was defined as the ratio of mutants to wild-type enzyme *Pm*IleRS2.

Values represent the mean ± SEM of three (n=3) independent experiments. Data from each experiment were fitted individually, and the resulting values were used to calculate the mean ± SEM. Source data are provided as a Source Data file.

Supplementary Table 4. Primers used to design *Pm*IleRS2 and *Pm*tRNA^{Ile} GAU mutants.

Primer name	Sequence (5' → 3')
***Pm*IleRS2**	
R721A_F	GCCGATCGTTTTTGGTCAGAAGG
R721A_R	GGAACGTCGCACATACCAG
N647A_F	CTTGTGGCCGTGTACGGCTTTTACG
N647A_R	GTACACGGCCACAAGTGTATCAATGAC
F651A_F	GTACGGCGCTTACGTGCTGT
F651A_R	CGTAAGCGCCGTACACGTTCA
S713A_F	AGCTAGCCAACTGGTATGTGCG
S713A_R	CCAGTTGGCTAGCTCTTCAATGA
Q961A_F	GCAGATTATCGTAAAAAGCTGGATTTACCC
Q961A_R	AACCGCTCGAATTACTTCACG
K965A_F	GCAAAGCTGGATTTACCCGTGAATTC
K965A_R	ACGATAATCTTGAACCGCTCG
R964A_F	TCAAGATTATGCTAAAAAGCTGGATTTACCCG
R964A_R	ACCGCTCGAATTACTTCAC
K862A_F	CGTATTAGCACTGGATTTTAAACAGGCTGG
K862A_R	CCAGTGCTAATACGTATGAAACCAGTTTG
F865A_F	CTGGATGCTAAACAGGCTGGACCAAAGT
F865A_R	GCCTGTTTAGCATCCAGTTTTAATACGTATG
K862A_F865A_F	GGTTTCATACGTATTAGCACTGGATGC
K862A_F865A_R	GCCTGTTTAGCATCCAGTGCTAATACGT
***Pm*tRNA^{Ile} GAU**	
U20G_F	TTATCAGGCGTGCGCTCTACCCAG
U20G_R	GCCTATAGCTCAGCTGGGTAGAG
U21G_F	TTATCAGGCGTGCGCTCTCACCAG
U21G_R	GGCCTATAGCTCAGCTGGTGAGAG
G34C_F	ACCGACCTCACGCTTATGAGG
G34C_R	GTTAGAGCGCACGCCTCATAAGC
A35U_F	ACCGACCTCACGCTTAACAGG
A35U_R	GTTAGAGCGCACGCCTGTTAAGC
U36A_F	ACCGACCTCACGCTTTTCAGG
U36A_R	GTTAGAGCGCACGCCTGAAAAGC
U36C_F	ACCGACCTCACGCTTGTCAGG
U36C_R	GTTAGAGCGCACGCCTGACAAGC
G19C_F	CTAAGCAGCTGAGCTATAG
G19C_R	GCTGCTTAGAGCGCAC

The red nucleotides indicate the mutation sites.

Supplementary References:

1. Modrusan, P. *et al.* Data Repository for 'C-terminal evolutionary remodelling of isoleucyl-tRNA synthetases is a prokaryote-specific strategy for tuning aminoacylation rate'. *Zenodo* (2026) doi:10.5281/zenodo.21453230.
2. Zanki, V., Bozic, B., Mocibob, M., Ban, N. & Gruic-Sovulj, I. A pair of isoleucyl-tRNA synthetases in *Bacilli* fulfills complementary roles to keep fast translation and provide antibiotic resistance. *Protein Sci.* **31**, e4418 (2022).
3. Pope, A. J. *et al.* Characterization of isoleucyl-tRNA synthetase from *Staphylococcus aureus*: II. Mechanism of inhibition by reaction intermediate and pseudomonic acid analogues studied using transient and steady-state kinetics. *J. Biol. Chem.* **273**, 31691–31701 (1999).
4. Hughes, J. & Mellows, G. Interaction of pseudomonic acid A with *Escherichia coli* B isoleucyl-tRNA synthetase. *Biochem. J* **191**, 209–219 (1980).
5. Yanagisawa, T. & Kawakami, M. How does *Pseudomonas fluorescens* avoid suicide from its antibiotic pseudomonic acid? Evidence for two evolutionarily distinct isoleucyl-tRNA synthetases conferring self-defense. *J. Biol. Chem.* **278**, 25887–25894 (2003).
6. Brkic, A. *et al.* Antibiotic hyper-resistance in a class I aminoacyl-tRNA synthetase with altered active site signature motif. *Nat. Commun.* **14**, 5498 (2023).
7. Cvetesic, N. *et al.* Naturally occurring isoleucyl-tRNA synthetase without tRNA-dependent pre-transfer editing. *J. Biol. Chem.* **291**, 8618–8631 (2016).
8. Zivkovic, I., Dulic, M., Kozulic, P., Mocibob, M. & Gruic-Sovulj, I. Kinetic characterization of amino acid activation by aminoacyl-tRNA synthetases using radiolabelled γ-[^{32}P]ATP. *FEBS Open Bio* **15**, 580–586 (2025).
9. Chen, B. *et al.* The mechanism of discriminative aminoacylation by isoleucyl-tRNA synthetase based on wobble nucleotide recognition. *Nat. Commun.* **15**, 10817 (2024).
10. Silvian, L. F., Wang, J. & Steitz, T. A. Insights into editing from an Ile-tRNA synthetase structure with $tRNA^{Ile}$ and mupirocin. *Science* **285**, 1074–1077 (1999).
